# CIBERSORT analysis of TCGA and METABRIC identifies subgroups with better outcomes in triple negative breast cancer

**DOI:** 10.1038/s41598-021-83913-7

**Published:** 2021-02-25

**Authors:** Kelly E. Craven, Yesim Gökmen-Polar, Sunil S. Badve

**Affiliations:** 1 Department of Pathology, Johns Hopkins University School of Medicine, Baltimore, MD 21287 USA; 2 Department of Pathology and Laboratory Medicine, Indiana University School of Medicine, Indianapolis, IN 46202 USA; 3 Department of Medicine, Indiana University School of Medicine, Indianapolis, IN 46202 USA; 4 Indiana University Melvin and Bren Simon Cancer Center, Indianapolis, IN 46202 USA

**Keywords:** Immunoediting, Breast cancer, Cancer genomics, Tumour immunology

## Abstract

Studies have shown that the presence of tumor infiltrating lymphocytes (TILs) in Triple Negative Breast Cancer (TNBC) is associated with better prognosis. However, the molecular mechanisms underlying these immune cell differences are not well delineated. In this study, analysis of hematoxylin and eosin images from The Cancer Genome Atlas (TCGA) breast cancer cohort failed to show a prognostic benefit of TILs in TNBC, whereas CIBERSORT analysis, which quantifies the proportion of each immune cell type, demonstrated improved overall survival in TCGA TNBC samples with increased CD8 T cells or CD8 plus CD4 memory activated T cells and in Molecular Taxonomy of Breast Cancer International Consortium (METABRIC) TNBC samples with increased gamma delta T cells. Twenty-five genes showed mutational frequency differences between the TCGA high and low T cell groups, and many play important roles in inflammation or immune evasion (*ATG2B, HIST1H2BC*, *PKD1, PIKFYVE, TLR3, NOTCH3*, *GOLGB1, CREBBP*). Identification of these mutations suggests novel mechanisms by which the cancer cells attract immune cells and by which they evade or dampen the immune system during the cancer immunoediting process. This study suggests that integration of mutations with CIBERSORT analysis could provide better prediction of outcomes and novel therapeutic targets in TNBC cases.

## Introduction

Several studies have shown that the presence of tumor infiltrating lymphocytes (TILs) in Triple Negative Breast Cancer (TNBC) is associated with a better prognosis^1–8^. This finding is further supported by a recent pooled analysis of nine studies that found improved invasive disease free survival (iDFS), distant disease free survival (D-DFS), and overall survival (OS) with increasing amounts of either intratumoral or stromal lymphocytes in TNBC patients receiving adjuvant chemotherapy^9^. Some studies have attempted to further delineate the specific types of lymphocytes that confer this survival advantage. These have shown that higher counts of CD8 (genes: CD8A, CD8B) T cells are associated with a better prognosis in TNBC^10–17^. For example, Savas et al. used flow cytometry and single-cell RNA sequencing to show that CD8 T cells with memory T cell differentiation (CD103 (gene: ITGAE) positive tissue-resident memory T cells) are associated with improved relapse-free and OS in TNBC patients and that this cell type provides better prognostication than CD8 expression alone^18^. Similarly, studies have shown better prognosis with CD3 (genes: CD3D, CD3E, CD3G) T cells^13,17^, CD4 (gene: CD4) T cells^13,15^, and activated T cells identified by expression of T-bet (gene: TBX21)^19^. One other type of T cell, the regulatory FOXP3 (gene: FOXP3) T cell, has been associated both with good^13,20,21^ and bad prognosis^22^ depending on the study. Other than these few markers, there are a lack of studies looking at additional immune sub-populations in TNBC and their relation to outcomes like OS and disease free survival (DFS).

While most of these studies utilized immunohistochemistry, gene expression data often affords the opportunity to interrogate many more immune cell types. Gene expression signatures have been used to quantify the amount of lymphocyte infiltration in TNBC^23,24^, but only a few studies have used gene expression signatures to quantify specific immune cell types^25–27^. Even fewer studies have attempted to determine the molecular features of the cancer that are associated with the increased immune infiltrate or immune cell type^28^. Karn et al. found that TNBC tumors with high immune gene expression had lower clonal heterogeneity, fewer copy number alterations, lower somatic mutations, and lower neoantigen loads, suggesting that the immune system eliminates some of the diversity seen in immune poor tumors^28^. However, a focus on individual alterations has been lacking.

CIBERSORT is a deconvolution method that characterizes the cell composition of complex tissue from their gene expression profiles^29^. It employs linear support vector regression (SVR), a machine learning approach, to deconvolute a mixture of gene expression. Its results have been shown to correlate well with flow cytometric analysis, and therefore, it has also been referred to as “digital cytometry”^30^. Although this technique has been applied to solid tumors including breast cancers^31–34^, its usage has been relatively limited.

While The Cancer Genome Atlas (TCGA) offers a significant amount of molecular data on TNBC tumors, often underscored with this data source is the availability of hematoxylin and eosin (H&E) images of the tumors. Therefore, we utilized the H&E images to identify TIL rich and TIL poor TNBC tumors, such that further molecular comparisons between the groups could be made. We also used gene expression data to further delineate specific immune cell types and their relation to prognosis. An additional TNBC dataset, Molecular Taxonomy of Breast Cancer International Consortium (METABRIC), was also utilized to determine the reproducibility of our findings.

## Results

**Figure 1:**
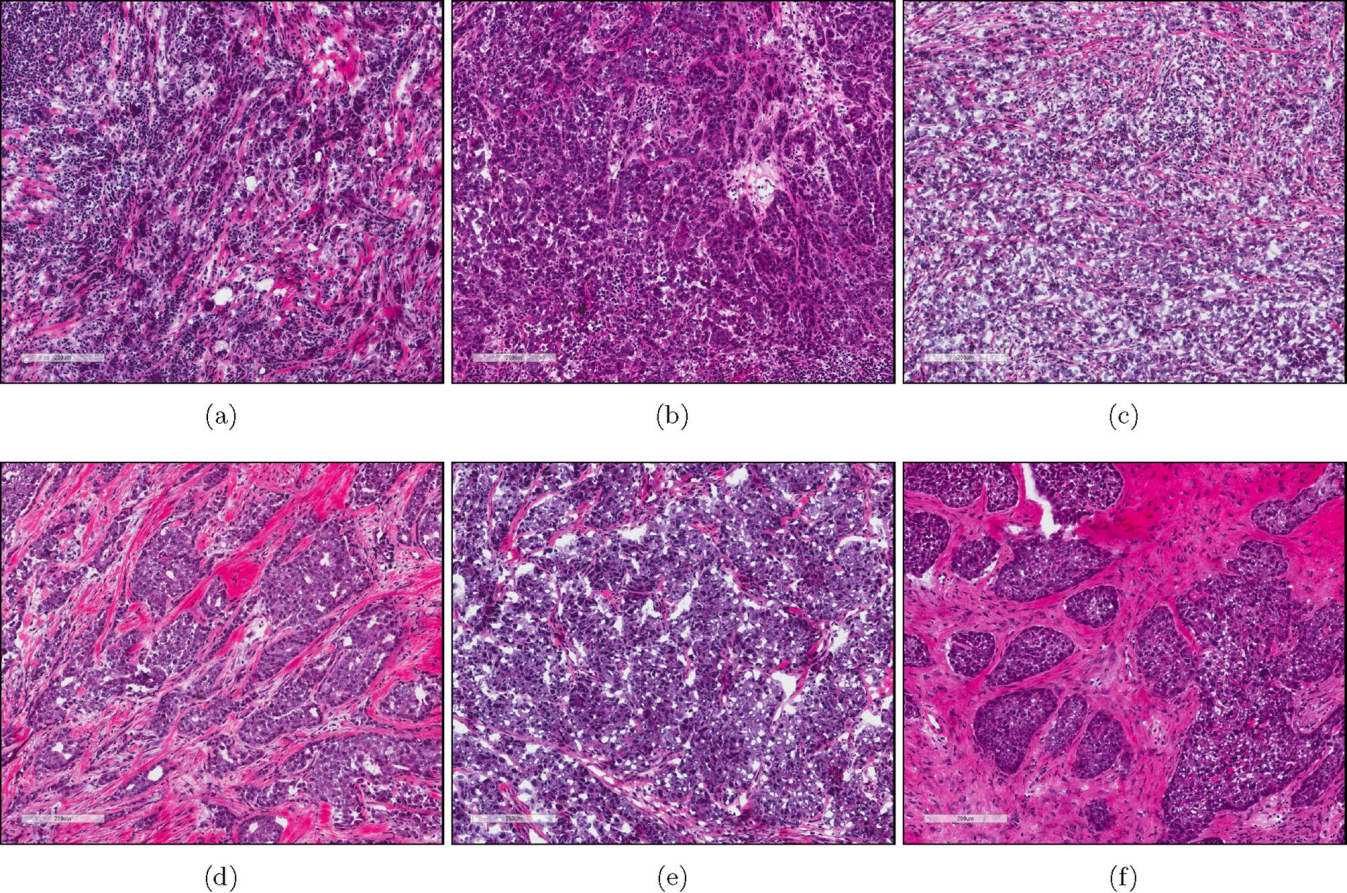
Example H&E images of TCGA TNBC cases with > 30%. (**a**–**c**) or < 30% (**d**–**f**) TILs as scored by a pathologist. (**a**) TCGA-A2-A0CM. (**b**) TCGA-S3-AA10. (**c**) TCGA-EW-A1OV. (**d**) TCGA-LL-A740. (**e**) TCGA-OL-A6VO. (**f**) TCGA-GI-A2C9. (**a**)–(**f**) Image captures made from TCGA image files opened with Aperio ImageScope 12.3.2.8013 (https://www.leicabiosystems.com/digital-pathology/manage/aperio-imagescope/).

Because previous clinical trials have shown an association between lymphocytic infiltrate and good prognosis in TNBC^1,3–5^, we sought to investigate the molecular mechanisms underlying these immune cell differences using the TNBC cases within TCGA (Table 1). Out of 133 TNBC cases with available survival data (Table S10), H&E images from 103 of the cases were scored as having < 1%, 10–20%, 20–30%, 30–40%, 40–50%, 50–60%, 60–70%, or > 70% TILs by a pathologist. Using the TIL cut off for lymphocyte-predominant breast cancer (LPBC) used by Loi et al.^9^, we split the samples into two groups, those with > 30% TILs (n = 11) (Fig. 1a–c) or those with < 30% TILs (n = 92) (Fig. 1d–f).

**Table 1:** Characteristics of TCGA TNBC patients.

Characteristic	TNBC patients (n = 133)	TILs			CD8 T cells (0.25 * sd)			CD4 T cells (0.25 * sd)			CD8/CD4 T cells (cluster analysis)		
		TILs > 30% (n = 11)	TILs < 30% (n = 92)	p	High CD8 T cells (n = 40)	Low CD8 T cells (n = 71)	p	High CD4 memory activated T cells (n = 45)	Low CD4 memory activated T cells (n = 72)	p	High CD8/high CD4 (n = 33)	Low CD8/low CD4 (n = 58)	p
**Mean age at pathologic diagnosis**	54.7	51.6	55.1	0.47*	54.4	54.6	0.60*	53.7	56.2	0.46*	54.2	55.2	0.84*
**Ethnicity**				1.00^#^			0.59^#^			0.18^#^			0.75^#^
Caucasian	77 (58%)	6 (55%)	52 (57%)	Y	24 (60%)	37 (52%)	Y	27 (60%)	39 (54%)	Y	19 (58%)	30 (52%)	Y
African American	45 (34%)	4 (36%)	30 (33%)	Y	14 (35%)	27 (38%)	Y	12 (27%)	29 (40%)	Y	11 (33%)	24 (41%)	Y
NA	11 (8%)	1 (9%)	10 (11%)	Y	2 (5%)	7 (10%)	Y	6 (13%)	4 (6%)	Y	3 (9%)	4 (7%)	Y
**Pathologic stage**				0.43^#^			0.56^#^			**0.02**^#^			0.28^#^
I–II	11 (83%)	11 (100%)	73 (79%)	Y	35 (88%)	57 (80%)	Y	42 (93%)	55 (76%)	Y	30 (91%)	45 (78%)	Y
III–IV	20 (15%)	0 (0%)	16 (17%)	Y	5 (13%)	12 (17%)	Y	2 (4%)	16 (22%)	Y	3 (9%)	11 (19%)	Y
NA	3 (2%)	0 (0%)	3 (3%)	Y	0 (0%)	2 (3%)	Y	1 (2%)	1 (1%)	Y	0 (0%)	2 (3%)	Y
**Menopause**				0.43^#^			0.78^#^			0.73^#^			0.64^#^
Pre	35 (26%)	4 (36%)	21 (23%)	Y	12 (30%)	18 (25%)	Y	13 (29%)	16 (22%)	Y	11 (33%)	15 (26%)	Y
Post	82 (62%)	5 (45%)	59 (64%)	Y	24 (60%)	43 (61%)	Y	27 (60%)	46 (64%)	Y	19 (58%)	34 (59%)	Y
NA	16 (12%)	2 (18%)	12 (13%)	Y	4 (10%)	10 (14%)	Y	5 (11%)	10 (14%)	Y	3 (9%)	9 (16%)	Y

**Bold**: pvalue < 0.05.

TCGA: The Cancer Genome Atlas; TNBC: triple negative breast cancer; TILs: tumor infiltrating lymphocytes; p: pvalue; sd: standard deviation; *: Wilcoxon test; ^#^: Fisher’s exact test; Y: Used in Fisher’s exact test; N: Not used for Fisher’s exact test; NA: missing data.

We observed no differences in OS (pvalue (p) = 0.69) (Figure S1a) or DFS (p = 0.69) (Figure S1b) using the log rank test. Similarly, when TILs were treated as a continuous variable, no differences in OS (hazard ratio (HR) 0.80, 95% confidence interval (CI) 0.52–1.22, p = 0.294) or DFS (HR 0.88, 95% CI 0.60–1.28, p = 0.494) were observed using a univariate cox proportional hazards model (Table 2).

**Table 2:** Prognostic value of immune cell types in TCGA TNBC patients (univariate) (continuous).

Cell type	OS HR	OS 95% CI	OS pvalue	OS FDR	DFS HR	DFS 95% CI	DFS pvalue	DFS FDR
TILs (n = 103)	0.80	0.52 to 1.22	0.294	NA	0.88	0.60 to 1.28	0.494	NA
CD8 T cells (n = 132)	0.0015	3.7e−06 to 0.58	**0.033**	0.26	0.060	0.0016 to 2.22	0.127	0.67
CD4 memory activated T cells (n = 132)	0.0000063	1.7e−11 to 2.26	0.067	0.28	0.0000031	5.0e−11 to 0.19	**0.024**	0.26
M1 macrophages (n = 132)	0.00084	1.1e−06 to 0.65	**0.037**	0.26	0.012	7.7e−05 to 1.89	0.087	0.61

**Bold**: pvalue < 0.05.

TCGA: The Cancer Genome Atlas; TNBC: triple negative breast cancer; TILs: tumor infiltrating lymphocytes OS: overall survival; HR: hazard ratio; FDR: false discovery rate; DFS: disease free survival; CI: confidence interval; NA: not applicable.

**Figure 2:**
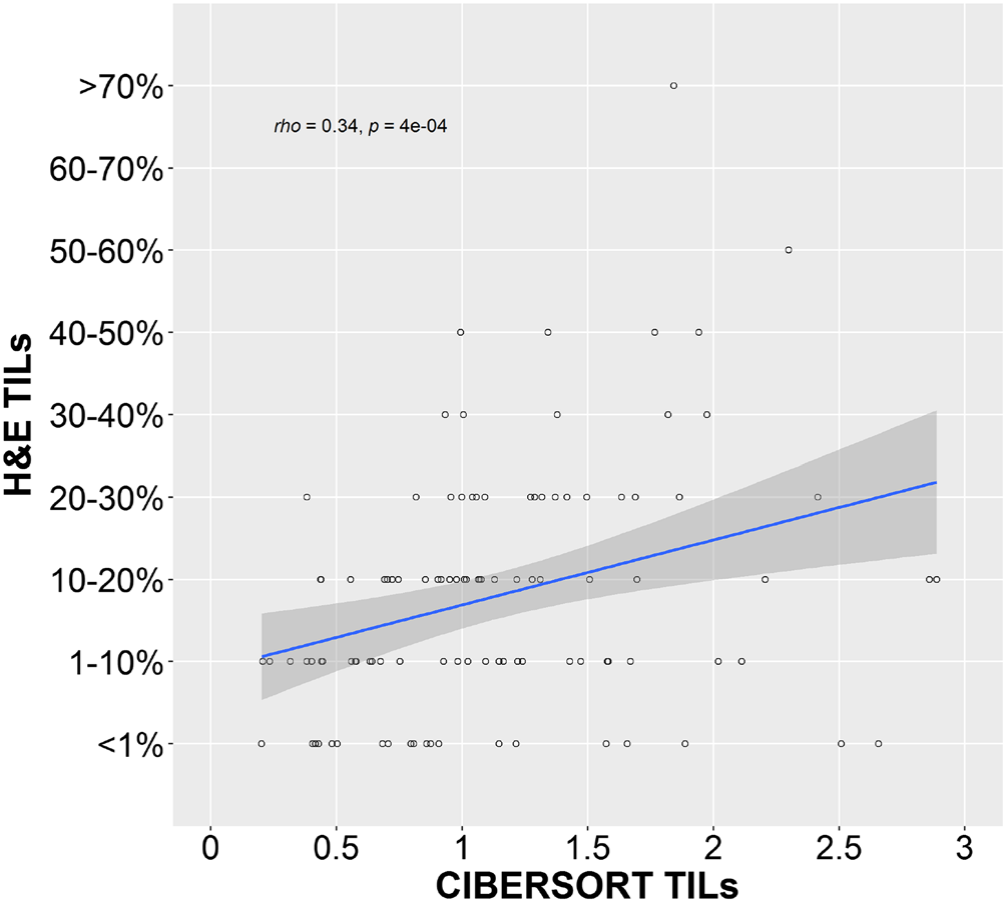
Correlation between H&E scored and CIBERSORT scored TILs in TCGA TNBC cases. Comparison of H&E scored and CIBERSORT scored TILs in TCGA TNBC cases (n = 103) showed a Spearman rank correlation coefficient of 0.34 with a pvalue of 0.0004. The CIBERSORT score consists of arbitrary units that reflect the absolute proportion of immune cells in a mixture. A higher score would indicate a higher proportion of immune cells. Image generated with R 4.0.2 (https://www.R-project.org)^35^.

We hypothesized that differences in survival might only be seen if we focused the analysis on specific immune cell types instead of TILs in general. While immunohistochemistry (IHC) slides for TCGA samples are not a part of the dataset, 132 of the 133 TNBC cases do have RNA-Seq gene expression data. Therefore, the gene expression information from these cases was used to quantify the amount of immune cell infiltrate in each sample by using an application called CIBERSORT, which has pre-identified gene signatures for different immune cell types including B cell subtypes, plasma cells, T cell subtypes, NK cells, monocytes, macrophage subtypes, dendritic cell subtypes, mast cell subtypes, eosinophils, and neutrophils (Figure S2). To determine the validity of CIBERSORT’s deconvolution method, we compared samples that had both H&E scored TILs and CIBERSORT scored TILs and determined the Spearman rank correlation coefficient to be 0.34 (p = 0.0004) (Fig. 2). Of the 22 immune cell types quantified by CIBERSORT, CD8 T cells and M1 macrophages were associated with improved OS while CD4 memory activated T cells were associated with improved DFS (Table 2) using a univariate cox proportional hazards model on a continuous scale. However, the confidence intervals were large due to the small sample size.

**Figure 3:**
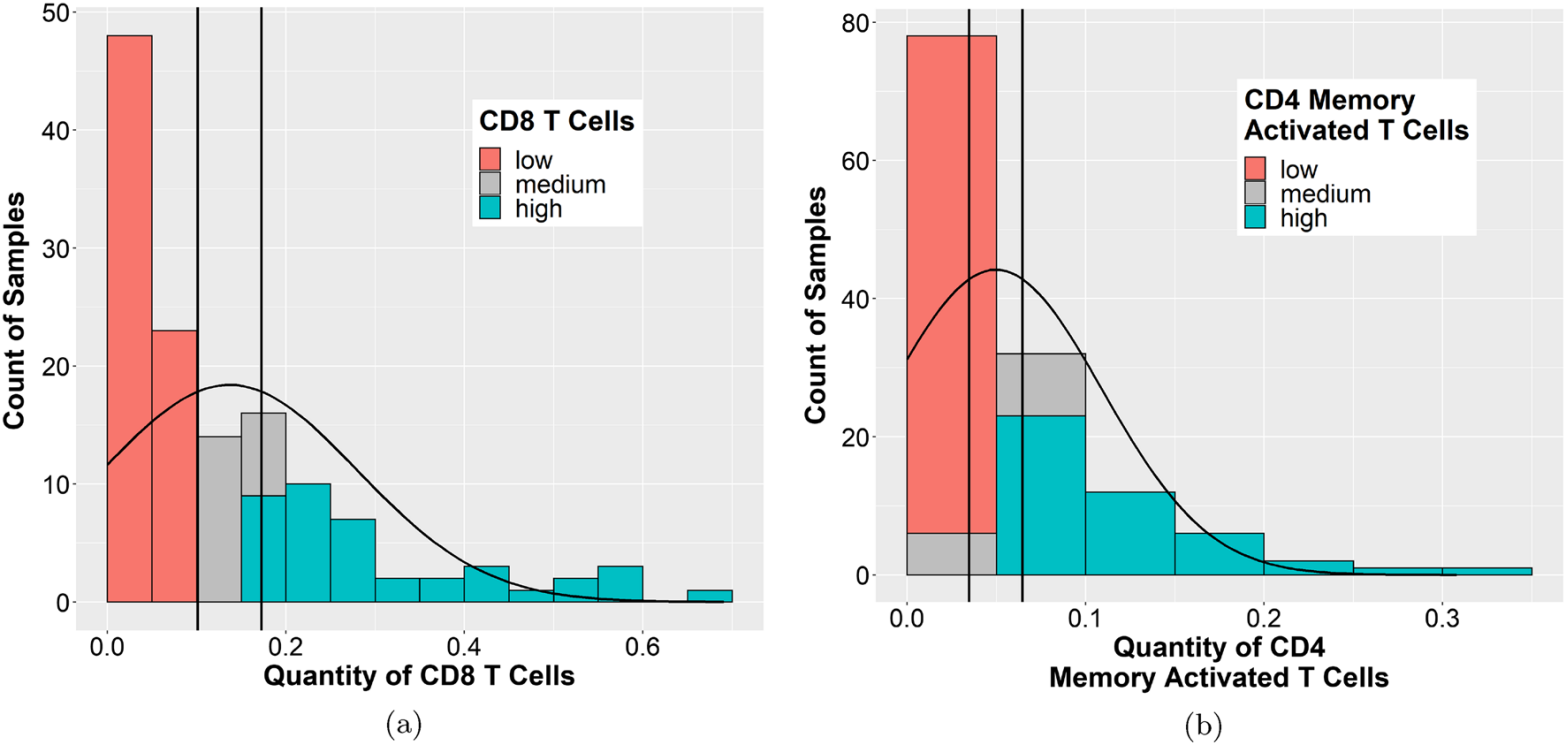
Distribution of the proportion of CD8 T cells or CD4 memory activated T cells across the TCGA TNBC cases. A histogram of the quantity of (**a**) CD8 T cells or (**b**) CD4 memory activated T cells present across the different TCGA TNBC samples was plotted using data generated by CIBERSORT. CIBERSORT assigns a score of arbitrary units that reflects the absolute proportion of each cell type in a mixture. A higher score would indicate a higher proportion of that cell type. Vertical lines represent the cut off (± 0.25 * standard deviation) used to create the “high” and “low” groups. (**a**) and (**b**) Images generated with R 4.0.2 (https://www.R-project.org)^35^.

In order to perform further comparative molecular analysis, we needed to separate the samples into high and low groups for each significant cell type. We tried several different cut offs (Table S1), and only the CD8 T cells and CD4 memory activated T cells showed significance at several different cut offs, while the M1 macrophages did not show significance with any cut off for the OS analysis. Therefore, we decided to focus our analysis on the CD8 T cells and CD4 memory activated T cells. The distribution of CD8 T cells (Fig. 3a) or CD4 memory activated T cells (Fig. 3b) presented a wide range among the different TNBC cases, with many cases having a paucity of immune cells.

**Figure 4:**
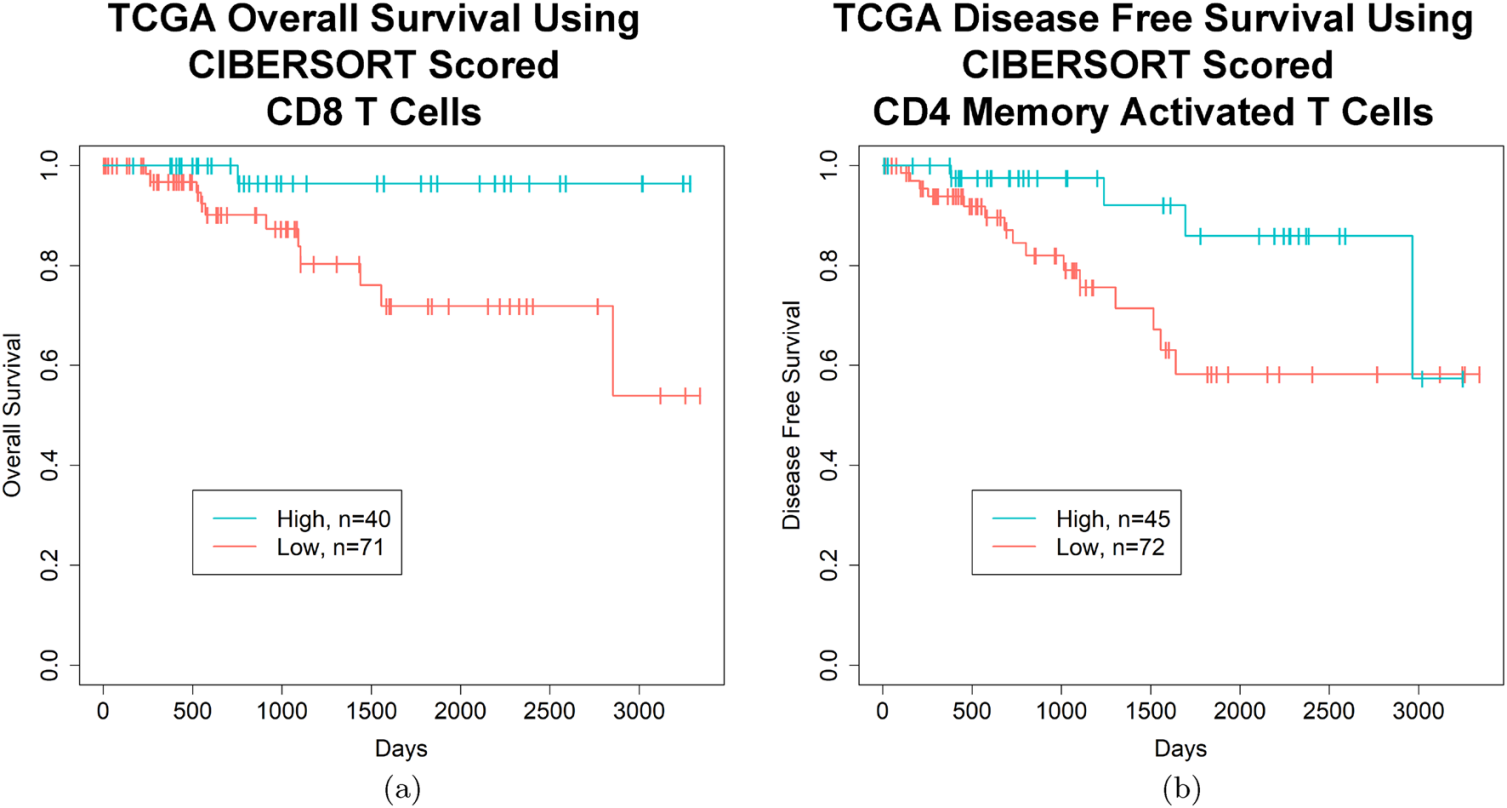
Kaplan–Meier curves demonstrating improvements in OS or DFS in TCGA TNBC patients with high quantities of CD8 T cells or CD4 memory activated T cells in their tumor sample, respectively. (**a**) TCGA TNBC patients with a higher proportion of CD8 T cells in their tumor have a better OS (p = 0.013, FDR = 0.24, log rank test) (survival rates high vs. low, 5 year: 96.4% and 71.9%, 10 year: 96.4% and 53.9%). (**b**) TCGA TNBC patients with a higher proportion of CD4 memory activated T cells in their tumor sample have a better DFS (p = 0.034, FDR = 0.66, log rank test) (survival rates high vs. low, 5 year: 85.9% and 58.2%, 10 year: 57.3% and 58.2%). (**a**) and (**b**) High and low cut offs of T cell infiltrate were chosen as quantities above and below 0.25 times the standard deviation of the mean, respectively. (**a**) and (**b**) Images generated with R 4.0.2 (https://www.R-project.org)^35^.

To separate the TNBC samples into two groups, high vs. low, based on the amount of CD8 T cell or CD4 memory activated T cell infiltrate, a cut off of plus or minus 0.25 times the standard deviation was used for both cell types, disregarding those samples that fell in the middle, termed the medium group (Figs. 3 and S3). After separating the samples into these two groups for each cell type, Kaplan–Meier survival analysis demonstrated that the samples with a high CD8 T cell infiltrate had a better overall survival (p = 0.013, false discovery rate (FDR) = 0.24, log rank test) (Fig. 4a) with 5 year survival rates of 96.4% and 71.9% and 10 year survival rates of 96.4% and 53.9% in the high vs. low groups, respectively (Table S2). Moreover, samples with a high CD4 memory activated T cell infiltrate had a better disease free survival (p = 0.034, FDR = 0.45, log rank test) (Fig. 4b) with 5 year survival rates of 85.9% and 58.2% and 10 year survival rates of 57.3% and 58.2% in the high vs. low groups, respectively (Table S2). Splitting the samples into quartiles showed similar trends (Figure S4a,b), but did not meet the significance threshold.

The significance of the two group analysis of CD8 T cells for OS was retained in a multivariate cox proportional hazards model (p = 0.033), but not the CD4 memory activated T cells for DFS (p = 0.32) (Table 3). This is likely due to correction for the pathologic stage by the model, as the low CD4 memory activated T cell group was biased towards more samples with a higher pathologic stage (p = 0.02)) (Table 1). However, the high and low CD8 T cell groups did not show a bias in any patient characteristic (Table 1).

**Table 3:** Prognostic value of immune cell types in TCGA TNBC (multivariate).

Model	OS HR (95% CI) pvalue (continuous)	OS HR (95% CI) pvalue (high vs. low) (rows 1-5 and 6-10: 0.25 * sd) (rows 11-13: cluster analysis)	DFS HR (95% CI) pvalue (continuous)	DFS HR (95% CI) pvalue (high vs. low) (rows 1-5 and 6-10: 0.25 * sd) (rows 11-13: cluster analysis)
**CD8 T cells**	0.000067 (2.2e−08 to 0.20) **0.019**	0.19 (0.039–0.87) **0.033**	0.067 (7.2e−04 to 6.22) 0.24	0.51 (0.21–1.22) 0.13
Pathologic stage	10.15 (3.51 to 29.35) **1.9e−05**	9.25 (2.71–31.61) **0.00039**	5.90 (2.73 to 12.77) **6.6e−06**	4.95 (1.99–12.36) **0.00060**
Age at pathologic diagnosis	0.91 (0.86 to 0.98) **0.0077**	0.91 (0.85–0.98) **0.0084**	0.95 (0.90 to 1.01) 0.083	0.94 (0.89–1.01) 0.070
Menopause status	2.55 (0.82 to 7.93) 0.11	3.16 (0.88–11.32) 0.077	1.74 (0.61 to 4.97) 0.30	1.77 (0.58–5.37) 0.31
Ethnicity	1.69 (0.50 to 5.71) 0.40	2.16 (0.51–9.20) 0.30	1.66 (0.58 to 4.73) 0.34	2.25 (0.71–7.18) 0.17
**CD4 memory activated T cells**	0.000047 (3.7e−12 to 579.37) 0.23	0.70 (0.21–2.34) 0.56	0.0000054 (4.27e−12 to 6.88) 0.091	0.61 (0.23–1.61) 0.32
Pathologic stage	5.98 (2.39 to 14.98) **0.00013**	7.63 (2.36–24.72) **0.00070**	5.21 (2.45 to 11.11) **0.000019**	7.49 (2.72–20.65) **0.00010**
Age at pathologic diagnosis	0.92 (0.86 to 0.99) **0.017**	0.92 (0.85–1.00) 0.050	0.94 (0.89 to 1.00) 0.052	0.94 (0.88–1.00) 0.064
Menopause status	2.46 (0.70 to 8.64) 0.16	1.33 (0.37–4.71) 0.66	2.01 (0.67 to 6.02) 0.21	1.24 (0.41–3.74) 0.71
Ethnicity	2.34 (0.64 to 8.60) 0.20	1.93 (0.45–8.29) 0.38	1.53 (0.54 to 4.32) 0.42	1.31 (0.43–4.02) 0.64
**CD8/CD4 (cluster analysis)**		0.21 (0.045–0.97) **0.045**		0.41 (0.16–1.07) 0.068
Pathologic stage		6.78 (2.31–19.96) **0.00051**		4.46 (1.84–10.80) **0.00094**
Age at pathologic diagnosis		0.95 (0.90–1.00) 0.053		0.97 (0.92–1.01) 0.14

**Bold**: pvalue < 0.05.

TCGA: The Cancer Genome Atlas; TNBC: triple negative breast cancer; OS: overall survival; HR: hazard ratio; CI: confidence interval; DFS: disease free survival; sd: standard deviation.

**Figure 5:**
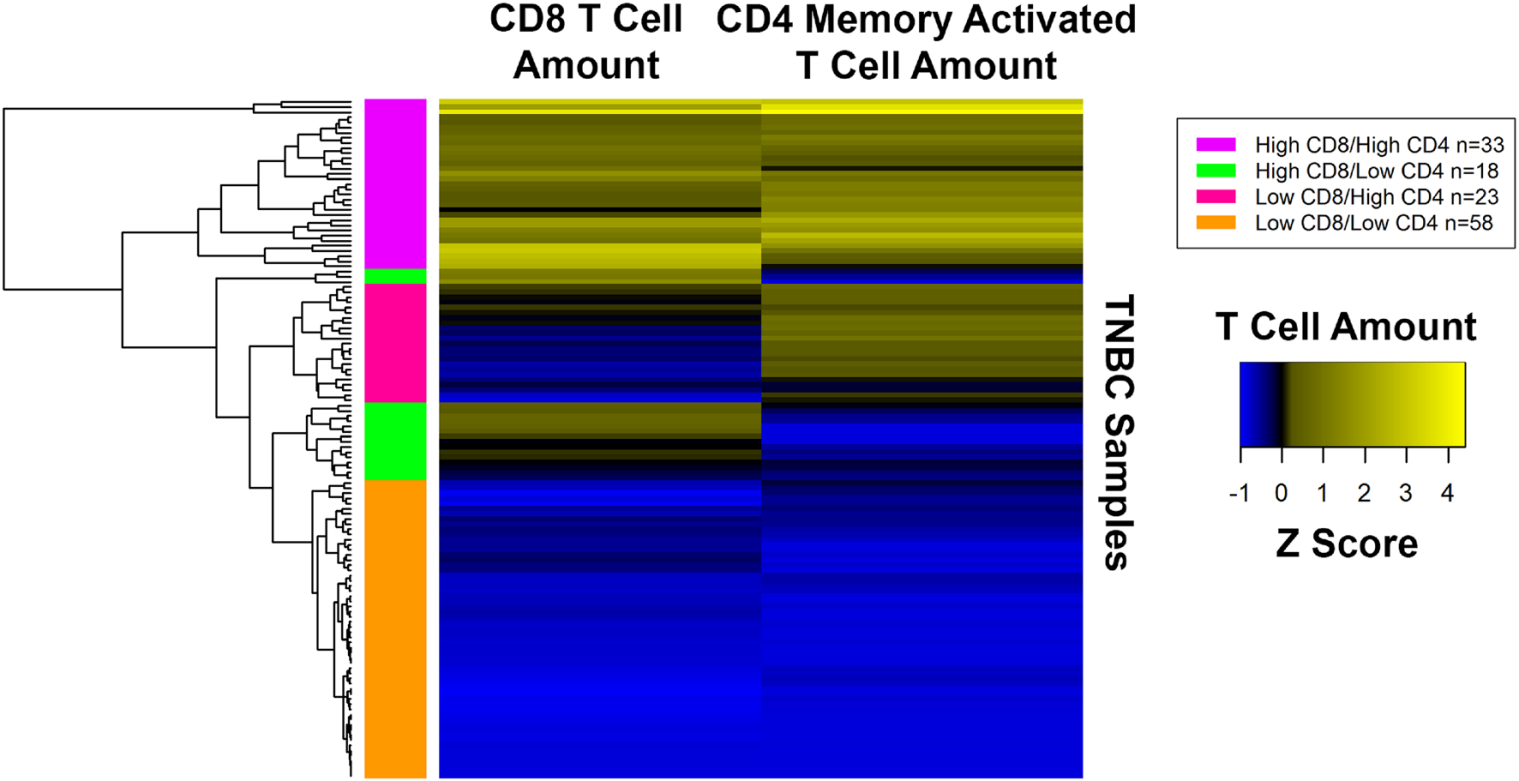
Hierarchical clustering of CD8 T cell and CD4 memory activated T cell quantities in TCGA TNBC samples. RNA sequencing (RNA-Seq) gene expression data was analyzed with CIBERSORT to quantify the amount of different T cells in the TCGA TNBC samples. Hierarchical clustering of these quantities after normalization is shown in the heatmap. Clusters of samples enriched in CD4 memory activated T cells (deep pink cluster), CD8 T cells (green cluster), both cell types (magenta cluster), or neither cell type (orange cluster) are represented. Image generated with R’s (4.0.2) (https://www.R-project.org)^35^ gplots package (3.0.4) (https://CRAN.R-project.org/package=gplots)^36^.

After observing these results, we were curious if the samples with high CD8 T cells also tended to have high CD4 memory activated T cells or if they were mutually exclusive. To determine if the samples had high amounts of both T cell types or if the samples were uniquely enriched in only one T cell type, we did hierarchical clustering of the data (Figs. 5 and S5). Hierarchical clustering indicated that many samples with a high quantity of CD8 T cells also have a high quantity of CD4 memory activated T cells (Fig. 5, magenta cluster). Other clusters indicated that some samples are uniquely enriched in CD8 T cells (Fig. 5, green cluster) or CD4 memory activated T cells (Fig. 5, deep pink cluster), but not both cell types.

**Figure 6:**
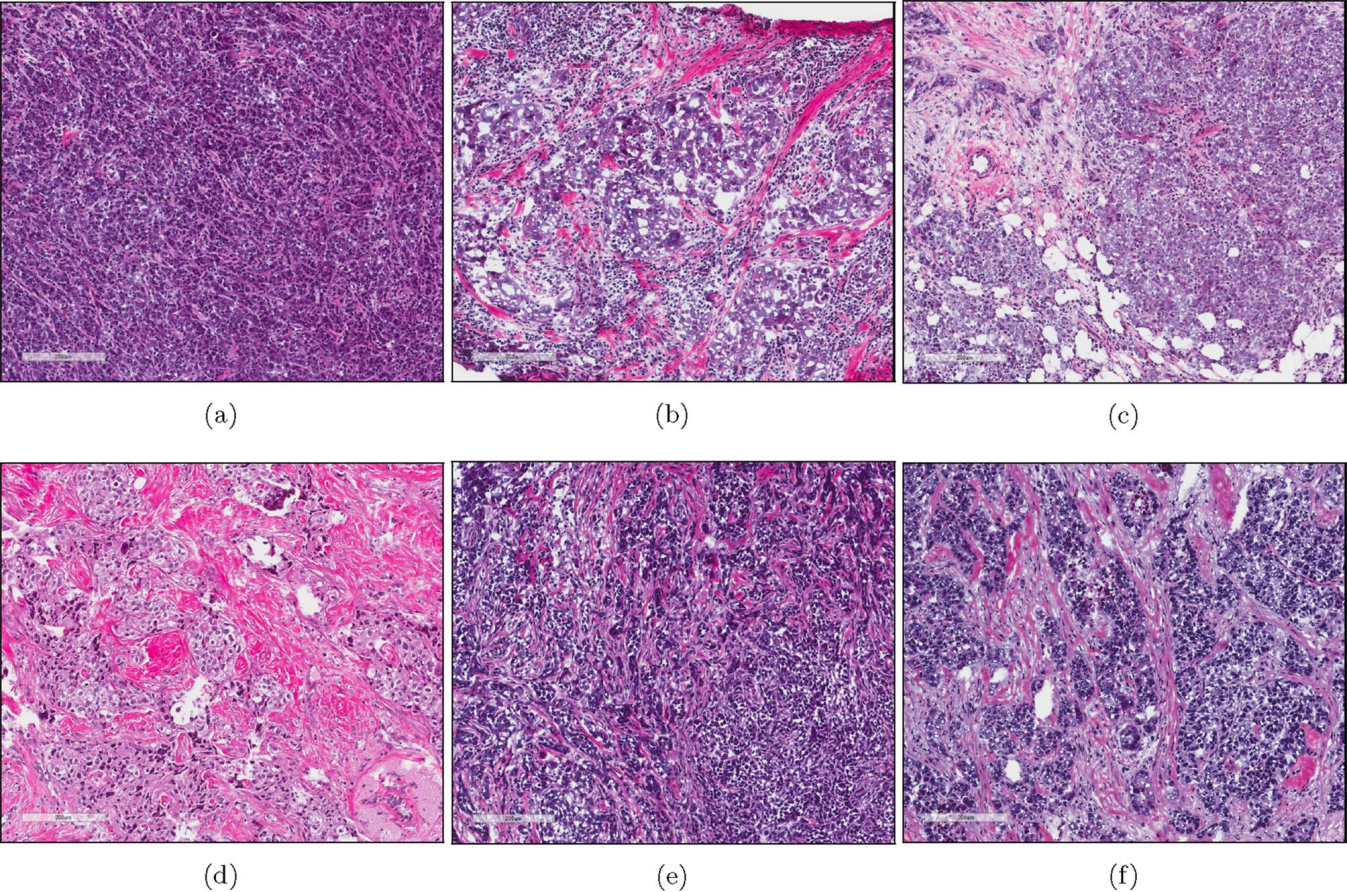
Example H&E images of TCGA TNBC cases with a high quantity of T cells (CD8 and CD4 memory activated) (**a**–**c**) or a low quantity of T cells (CD8 and CD4 memory activated) (**d**–**f**) as scored by RNA-Seq gene expression analysis by CIBERSORT. (**a**) TCGA-AO-A128. (**b**) TCGA-GM-A2DI. (**c**) TCGA-OL-A66I. (**d**) TCGA-A7-A26I. (**e**) TCGA-BH-A0AV. (**f**) TCGA-OL-A5RW. (**a**)–(**f**) Image captures made from TCGA image files opened with Aperio ImageScope 12.3.2.8013 (https://www.leicabiosystems.com/digital-pathology/manage/aperio-imagescope/).

Some representative H&E images of samples identified as having a high quantity of T cells (both CD8 and CD4 memory activated) (Fig. 6a–c) or a low amount of T cells (both CD8 and CD4 memory activated) (Fig. 6d–f) are shown in Fig. 6.

**Figure 7:**
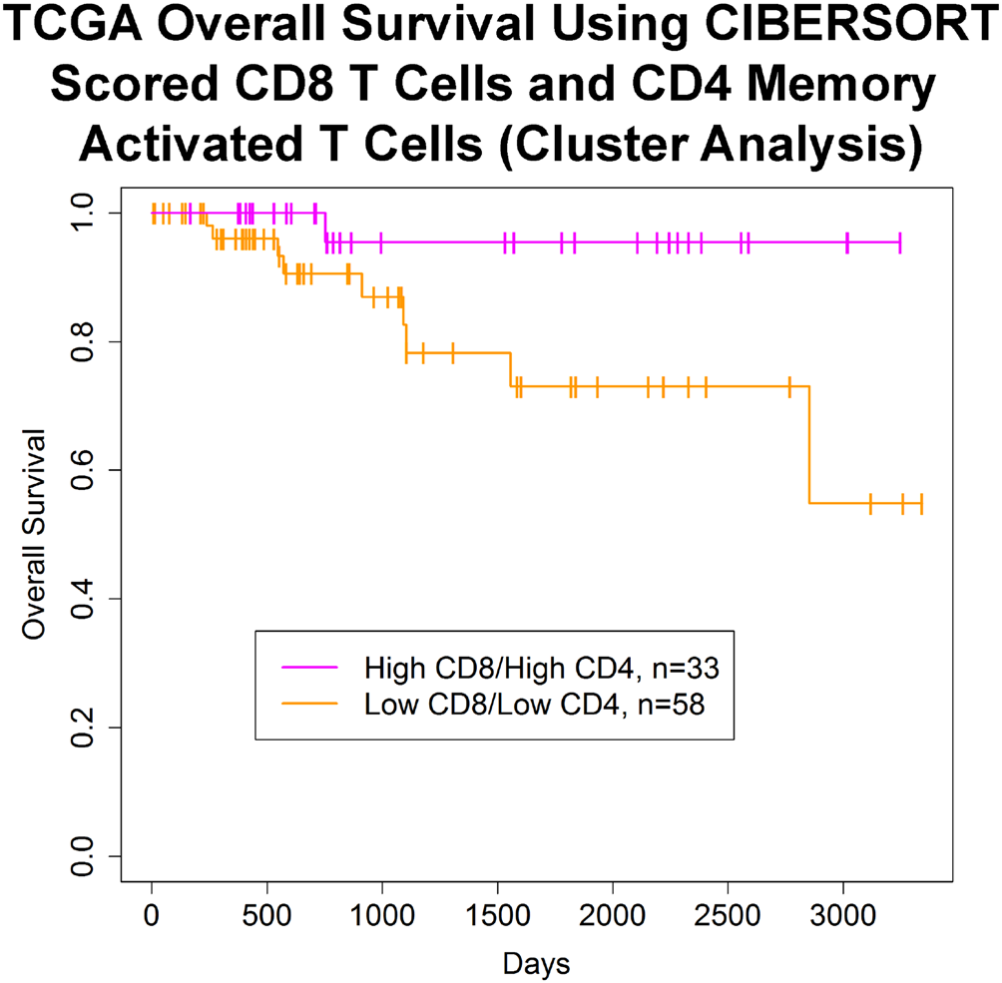
Kaplan–Meier curve demonstrating improvements in OS in TCGA TNBC patients with high quantities of CD8 T cells and CD4 memory activated T cells in their tumor sample. TCGA TNBC patients with a higher amount of CD8 T cells and CD4 memory activated T cells in their tumor as compared to samples with a lower amount of both these cell types have a better OS (p = 0.037, FDR = 0.11, log rank test) (survival rates high vs. low, 5 year: 95.5% and 73.1%, 10 year: 95.5% and 54.8%). The high and low groups were identified according to the hierarchical clustering analysis in Fig. 5. Image generated with R 4.0.2 (https://www.R-project.org)^35^.

To determine if one of the clusters might show an improved prognosis, clusters that had higher amounts of both T cell types (Fig. 5, magenta) or one T cell type (Fig. 5, green, deep pink) were compared individually with the cluster with minimal T cells of either type (Fig. 5, orange) by log rank test (Figures S3, S6). The cluster with higher amounts of both T cell types (Fig. 5, magenta) showed improved OS (Fig. 7, p = 0.037, FDR = 0.11), but not DFS (p = 0.079, FDR = 0.24) while the other individual clusters did not show an improvement in OS or DFS (Figure S6). The survival rates for the high CD8/high CD4 cluster vs. low CD8/low CD4 cluster were 95.5% and 73.1% at 5 years and 95.5% and 54.8% at 10 years, respectively (Table S2). This significance for OS was retained in a multivariate cox proportional hazards model (p = 0.045), but only after backwards elimination of other insignificant covariates (Table 3). To explore whether CD4 memory activated T cells add any additional significance once CD8 T cell information is already known, a likelihood ratio test was done comparing the CD8 T cell continuous model in Table 3 with a second model that differed only in its addition of a CD4 memory activated T cell covariate. This comparison produced a pvalue of 0.61, suggesting that the association with survival is primarily due to the CD8 T cells, and that the CD4 memory activated T cells do not add additional significance.

**Figure 8:**
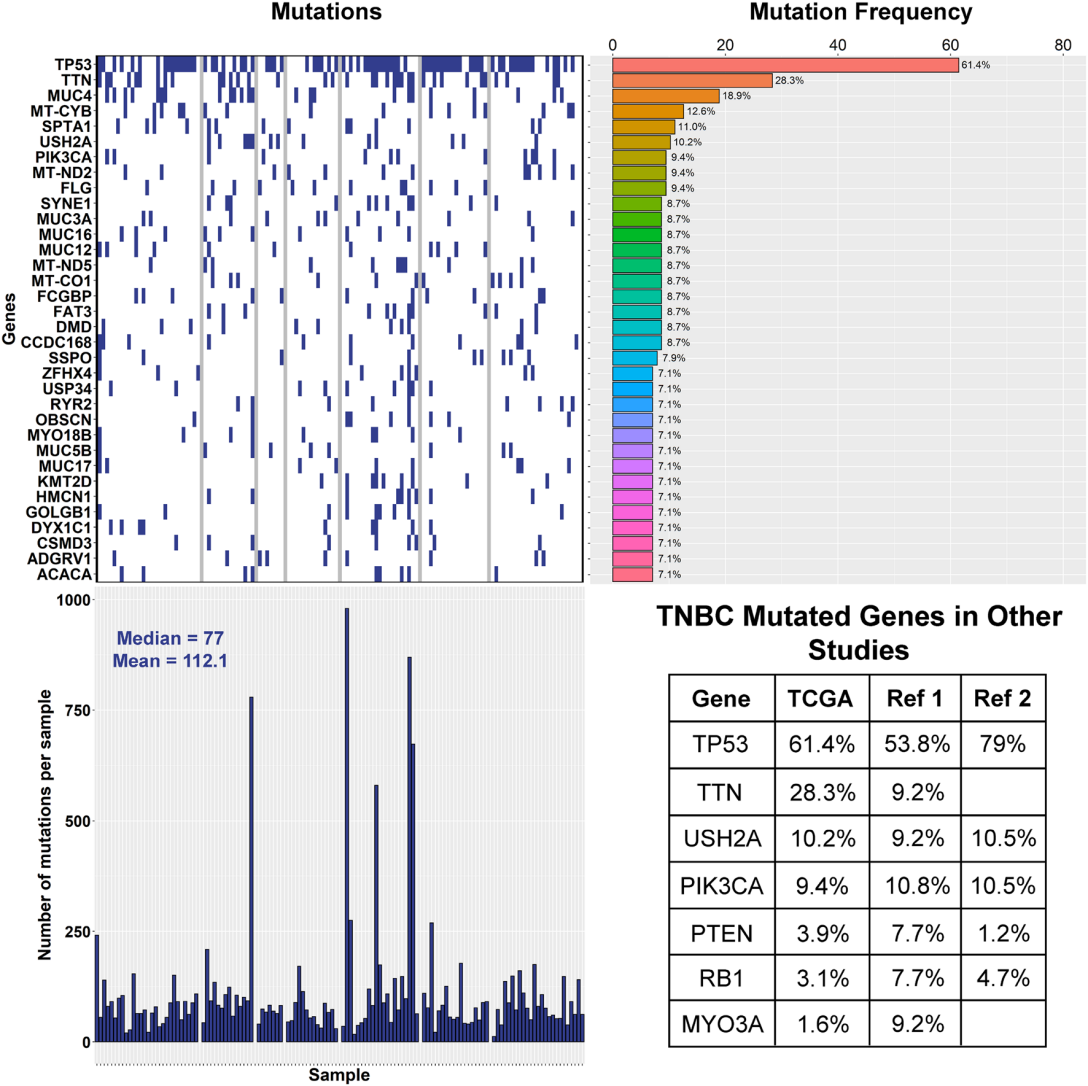
Top 34 gene mutations in TCGA TNBC cases. (Top left and right) Mutational analysis of the TNBC cases in TCGA identifies TP53 (61.4%), TTN (28.3%), MUC4 (18.9%), MT-CYB (12.6%), SPTA1 (11%), and USH2A (10.2%) as the most frequently mutated genes. (Bottom left) Median and mean mutation count are 77 and 112.1, respectively. (Bottom right) Mutation frequencies of several genes in TNBC as determined by this study (TCGA) or other whole genome or exome sequencing studies (Ref 1 =^37^, Ref 2 =^38^). (Top left and right, Bottom left) Images generated with R 4.0.2 (https://www.R-project.org)^35^. (Bottom right) Image generated with Adobe Photoshop CC 2018 (https://www.adobe.com).

To further understand if there is an antigenic characteristic of the tumors that could be driving the differences in immune cell infiltrate, we decided to do a mutational analysis of the TCGA TNBC cases. Overall, looking at the top 34 gene mutations in the TNBC cases in general, the most frequently mutated genes are TP53 (61.4%), TTN (28.3%), MUC4 (18.9%), MT-CYB (12.6%), SPTA1 (11%), and USH2A (10.2%) (Fig. 8, top left and right). Several of these genes, such as TP53 (61.4%), PIK3CA (9.4%), USH2A (10.2%), MYO3A (1.6%), TTN (28.3%), PTEN (3.9%), and RB1 (3.1%), have been previously described in other TNBC whole genome or exome sequencing studies (Fig. 8, bottom right)^37,38^. TNBC cases from TCGA also showed an average of 112.1 and a median of 77 gene mutations per sample (Fig. 8, bottom left).

**Figure 9:**
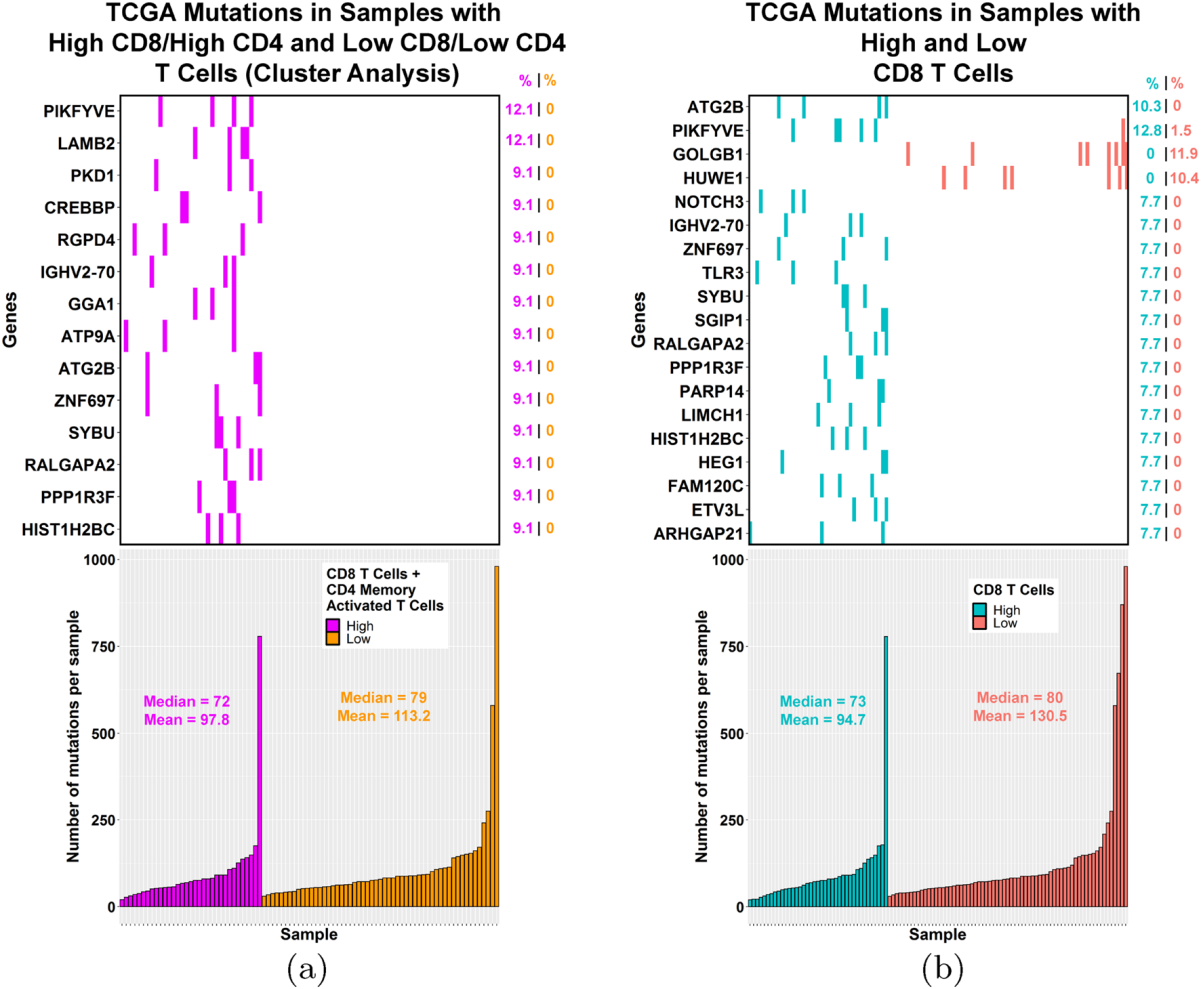
Genes with significantly different mutation frequencies between TCGA TNBC samples with high or low T cells (CD8/CD4 memory activated or CD8). (**a**, top) 14 genes show significantly different mutation frequencies (p < 0.05, Fisher’s exact test) between the group enriched in both CD8 T cells and CD4 memory activated T cells as compared to the group low in these T cells. (**a**, bottom) Mutation counts were similar between the two groups. (**b**, top) 19 genes show significantly different mutation frequencies (p < 0.05, Fisher’s exact test) between the high and low CD8 T cell groups. (**b**, bottom) Mutation counts were similar between the high and low CD8 T cell groups. (**a**) and (**b**) Images generated with R 4.0.2 (https://www.R-project.org)^35^.

Next, we compared the mutation frequencies for all genes between the different groups we identified with improved prognosis. When the group with high CD8 T cells and CD4 memory activated T cells as identified by hierarchical clustering (Fig. 5, magenta) was compared to the cluster group with low amounts of these cells (Fig. 5, orange), 14 genes with significantly different mutation frequencies were identified (Fig. 9a, top) with the mutation frequency ranging from 9.1 to 12.1% in the high group compared to 0% in the low group. For the high vs. low CD8 T cell groups, we found 19 genes with significantly different mutation frequencies (Figure 9b, top). For 17 of these genes, the mutation frequency ranged between 7.7 and 12.8% in the high group compared to 0–1.5% in the low group.

As expected, there was some overlap in the genes identified in the two different comparisons, with phosphoinositide kinase, FYVE-type zinc finger containing (PIKFYVE), immunoglobulin heavy variable 2–70 (IGHV2-70), autophagy related 2B (ATG2B), zinc finger protein 697 (ZNF697), syntabulin (SYBU), Ral GTPase activating protein catalytic alpha subunit 2 (RALGAPA2), protein phosphatase 1 regulatory subunit 3F (PPP1R3F), and histone cluster 1 H2B family member c (HIST1H2BC) more frequently mutated in the high T cell groups compared to the corresponding low groups. However, two genes, golgin B1 (GOLGB1) and HECT, UBA and WWE domain containing E3 ubiquitin protein ligase 1 (HUWE1), did not follow this trend, with no mutations in these genes in the high CD8 group, but mutation frequencies of 11.9% and 10.4% in the low CD8 cases, respectively. While multiple testing correction did not retain any of the significant genes, the goal at this stage of the analysis is exploratory and not to control the number of false positives. Since studies have shown better responses to checkpoint inhibitors when tumor samples possess a higher mutational burden^39^, we also compared the mutation counts between the different high and low groups (Fig. 9a bottom, 9b bottom) and no significant differences were seen.

TNBC is known to be molecularly heterogeneous^40^, and previous studies have utilized gene expression information to further subclassify TNBC samples into various subgroups, such as basal-like (BL1 and BL2), immunomodulatory (IM), mesenchymal (M), mesenchymal stem-like (MSL), and luminal androgen receptor (LAR)^41^. Because the mutation frequencies that we observed did not comprise more than 12.8% of the samples of a group, we decided to subclassify our TNBC samples using a web developed tool called TNBCtype^42^ to determine if samples with the same mutated gene belong to the same subtype within a high or low group. We found that samples with the same mutated genes within a high or low group did not belong to the same TNBC subtype (Tables S3 and S4), but analysis of the high and low groups overall showed that the high groups (CD8 T cells or CD8 T cells combined with CD4 memory activated T cells) were enriched with the immunomodulatory subtype while the corresponding low groups were enriched in the mesenchymal subtype (Table S5).

Lastly, to see if some of these findings would replicate in another dataset, we identified an additional 199 TNBC cases (Tables 4 and S11) from the METABRIC dataset^43,44^ and subjected 196 of them with available OS and gene expression microarray data to CIBERSORT analysis (Figure S7). Of the 22 interrogated immune cell types, only gamma delta T cells were associated with improved OS by a univariate cox proportional hazards model on a continuous scale, while CD8 T cells, CD4 Memory Activated T Cells, and M1 macrophages were not associated with improved OS (Table 5). While many patient demographics were similar between the TCGA and METABRIC datasets (Table S6), more patients in TCGA received chemotherapy compared to METABRIC (77% vs. 59%, respectively) and less patients in TCGA had positive lymph nodes compared to METABRIC (37% vs. 50%, respectively).

**Table 4:** Characteristics of METABRIC TNBC patients.

Characteristic	TNBC patients (n = 199)	CD8 T cells (0.25 * sd)			T cells gamma delta (0.25 * sd)		
		High CD8 T cells (n = 65)	Low CD8 T cells (n = 101)	p	High T cells gamma delta (n = 66)	Low T cells gamma delta (n = 103)	p
**Mean age at pathologic diagnosis**	53.9	53.6	54.7	0.77*	53.0	54.2	0.75*
**Tumor stage**				0.96^#^			0.93^#^
I–II	129 (65%)	42 (65%)	67 (66%)	Y	44 (67%)	65 (63%)	Y
III–IV	12 (6%)	4 (6%)	7 (7%)	Y	4 (6%)	6 (6%)	Y
NA	58 (29%)	19 (29%)	27 (27%)	Y	18 (27%)	32 (31%)	Y
**Inferred menopause**				0.87^#^			0.97^#^
Pre	82 (41%)	28 (43%)	42 (42%)	Y	27 (41%)	43 (42%)	Y
Post	117 (59%)	37 (57%)	59 (58%)	Y	39 (59%)	60 (58%)	Y

METABRIC: Molecular Taxonomy of Breast Cancer International Consortium; TNBC: triple negative breast cancer; p: pvalue; sd: standard deviation; *: Wilcoxon test; ^#^: Fisher’s exact test; Y: Used in Fisher’s exact test; N: Not used for Fisher’s exact test; NA: missing data.

**Table 5:** Prognostic value of immune cell types in METABRIC TNBC (univariate) (continuous).

Cell type	OS HR	OS 95% CI	OS pvalue	OS FDR
CD8 T cells (n = 196)	1.081	0.11–10.89	0.95	1.00
CD4 memory activated T cells (n = 196)	0.091	0.00049–16.98	0.37	0.74
M1 macrophages (n = 196)	0.16	0.013–1.97	0.15	0.59
T cells gamma delta (n = 196)	0.0089	0.00016–0.50	**0.02**	0.48

**Bold**: pvalue < 0.05.

METABRIC: Molecular Taxonomy of Breast Cancer International Consortium; TNBC: triple negative breast cancer; OS: overall survival; HR: hazard ratio; FDR: false discovery rate; CI: confidence interval.

**Figure 10:**
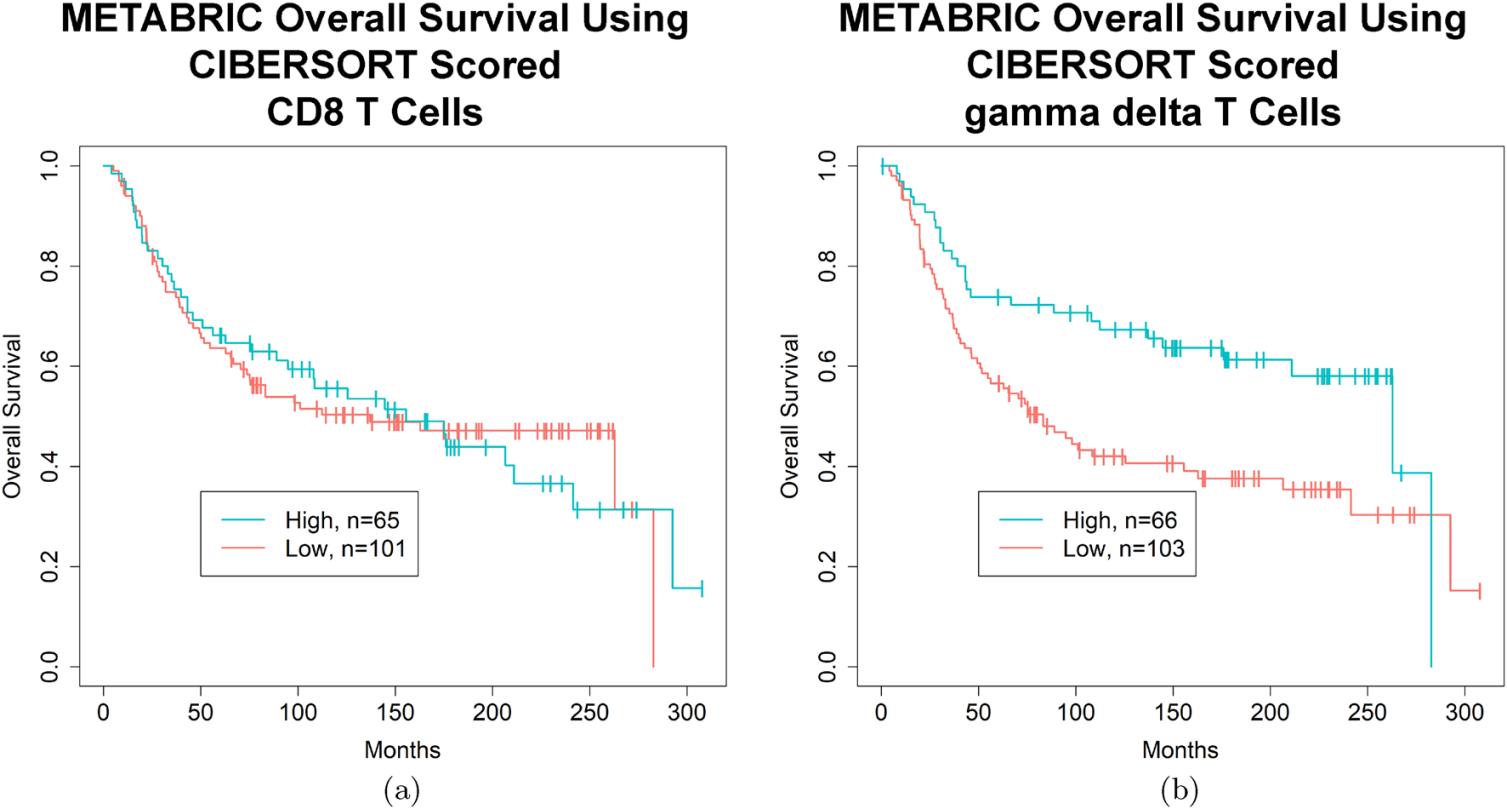
Kaplan–Meier curves demonstrating improvements in OS in METABRIC TNBC patients with high quantities of gamma delta T cells in their tumor sample. (**a**) METABRIC TNBC patients with a higher proportion of CD8 T cells in their tumor do not have a better OS (p = 0.98, FDR = 0.98, log rank test) (survival rates high vs. low, 5 year: 66.2% and 63.5%, 10 year: 55.6% and 50.3%). (**b**) METABRIC TNBC patients with a higher proportion of gamma delta T cells in their tumor sample have a better OS (p = 0.0059, FDR = 0.12, log rank test) (survival rates high vs. low, 5 year: 73.9% and 56.6%, 10 year: 67.3% and 42.0%). (**a**) and (**b**) High and low cut offs of T cell infiltrate were chosen as quantities above and below 0.25 times the standard deviation of the mean, respectively. (**a**) and (**b**) Images generated with R 4.0.2 (https://www.R-project.org)^35^.

The distribution of CD8 T cells (Figure S8a) or gamma delta T cells (Figure S8b) among the different METABRIC TNBC cases was variable, with many cases having a paucity of immune cells. Using a strict cut off of plus or minus 0.25 times the standard deviation to separate the samples into high and low groups recapitulated what was seen in the univariate analysis such that samples with a high CD8 T cell infiltrate did not show a better overall survival (p = 0.98, FDR = 0.98, log rank test) (Fig. 10a) while samples with a high gamma delta T cell infiltrate did have a better OS (p = 0.0059, FDR = 0.12, log rank test) (Fig. 10b, Table S7). For the CD8 T cells, the 5 year survival rate was 66.2% and 63.5%, the 7 year survival rate was 62.9% and 53.9%, and the 10 year survival rate was 55.6% and 50.3% in the high and low groups, respectively (Table S8). For the gamma delta T cells, the 5 year survival rate was 73.9% and 56.6%, the 7 year survival rate was 72.3% and 48.0%, and the 10 year survival rate was 67.3% and 42.0% in the high and low groups, respectively (Table S8). Splitting the samples into quartiles showed similar trends (Figure S9a,b), but the gamma delta T cell group did not quite cross the significance threshold (p = 0.052, FDR = 0.16, log rank test).

Subtype analysis of the METABRIC TNBC samples reproduced what was seen in the TCGA analysis with a high CD8 T cell group enriched in the immunomodulatory subtype and the low group enriched in the mesenchymal subtype (Table S9). While the high gamma delta T cell group was not enriched in any subtype, the low gamma delta T cell group was enriched in the mesenchymal subtype (Table S9).

## Discussion

Most clinical trials evaluating the relationship of TILs to prognosis in TNBC have focused on sub-classifying the TILs into intratumoral vs. stromal TILs^1,3–5^, and these studies generally have observed a survival or relapse benefit when the TILs were evaluated on a continuous scale, such that each 10% increase in TILs is associated with a 11–19% decrease in risk of death or relapse^1,3–6^. Some studies would also utilize a categorical variable of lymphocyte amount, often 50% (termed LPBC), so that specific survival estimates could be reported^1,3,5,6^. In the most recent pooled analysis by Loi et al., stromal TILs (sTILs) were primarily focused on due to their better reproducibility, and the LPBC cut off was set at 30%^9^. In our study, we were unable to reproduce these observations either on the continuous or categorical scale using H&E scored images. We assumed this was because our study was under-powered with only ~130 samples, 103 of which were used for the H&E analysis. Most of these clinical studies analyzed between 250 and 650 samples^1,3,5,6^ with the exception of Loi et al.^4^, which only looked at about 130 samples and thus, they were unable to confirm an association between TILs and OS in that study. Similarly, Park et al. also did not observe an association between TILs and prognosis with only ~120 samples^45^. Lastly, we must consider that survival data in TCGA is not as robust as it would be in a clinical trial.

After we considered that the effect of individual immune cell types might still be substantial enough to show a prognostic benefit in the TCGA dataset, CIBERSORT analysis of gene expression data demonstrated a better OS in those TNBC samples with a higher proportion of CD8 T cells. Additionally, hierarchical clustering identified a subgroup enriched in both CD8 T cells and CD4 memory activated T cells that showed improved OS, though a likelihood ratio test comparing different multivariate models suggests that CD8 T cells mostly contribute to this effect. This is concordant with previous studies that have used immunohistochemistry to associate increased CD8 T cells^10–17^ or CD4 T cells^13,15^ with good prognosis in TNBC. CIBERSORT interrogates three types of CD4 T cells: naive, memory resting, and memory activated. The existence of a memory T cell suggests that the T cell has previously been exposed to an antigen and has been activated^46^. Cell surface markers that can be used to identify memory T cells include high levels of CD44 (gene: CD44), expression of the CD45 (gene: PTPRC) isoform CD45RO (as opposed to the CD45RA isoform in naive cells), and downregulation of CD62L (gene: SELL)^46,47^.

In the process of cancer immunoediting, cancer cells and the immune system go through three phases: elimination, equilibrium, and escape^48^. In the elimination phase, an innate and adaptive immune response (characterized by the propagation of CD4 and CD8 T cells by tumor antigens) work to protect the host against a developing tumor^48^. In the equilibrium phase, an adaptive immune system prevents tumor cell outgrowth, but it may also promote the acquisition of immunoevasive mutations^48^. In the escape phase, tumor cells evade immune recognition and destruction through many different mechanisms^48^. Therefore, tumors with higher intratumoral immune responses with ongoing cancer immunoediting have been found to have better prognosis^48^. Tumors that are more likely to respond to cancer immunoediting include those with a higher mutational burden as they are more likely to express immunogenic neoantigens that can be recognized by T cells^49^. This is also why tumors with high mutational burdens have shown better response rates with immune checkpoint inhibitors^39^.

Breast cancers have a low mutation rate when compared with other solid tumors^50,51^, but TNBC is known to have the highest median mutation rate among breast cancer subtypes, followed next by HER2-positive tumors^39^. This is one possible reason why TILs are more commonly found in TNBC and HER2-positive breast cancers^51^. When comparing TIL rich with TIL poor samples within a breast cancer subtype, one might also expect a higher mutational burden in the TIL rich samples as compared to the TIL poor samples. However, we did not find any significant difference in median mutation count when comparing TNBC tumors with high and low amounts of both CD8 T cells and CD4 memory activated T cells or CD8 T cells alone. Karn et al. actually observed a lower mutational burden in TNBC TIL rich samples as compared to TIL poor samples, and they suggested that this could be due to the elimination process removing immunogenic clones and lowering the clonal heterogeneity^28^.

To better understand the mutational differences in TNBC that might contribute to higher intratumoral immune responses or to reduced immunogenicity and escape, we looked for genes that were frequently mutated in the TIL rich group as compared to the TIL poor group or visa versa, focusing specifically on CD8 T cells or the combination of CD8 T cells with CD4 memory activated T cells. Mutational frequency differences typically ranged from 0% in one group to 7.7–12.8% in the other group. While it would be more convincing to see even higher mutational frequencies within a group, TNBC is known to be molecularly heterogeneous^40^. TNBC subtype analysis did not show samples within a high or low group with similarly mutated genes to be of the same subtype, but the high CD8 T cell or CD8 T cell combined with CD4 memory activated T cell groups were found to be enriched in the immunomodulatory subtype while their corresponding low groups were enriched in the mesenchymal subtype. This finding also repeated in the high and low CD8 T cell groups of the METABRIC TNBC samples. Since the immunomodulatory subtype is defined by immune cell signaling and processes, this association with the high immune groups makes sense. The enrichment of the mesenchymal subtype by the low immune groups, including the METABRIC TNBC low gamma delta T cell group, is interesting and may suggest that active pathways in this subtype may be correlated with a low immune cell infiltrate. The mesenchymal group is defined by pathways involved in cell motility, extracellular matrix receptor interaction, and cell differentiation pathways (such as Wnt, ALK, and TGF-beta)^41^.

Depending on the tumor’s current phase of cancer immunoediting, the mutations we observe in the high TIL group could function by increasing inflammation (elimination phase), by dampening the immune system (equilibrium or escape phases), or by allowing the cancer cells to evade the immune system (equilibrium or escape phases). Therefore, we next explored some of the known roles of the mutated genes that were identified in our analysis on the immune system regardless as to whether it was activating or inhibitory. ATG2B, which was more frequently mutated in the high CD8 T cell group and the group enriched in both T cell types, produces a gene product involved in autophagy, and its downregulation by a microRNA has been shown to be associated with an increased inflammatory response in Crohn’s disease^52^. Likewise, a decrease in the protein produced by HIST1H2BC, which can act as an antimicrobial peptide^53^, has been shown to augment the ability of TNF-alpha (gene: TNF) to upregulate inflammation associated genes^54^. Therefore, mutations in these genes could play a role in the increased inflammatory infiltrate observed in the high CD8 T cell group and the group enriched in both T cell types (Fig. 9a,b).

Polycystin 1, transient receptor potential channel interacting (PKD1), which was more frequently mutated in the group enriched in both T cell types (Fig. 9a), has been shown to play a role in the inflammatory response following toll like receptor 2 (TLR2), toll like receptor 4 (TLR4), or toll like receptor 5 (TLR5) activation^55^. PIKFYVE, frequently mutated in both the high T cell groups (Fig. 9a,b), and toll like receptor 3 (TLR3), frequently mutated in the high CD8 T cell group (Fig. 9b), have both been implicated in the production of type I interferons^56–58^. Studies have also shown that TLR3 is expressed on cancer cells and its activation leads to recruitment of different leukocyte subpopulations^59^. Notch receptor 3 (NOTCH3) was more frequently mutated in the high CD8 T cell group (Fig. 9b); in NOTCH3 deficient mice following tubular kidney injury, monocytic cell infiltration was shown to be reduced, likely due to abrogated chemokine synthesis^60^. Thus, mutations in these genes might lead to a reduced inflammatory response as the tumor evolves.

CREB binding protein (CREBBP), frequently mutated in the group enriched in both T cell types (Fig. 9a), has been implicated in the inflammatory response^61,62^, the control of MHC-II expression^63,64^, and the transactivation of the non-classical MHC-I molecule, HLA-G^65^. While MHC-II molecules are typically expressed by professional antigen-presenting cells, tumors have also been shown to also express them, which may lead to their increased recognition by the immune system^66^. Consequently, mutations in this gene might lead to reduced MHC expression and immune evasion by the cancer cells.

Of interest, mutational data analysis of CD8 immune-cold tumors showed HUWE1 and GOLGB1 mutations to be present only in “cold” tumors (Fig. 9b). Prior independent research shows that HUWE1 plays an important role in cancer tumorigenesis and metastasis, including breast cancer^67^. HUWE1 is a multifunction protein that affects several hallmarks of cancer including proliferation/differentiation, DNA repair, stress response and apoptosis pathways. Its substrates include key regulators of apoptosis, proliferation and differentiation, DNA repair, and stress response. Chen et al. have shown that in TP53-null cells, p14^ARF^ (gene: CDKN2A) induces p53-independent growth suppression by inhibiting HUWE1. In contrast, in TP53 wild type cells, HUWE1 directly binds to and ubiquitinates p53, which suppresses p53-induced apoptosis^68^. Similar opposing effects of ER beta (gene: ESR2) based on mutated and wild type TP53 have been recently described^69,70^. Furthermore, one of the major substrates of HUWE1 is the Myc (gene: MYC) proto-oncogene that can serve as a transcription activator or repressor based on the partners in its transcription complex. Myc-Max complex activates transcription of growth-promoting genes, whereas Myc-Max-Miz1 complex represses transcription through Miz1 (gene: ZBTB17), p300 histone acetyltransferase (gene: EP300) and DNA methyltransferases, DNMT3A and DNMT3B. Another substrate, Miz1, a zinc-finger transcription factor, binds and regulates the expression of several genes, including BCL2^71^, CDKN2B^72^, CDKN1A^73^. CDC6, cell division cycle 6, also a HUWE1 substrate, is a protein essential for the initiation of DNA replication. This protein functions as a regulator at the early steps of DNA replication.

Chen et al. have documented that knockdown of HUWE1 in TNBC cells (TP53 stable and mutant cell lines) was associated with growth arrest, confirming that HUWE1 functions both in TP53-dependent and independent manner^74^. Furthermore, Di Fiore’s group^75^ documented that lack of HUWE1 mRNA expression is associated with poor prognosis in (all subtypes) breast cancer (pvalue < 0.02) using in situ hybridization (ISH) in two cohorts (each ~450 patients)^75^.

GOLGB1 is believed to play a role in endoplasmic reticulum and golgi traffic^76^, and knockdown of this gene has been shown to lead to abnormal glycosylation in prostate cancer cells^77^. Abnormal glycosylation is a known hallmark of cancer^78^ which has been observed in breast cancer^79^, and studies have suggested that abnormal glycosylation might play a role in immune evasion^80^. Perhaps the presence of this mutation early on might contribute to reduced recognition of the cancer cells by the immune system, promoting a low inflammatory environment in the low CD8 T cell group. Overall, identification of these mutations suggests novel mechanisms by which the cancer cells initially attract immune cells, and also novel mechanisms by which they evade or dampen the immune system as they evolve through the cancer immunoediting process.

Unfortunately, the association between CD8 T cells or CD8 T cells in combination with CD4 memory activated T cells and survival outcomes in the TCGA dataset did not reproduce in the analysis of the METABRIC dataset. It is not clear why this is the case, as there is supporting evidence for an association between increased CD8 T cells^10–17^ or CD4 T cells^13,15^ and good prognosis in TNBC in several previous studies. One possible explanation might involve timing, as the TCGA dataset had about 8 years of outcome data while METABRIC had closer to 25 years. For the METABRIC dataset, a bump in the survival curve for the high CD8 T cell group was observed at about the 7 year mark (Fig. 10a) with a 62.9% survival rate compared to a 53.9% survival rate in the high vs. low group, respectively, whereas the TCGA 7 year survival rates were 96.4% and 71.9%, respectively. Moreover, since the start of this study, additional deconvolution methods have been developed^81,82^, some of which have identified possible “spillover” between different cell types, such that there may be limitations in CIBERSORT’s ability to differentiate between similar cell types such as CD8 T cells and gamma delta T cells^83^. While most gamma delta T cells do not express CD8 or CD4, up to 30% can express CD8^84^. These new deconvolution methods aim to reduce dependencies between such closely related cell types. Other possible explanations might involve differing patient demographics (Table S6) or differing technologies for detecting gene expression, as TCGA used RNA-Seq while METABRIC used microarrays.

The additional finding in the METABRIC dataset of an association of gamma delta T cells with better outcomes in TNBC has recently been reported in a much smaller cohort of 11 patients^85^, and other studies have identified such an association with other breast cancer subtypes^33,86^. In vitro or in vivo studies have also shown that gamma delta T cells can be cytotoxic to TNBC cell lines or human xenograft models^87–89^. However, other studies have shown the opposite finding, that gamma delta T cells can be associated with worse outcomes in breast cancer^90^, or that they have an immunosuppressive effect that can lead to breast tumor progression in vitro or in vivo^91,92^. Therefore, additional studies are warranted, especially those specific to TNBC or gamma delta subtypes.

While our study focused on understanding the mutational differences between TNBC tumors with high or low immune cell infiltrate and their possible role in influencing the immune microenvironment, other studies have focused on mutations in TNBC in the context of their ability to produce tumor antigens^39^. Identification of neoantigens holds promise for the use of adoptive cell transfer therapy that directly targets the immunogenic mutations^93^. To date, several immune checkpoint inhibitor trials have been conducted in TNBC, either as a monotherapy or combined with chemotherapy, with modest response rates observed^94^. Because immune checkpoint inhibitors rarely work in tumors devoid of CD8 T cells^94^, it is important to develop ways in which immune cells can be increased within the tumor. Further study of these genes and T cell subtypes is warranted in order to identify ways to augment the immune system or to overcome the immunosuppressive tumor microenvironment in TNBC and lead to better anti-tumor responses.

## Methods

TCGA breast cancer data was downloaded from the genomic data commons (GDC) data portal and legacy archive on May 19th and 20th, 2017. METABRIC breast cancer data was downloaded from cBioPortal^95,96^ on September 2nd, 2020.

### Selection of TNBC cases

#### TCGA

Clinical information on estrogen receptor (ER), progesterone receptor (PR), and human epidermal growth factor receptor 2 (HER2) status was obtained from the clinical biospecimen core resource (BCR) XML files. Out of 1097 files, 220 had a “Negative” ER status (breast_carcinoma_estrogen_receptor_status) and a “Negative” PR status (breast_carcinoma_progesterone_receptor_status). Out of these files, 10 cases with a “Positive” HER2 fluorescence in situ hybridization (FISH) result (lab_procedure_her2_neu_in_situ_hybrid_outcome_type) were removed, leaving 210 cases. For the remaining cases, they were kept if one of the following was true: (1) the HER2 FISH status was “Negative” and the IHC status (lab_procedure_her2_neu_immunohistochemistry_receptor_status) was “Negative,” “Equivocal,” “Indeterminate,” or not available OR (2) the HER2 IHC status (lab_procedure_her2_neu_immunohistochemistry_receptor_status) was “Negative” and the reported IHC level (her2_immunohistochemistry_level_result) was 0, 1+, or not available. This resulted in 157 TNBC cases. Of these cases, only 133 were of the histologic type “Infiltrating Ductal Carcinoma” (Table S10).

Of the 133 cases, 132 had associated RNA-Seq gene expression data (TCGA-AR-A0U1 was the one case without), and 127 had mutations reported in the maf file (TCGA-A8-A07C, TCGA-AO-A129, TCGA-AR-A0U4, TCGA-BH-A0E6, TCGA-D8-A27H, TCGA-EW-A1OW were the cases without).

#### METABRIC

Clinical information was obtained from the data_clinical_patient.txt and data_clinical_sample.txt files. The “patient” file contained 2509 rows which was filtered to 350 rows by selecting those cases with a “Negative” ER_IHC, a “Ductal/NST” HISTOLOGICAL_SUBTYPE, and no “GAIN” in HER2_SNP6. Similarly, the “sample” file contained 2509 rows which was filtered to 277 samples associated with 277 unique patients by selecting those cases with a “Negative” ER_STATUS, a “Negative” PR_STATUS, a “Negative” HER2_STATUS, and a BREAST_CANCER_TYPE_DETAILED of “Breast Invasive Ductal Carcinoma.” Merging of the two files resulted in 199 cases that were present in both files.

Of the 199 cases, 196 had associated microarray (Illumina Human v3 microarray) gene expression information in the data_expression_median.txt file represented as log intensity levels.

### H&E scored TILs

#### TCGA

Out of 133 TNBC cases with available survival data, H&E images from 103 of the cases were scored as having < 1%, 10–20%, 20–30%, 30–40%, 40–50%, 50–60%, 60–70%, or > 70% TILs by a pathologist according to published international guidelines^97,98^.

### Survival data

#### TCGA

For the 133 cases, survival information was obtained from the clinical BCR XML files. For overall survival, any deaths were recorded as events while the latest known follow up date was used for censored observations. For disease free progression, deaths or new tumor events that were not new primaries were counted as events, while the latest known follow up date was used for censored observations.

#### METABRIC

For the 196 cases, overall survival information was provided in the data_clinical_patient.txt file. Any deaths were flagged as events while censored observations included those where the person was still living. No DFS data was available for METABRIC.

### Patient characteristics

#### TCGA

For the 133 TNBC cases, patient characteristics were obtained from the clinical BCR XML files. Some groups with very small numbers were combined with the NA group (missing data) to avoid infinite confidence intervals during the cox proportional hazards analysis. These changes include the following: for ethnicity, 6 Asian samples were recoded as NA and for menopause status, 4 peri-menopausal and 2 indeterminate samples were recoded as NA. The patient characteristics of the different T cell groups were then compared using a Wilcoxon (age at pathologic diagnosis) or Fisher’s exact test (ethnicity, pathologic stage, menopause status).

#### METABRIC

For the 199 TNBC cases, patient characteristics were obtained from the data_clinical_patient.txt file. The patient characteristics of the different T cell groups were compared using a Wilcoxon (age at diagnosis) or Fisher’s exact test (tumor stage, inferred menopause).

#### TCGA vs. METABRIC

For the 133 TCGA TNBC cases, patients with chemotherapy drug names listed under the drug_name field were considered to have received chemotherapy while those with no entry in this field were considered to be of unknown status/not to have received chemotherapy. Patients with a pathologic N stage of N0 (to include N0 i+) were considered to have negative lymph nodes, while patients with an N stage of N1 or above were considered to have positive lymph nodes.

For the 199 METABRIC TNBC cases, patients with or without chemotherapy were indicated in the CHEMOTHERAPY field. Patients with LYMPH_NODES_EXAMINED_POSITIVE equaling 0 were considered to have negative lymph nodes while patients with LYMPH_NODES_EXAMINED_POSITIVE greater than 0 were considered to have positive lymph nodes.

The patient characteristics of the different datasets were then compared using a Wilcoxon (age at pathologic diagnosis) or Fisher’s exact test (ethnicity, pathologic stage, menopause status, chemotherapy, positive lymph node). Not applicable (NA) groups were excluded for all the Fisher’s exact tests (except chemotherapy due to how the data were encoded in TCGA) due to differing reasons for missing data across the different studies.

### Gene expression data

#### TCGA

For the 132 cases with RNA-Seq gene expression data, “Primary Tumor” gene expression files with FPKM expression values associated with 60,483 ensembl ids were available. These values were combined into a single file, and the ensembl ids were associated with their respective HUGO gene symbols using the R 4.0.2^35^ biomaRt library^99,100^. 36,184 ensembl gene ids were associated with 36,169 HUGO gene symbols, with some ensembl gene ids mapping to multiple HUGO gene symbols and some HUGO gene symbols mapping to multiple ensembl gene ids. All records without an associated HUGO gene symbol were removed, resulting in a file with 36,218 rows of data, with some redundant HUGO gene symbols.

#### METABRIC

For the 196 cases with gene expression data, the log2 intensity values were already associated with 24,368 HUGO gene symbols as the Illumina probeset to HUGO gene symbol mapping was already done by cBioPortal.

### Immune cell analysis

#### TCGA

CIBERSORT was used for the immune cell analysis of the TCGA gene expression data^29^. Of the available gene expression values provided by TCGA, FPKM values were used due to their superiority for deconvolution analysis^101^. Because the ensembl to HUGO gene symbol mapping resulted in redundant rows, CIBERSORT will automatically choose the record with the highest mean expression across the mixtures during analysis. The created gene expression file with the 132 cases was uploaded to CIBERSORT as a mixture file, and CIBERSORT was run with the following options: relative and absolute modes together, LM22 signature gene file, 100 permutations, and quantile normalization disabled. 100 permutations were used as the recommended minimum, but the use of 1000 permutations showed no change in the returned absolute proportion of each cell type.

#### METABRIC

CIBERSORT was used for the immune cell analysis of the METABRIC gene expression data^29^. Of the log2 intensity values provided by cBioPortal, the log space was reversed by taking 2 raised to the log2 intensity value for all entries as CIBERSORT requires non-log linear space. The created gene expression file with the 196 cases was uploaded to CIBERSORT as a mixture file, and CIBERSORT was run with the following options: relative and absolute modes together, LM22 signature gene file, 100 permutations, and quantile normalization disabled. 100 permutations were used as the recommended minimum, but the use of 1000 permutations showed no change in the returned absolute proportion of each cell type.

### Correlation

#### TCGA

For the 103 TCGA TNBC cases that were scored by both H&E quantification and CIBERSORT quantification, a correlation test was done using the Spearman method in R 4.0.2^35^. The CIBERSORT TIL score for each sample was determined by adding the values for all immune types except for eosinophils and neutrophils. This is based on published international guidelines^97^ to score all mononuclear cells when quantifying TILs.

### Hierarchical clustering

#### TCGA

For the 132 cases subjected to CIBERSORT analysis, the CD8 T cell and CD4 memory activated T cell values representing the quantity of immune cell infiltrate in each sample were normalized prior to passing the data to the heatmap.2^36^ function (gplots package 3.0.4) in R 4.0.2^35^ to do the clustering. The default cluster and distance functions were used and the scale parameter was set to none since the data were already scaled. The cutree function along with visual trends in the resulting dendrogram were used to identify 4 clusters of interest, a cluster enriched in both T cells types (n = 33), a cluster uniquely enriched in CD8 T cells (n = 18), a cluster uniquely enriched in CD4 memory activated T cells (n = 23), and a cluster low in both T cell types (n = 58). A cluster analysis was also run using values for all immune cell types except for CD4 naive T cells since they had a 0 value in every sample.

### Survival analysis

#### TCGA

103 TNBC cases were scored by a pathologist as having < 1%, 10–20%, 20–30%, 30–40%, 40–50%, 50–60%, 60–70%, or > 70% TILs. TILs were treated as a continuous variable and tested for an association with OS or DFS using a univariate cox proportional hazards model with the Wald test. 11 samples had TILs > 30%, and 92 had TILs < 30%. Based on these two groups, Kaplan–Meier curves were plotted and significance for OS or DFS was tested with a log-rank test or univariate cox proportional hazards model with the Wald test.

For the 132 TNBC cases with gene expression information, the absolute numeric results quantifying the amount of each immune cell type in the samples as output by CIBERSORT were loaded in R 4.0.2^35^ along with the survival information. Significance of each immune cell type treated as a continuous variable was tested for an association with OS or DFS using a univariate cox proportional hazards model with the Wald test. Multiple testing correction was performed using the Benjamini and Hochberg method. CD8 T cells and CD4 memory activated T cells were further tested with a multivariate cox proportional hazards model with the Wald test.

For the CD8 T cells, CD4 memory activated T cells, and M1 macrophages, multiple cut offs were used in order to separate the samples into two groups (0.25 * standard deviation, 0.5 * standard deviation, median, mean) or four groups (quartiles) for each immune cell type. Samples that fell in between the standard deviation cut offs, termed the medium group, were disregarded. Once the samples were separated into two or four groups, Kaplan–Meier curves were plotted and significance of OS or DFS tested with a log-rank test. Multiple testing correction was performed using the Benjamini and Hochberg method. Significance at the chosen cut off (0.25 * standard deviation) for the CD8 T cell and CD4 memory activated T cells was also tested with a multivariate cox proportional hazards model with the Wald test.

For the 4 clusters of samples identified by hierarchical clustering, significance of OS or DFS was tested with a log-rank test by comparing each cluster to the cluster low in both T cell types or by using all 4 clusters. Multiple testing correction was performed using the Benjamini and Hochberg method. Kaplan–Meier curves were plotted and a multivariate cox proportional hazards model was run with the Wald test.

#### METABRIC

For the 196 TNBC cases with gene expression information, the absolute numeric results quantifying the amount of each immune cell type in the samples as output by CIBERSORT were loaded in R 4.0.2^35^ along with the survival information. Significance of each immune cell type treated as a continuous variable was tested for an association with OS using a univariate cox proportional hazards model with the Wald test. Multiple testing correction was performed using the Benjamini and Hochberg method.

For the CD8 T cells and gamma delta T cells, multiple cut offs were used in order to separate the samples into two groups (0.25 * standard deviation, 0.5 * standard deviation, median, mean) or four groups (quartiles) for each immune cell type. Samples that fell in between the standard deviation cut offs, termed the medium group, were disregarded. Once the samples were separated into two or four groups, Kaplan–Meier curves were plotted and significance of OS tested with a log-rank test. Multiple testing correction was performed using the Benjamini and Hochberg method.

### Mutation analysis

#### TCGA

127 TNBC cases were found to have a reported mutation in the maf file, so this total number of cases was used when calculating mutation frequencies overall. Of the several maf files generated by TCGA using different variant callers, the protected MUSE maf file was used. Of all the reported mutations, variants with a predicted “HIGH” or “MODERATE” impact on the gene product were kept. Next, if a sample had multiple “HIGH” or “MODERATE” impact mutations in a single gene, then the gene was only considered to be mutated or not in that sample. This means that genes with only “LOW” or “MODIFIER” impact mutations would be called as not being mutated. Therefore, mutation counts are considered to be the number of genes that are mutated. Mutation counts between groups were compared using a two-sample Wilcoxon test.

Of the 33 cases in the cluster enriched for both T cell types and the 58 cases in the cluster low in both T cell types, 33 and 55 had mutation data, respectively. Of the 40 high and 71 low CD8 T cell cases, 39 and 67 had mutation data, respectively. Mutation frequencies between these groups were compared using a Fisher’s exact test. Multiple testing correction was performed using the Bonferroni and Benjamini and Hochberg methods.

### TNBC subtype analysis

#### TCGA

For the 132 TNBC cases with gene expression information, the FPKM values were normalized with R’s scale function (R 4.0.2^35^) and input in the the TNBCtype tool^42^. The tool interpreted 8 samples (labeled NA in Table S10) as having high ER expression and recommended their removal. These samples were removed and the tool was rerun. The resulting classifications were then used to compare the different T cell groups using a Fisher’s exact test. All subtypes were tested at once in a 2 × 8 contingency table, and then each subtype was tested individually with a 2 × 2 contingency table where samples were grouped as belonging to the subtype or not. Multiple testing correction was performed using the Benjamini and Hochberg method.

#### METABRIC

For the 196 TNBC cases with gene expression information, the intensity values were normalized with R’s scale function (R 4.0.2^35^) and input in the the TNBCtype tool^42^. The tool interpreted 9 samples (labeled NA in Table S11) as having high ER expression and recommended their removal. These samples were removed and the tool was rerun. The resulting classifications were then used to compare the different T cell groups using a Fisher’s exact test. All subtypes were tested at once in a 2 × 8 contingency table, and then each subtype was tested individually with a 2 × 2 contingency table where samples were grouped as belonging to the subtype or not. Multiple testing correction was performed using the Benjamini and Hochberg method.

### Code sharing

The relevant code and processed data associated with this project are made available at the following GitHub repository: https://github.com/kelgalla/tnbctils or 10.5281/zenodo.4542590.

## Supplementary Information

Supplementary Information.
