# Supplementary material for: CIBERSORT analysis of TCGA and METABRIC identifies subgroups with better outcomes in triple negative breast cancer
Source: Sci Rep. 2021 Feb 25;11:4691. doi: 10.1038/s41598-021-83913-7 (PMC7907367; doi:10.1038/s41598-021-83913-7)
Supplement: Supplementary file 1 — Supplementary Information. [file 41598_2021_83913_MOESM1_ESM.pdf]

CIBERSORT Analysis of TCGA and METABRIC Identifies Subgroups with Better Outcomes in Triple Negative Breast Cancer

Kelly E. Craven[1], Yesim Gökmen-Polar[2], and Sunil S. Badve[2,3,4,*]

[1]*Department of Pathology, Johns Hopkins University School of Medicine, Baltimore, MD 21287*
[2]*Department of Pathology and Laboratory Medicine, Indiana University School of Medicine, Indianapolis, IN 46202*
[3]*Department of Medicine, Indiana University School of Medicine, Indianapolis, IN 46202*
[4]*Indiana University Melvin and Bren Simon Cancer Center, Indianapolis, IN 46202*
[*]*Corresponding author: sbadve@iupui.edu*

Supplemental material

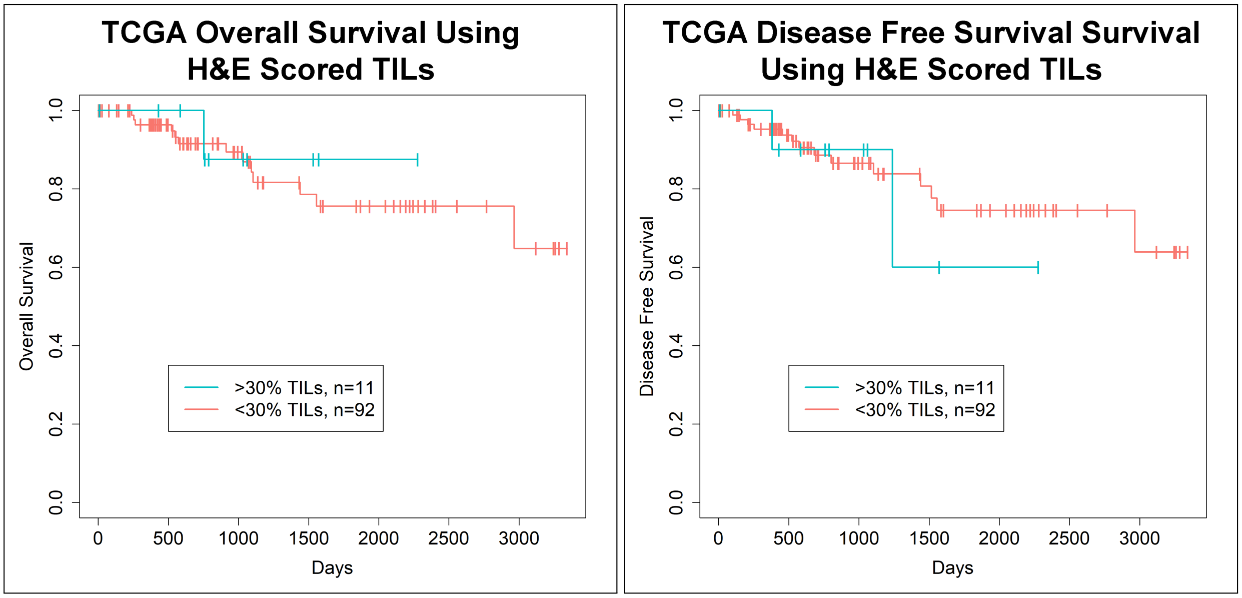

(a) (b)

Figure S1: **Survival data from TCGA does not replicate clinical trial observations.** Kaplan-Meier curves showing no improvement in **(a)** OS (p = 0.69, log rank test) or **(b)** DFS (p = 0.69, log rank test) in TCGA TNBC patients with >30% TILs as compared to <30% TILs as scored by a pathologist. **(a)** and **(b)** Images generated with R 4.0.2 (https://www.R-project.org) [35].

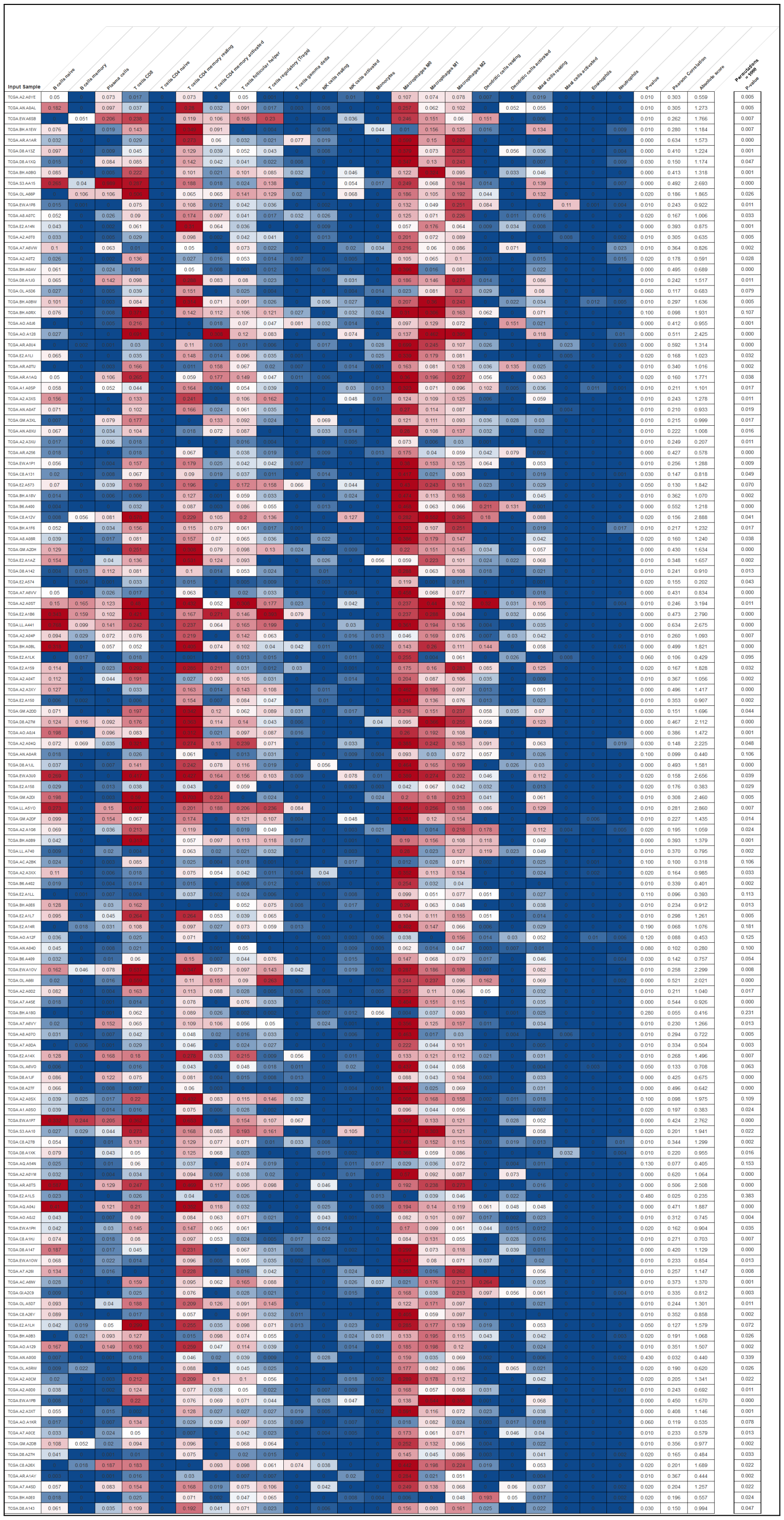

Figure S2: **Quantification of immune cell infiltrate by CIBERSORT analysis of RNA-Seq gene expression data from TCGA TNBC cases.** The heatmap returned by CIBERSORT shows the quantities of 22 different immune cell types among the 132 TCGA TNBC samples. CIBERSORT assigns a score of arbitrary units that reflects the absolute proportion of each cell type in a mixture. A higher score would indicate a higher proportion of that cell type. The far right column indicates the pvalue when 1000 permutations are used instead of 100. Image generated with CIBERSORT (https://cibersort.stanford.edu) [29].

Table S1: Prognostic Value of Immune Cell Types in TCGA TNBC Patients (Kaplan-Meier log rank test)

Cell Type	Cut Off	Low Cut Off	High Cut Off	Low Number	High Number	OS pvalue	OS FDR	DFS pvalue	DFS FDR
CD8 T Cells	0.25*sd	0.1011	0.1726	71	40	**0.013**	0.24	0.077	0.73
CD8 T Cells	median	0.0849	0.0849	66	66	**0.028**	0.14	0.088	0.28
CD8 T Cells	mean	0.1369	0.1369	82	50	**0.028**	0.28	0.176	0.75
CD8 T Cells	0.5*sd	0.0654	0.2084	55	31	**0.032**	0.36	0.158	0.68
CD8 T Cells	quartiles	0.0000, 0.0290, 0.0849	0.0849, 0.1916, 0.6908	33, 33	33, 33	0.094	0.48	0.280	0.54
CD4 Memory Activated T Cells	0.25*sd	0.0348	0.0646	72	45	0.105	0.45	**0.034**	0.66
CD4 Memory Activated T Cells	median	0.0258	0.0258	66	66	**0.038**	0.14	**0.011**	0.11
CD4 Memory Activated T Cells	mean	0.0497	0.0497	78	54	0.270	0.71	0.179	0.75
CD4 Memory Activated T Cells	0.5*sd	0.0199	0.0794	61	33	0.091	0.37	**0.045**	0.29
CD4 Memory Activated T Cells	quartiles	0.0000, 0.0007, 0.0258	0.0258, 0.0789, 0.3078	33, 33	33, 33	0.220	0.48	0.085	0.42
M1 Macrophages	0.25*sd	0.1038	0.1556	60	41	0.210	0.50	0.194	0.74
M1 Macrophages	median	0.1109	0.1109	66	66	0.100	0.26	0.165	0.37
M1 Macrophages	mean	0.1297	0.1297	76	56	0.053	0.28	0.166	0.75
M1 Macrophages	0.5*sd	0.0779	0.1815	50	27	0.056	0.36	**0.032**	0.29
M1 Macrophages	quartiles	0.0000, 0.0599, 0.1109	0.1109, 0.1760, 0.5524	33, 33	33, 33	0.146	0.48	0.114	0.42

TCGA: The Cancer Genome Atlas; TNBC: triple negative breast cancer; OS: overall survival; DFS: disease free survival; sd: standard deviation; FDR: false discovery rate; **Bold**: pvalue < 0.05

Table S2: Survival Rates of TCGA TNBC Patients (Kaplan-Meier)

Years	OS High CD8 T Cells (0.25*sd)	OS Low CD8 T Cells (0.25*sd)	DFS High CD4 Memory Activated T Cells (0.25*sd)	DFS Low CD4 Memory Activated T Cells (0.25*sd)	OS High CD8/High CD4 T Cells (cluster analysis)	OS Low CD8/Low CD4 T Cells (cluster analysis)
5	96.4%	71.9%	85.9%	58.2%	95.5%	73.1%
7	96.4%	71.9%	85.9%	58.2%	95.5%	73.1%
10	96.4%	53.9%	57.3%	58.2%	95.5%	54.8%

TCGA: The Cancer Genome Atlas; TNBC: triple negative breast cancer; OS: overall survival; DFS: disease free survival; sd: standard deviation

Table S3: Subtype Classification of TCGA TNBC Samples with Mutation Frequency Differences: High vs. Low CD8/CD4 T Cells (cluster analysis)

Samples	Gene Mutation	High or Low Group	TNBC Subtypes
TCGA.EW.A3U0, TCGA.D8.A27M, TCGA.C8.A26X, TCGA.A2.A0CM	PIKFYVE	high	UNS, UNS, LAR, IM
TCGA.S3.AA10, TCGA.EW.A1PB, TCGA.GM.A3XL, TCGA.GM.A2DD	LAMB2	high	IM, IM, IM, LAR
TCGA.EW.A3U0, TCGA.GM.A3XL, TCGA.OL.A5D7	PKD1	high	UNS, IM, IM
TCGA.AO.A128, TCGA.AR.A0TS, TCGA.AQ.A04J	CREBBP	high	IM, BL1, MSL
TCGA.EW.A1PB, TCGA.E2.A1L7, TCGA.BH.A0RX	RGPD4	high	IM, NA, NA
TCGA.D8.A27M, TCGA.OL.A66P, TCGA.E2.A1B6	IGHV2-70	high	UNS, LAR, IM
TCGA.D8.A27M, TCGA.C8.A26X, TCGA.GM.A2DD	GGA1	high	UNS, LAR, LAR
TCGA.D8.A27M, TCGA.E2.A1L7, TCGA.A2.A0SX	ATP9A	high	UNS, NA, BL2
TCGA.AO.A128, TCGA.GM.A2DH, TCGA.AR.A1AQ	ATG2B	high	IM, UNS, IM
TCGA.AO.A128, TCGA.A2.A04T, TCGA.AR.A1AQ	ZNF697	high	IM, BL1, IM
TCGA.E2.A159, TCGA.EW.A1OV, TCGA.A2.A04T	SYBU	high	BL2, UNS, BL1
TCGA.AO.A128, TCGA.EW.A3U0, TCGA.OL.A66P	RALGAPA2	high	IM, UNS, LAR
TCGA.D8.A27M, TCGA.GM.A3XL, TCGA.LL.A441	PPP1R3F	high	UNS, IM, MSL
TCGA.E2.A159, TCGA.EW.A1OV, TCGA.GM.A2DI	HIST1H2BC	high	BL2, UNS, NA

TCGA: The Cancer Genome Atlas; TNBC: triple negative breast cancer; sd: standard deviation; BL1: basal-like 1; BL2: basal-like 2; IM: immunomodulatory; M: mesenchymal, MSL: mesenchymal stem-like, LAR: luminal androgen receptor; UNS: unstable; NA: not applicable

Table S4: Subtype Classification of TCGA TNBC Samples with Mutation Frequency Differences: High vs. Low CD8 T Cells (0.25*sd)

Samples	Gene Mutation	High or Low Group	TNBC Subtypes
TCGA.AO.A128, TCGA.GM.A2DH, TCGA.S3.AA15, TCGA.AR.A1AQ	ATG2B	high	IM, UNS, MSL, IM
TCGA.D8.A1XK, TCGA.EW.A3U0, TCGA.D8.A27M, TCGA.BH.A0BG, TCGA.C8.A26X, TCGA.A2.A0CM	PIKFYVE	low	BL1, UNS, UNS, UNS, LAR, IM
TCGA.BH.A18G, TCGA.D8.A1XK, TCGA.C8.A26Y, TCGA.A1.A0SO, TCGA.OL.A5RW, TCGA.D8.A147, TCGA.BH.A0BL, TCGA.A2.A3XX	GOLGB1	low	UNS, BL1, LAR, UNS, UNS, BL1, UNS, BL1
TCGA.BH.A18G, TCGA.D8.A1XQ, TCGA.A1.A0SO, TCGA.AN.A04D, TCGA.A2.A3XT, TCGA.D8.A27F, TCGA.A2.A3XY	HUWE1	low	UNS, BL1, UNS, LAR, NA, M, M
TCGA.S3.AA15, TCGA.A2.A0CM, TCGA.A2.A0SX	NOTCH3	high	MSL, IM, BL2
TCGA.D8.A27M, TCGA.OL.A66P, TCGA.E2.A1B6	IGHV2-70	high	UNS, LAR, IM
TCGA.AO.A128, TCGA.A2.A04T, TCGA.AR.A1AQ	ZNF697	high	IM, BL1, IM
TCGA.C8.A26X, TCGA.A2.A0CM, TCGA.E2.A14X	TLR3	high	LAR, IM, BL1
TCGA.E2.A159, TCGA.EW.A1OV, TCGA.A2.A04T	SYBU	high	BL2, UNS, BL1
TCGA.AO.A128, TCGA.E2.A1LH, TCGA.EW.A1OV	SGIP1	high	IM, MSL, UNS
TCGA.AO.A128, TCGA.EW.A3U0, TCGA.OL.A66P	RALGAPA2	high	IM, UNS, LAR
TCGA.D8.A27M, TCGA.GM.A3XL, TCGA.LL.A441	PPP1R3F	high	UNS, IM, MSL
TCGA.E2.A1LH, TCGA.GM.A2DH, TCGA.A2.A04Q	PARP14	high	MSL, UNS, IM
TCGA.GM.A2DH, TCGA.OL.A66P, TCGA.EW.A1P7	LIMCH1	high	UNS, LAR, UNS
TCGA.E2.A159, TCGA.EW.A1OV, TCGA.GM.A2DI	HIST1H2BC	high	BL2, UNS, NA
TCGA.AO.A128, TCGA.E2.A1LH, TCGA.E2.A573	HEG1	high	IM, MSL, IM
TCGA.S3.AA10, TCGA.BH.A0BG, TCGA.GM.A2DD	FAM120C	high	IM, UNS, LAR
TCGA.AO.A128, TCGA.EW.A3U0, TCGA.AO.A0J6	ETV3L	high	IM, UNS, IM
TCGA.E2.A1LH, TCGA.GM.A2DD, TCGA.A2.A0ST	ARHGAP21	high	MSL, LAR, NA

TCGA: The Cancer Genome Atlas; TNBC: triple negative breast cancer; sd: standard deviation; BL1: basal-like 1; BL2: basal-like 2; IM: immunomodulatory; M: mesenchymal, MSL: mesenchymal stem-like, LAR: luminal androgen receptor; UNS: unstable; NA: not applicable

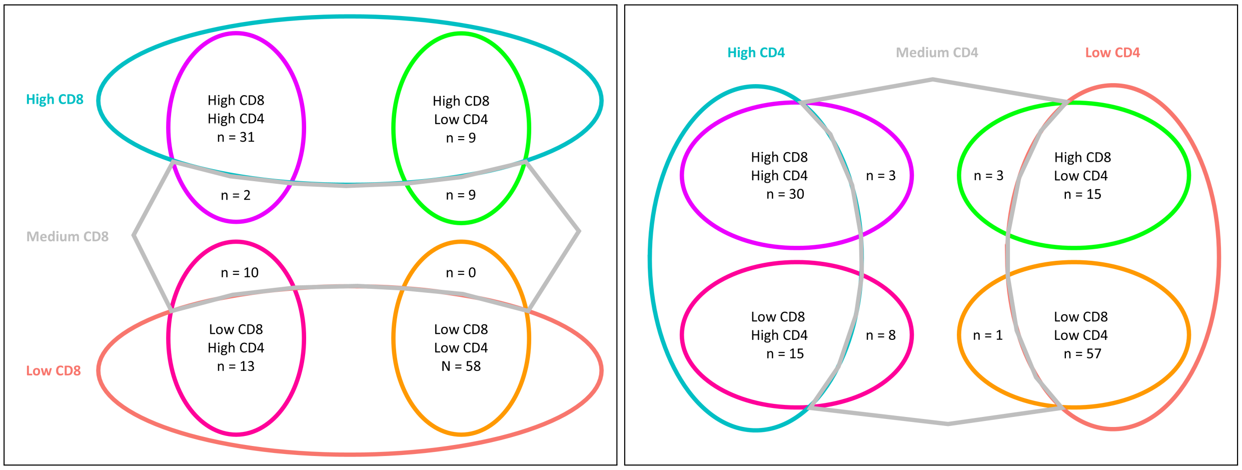

(a) (b)

Figure S3: **Relationship of CD8 T cells and CD4 memory activated T cells in the two group vs. cluster analysis for TCGA. (a)** In the TCGA two group analysis, samples with high CD8 T cells are compared to samples with low CD8 T cells regardless of the CD4 memory activated T cell status. **(b)** In the TCGA two group analysis, samples with high CD4 memory activated T cells are compared to samples with low CD4 memory activated T cells regardless of the CD8 T cell status. **(a)** and **(b)** In the TCGA cluster analysis, only samples with both high CD8 T cells and high CD4 memory activated T cells (magenta) are compared to samples low in both cell types (orange). **(a)** and **(b)** Images generated with Microsoft Powerpoint (Office 365 ProPlus Version 2002) (https://www.microsoft.com).

Table S5: Subtypes of TCGA TNBC Patients

Characteristic	TNBC Patients (n=133)	CD8 T Cells (0.25*sd)				CD8/CD4 T Cells (cluster analysis)			
		High CD8 T Cells (n=40)	Low CD8 T Cells (n=71)	p	fdr	High CD8/ High CD4 (n=33)	Low CD8/ Low CD4 (n=58)	p	fdr
TNBC Subtype				**8.4e-10**				**6.4e-08**	
BL1	17 (13%)	3 (8%)	12 (17%)	0.16	0.63	3 (9%)	7 (12%)	0.74	1
BL2	11 (8%)	2 (5%)	7 (10%)	0.48	1.00	2 (6%)	6 (10%)	0.71	1
IM	20 (15%)	17 (43%)	1 (1%)	**1.2e-07**	**8.5e-07**	14 (42%)	1 (2%)	**8.8e-07**	**6.2e-06**
LAR	17 (13%)	3 (8%)	13 (18%)	0.10	0.50	3 (9%)	11 (19%)	0.24	1
M	25 (19%)	0 (0%)	21 (30%)	**1.1e-05**	**6.7e-05**	0 (0%)	21 (36%)	**1.8e-05**	**1.1e-04**
MSL	11 (8%)	4 (10%)	4 (6%)	0.48	1	3 (9%)	3 (5%)	0.66	1
UNS	23 (17%)	6 (15%)	11 (15%)	1	1	4 (12%)	7 (12%)	1	1
NA	9 (7%)	5 (13%)	2 (3%)			4 (12%)	2 (3%)		

TCGA: The Cancer Genome Atlas; TNBC: triple negative breast cancer; p: pvalue; sd: standard deviation; FDR: false discovery rate; BL1: basal-like 1; BL2: basal-like 2; IM: immunomodulatory; M: mesenchymal, MSL: mesenchymal stem-like, LAR: luminal androgen receptor; UNS: unstable; NA: not applicable; **Bold**: pvalue < 0.05

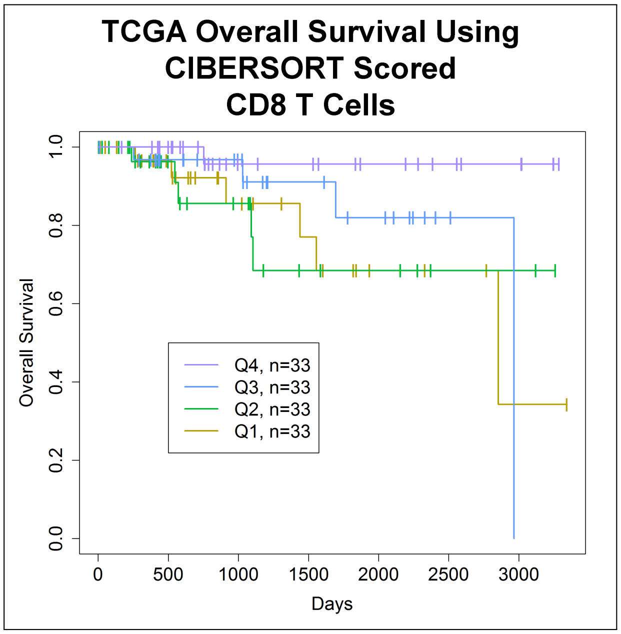

(a)

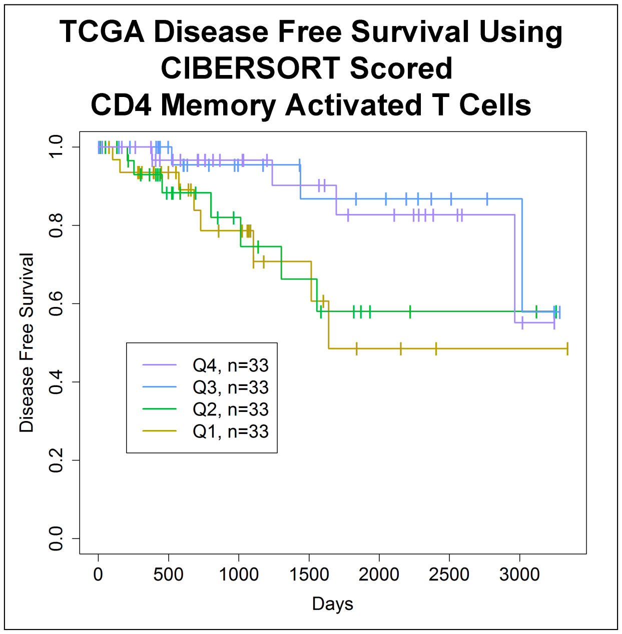

(b)

Figure S4: **Kaplan-Meier curves showing a trend towards improved OS or DFS in TCGA TNBC patients with high quantities of CD8 T cells or CD4 memory activated T cells in their tumor sample.** **(a)** Analysis of TCGA TNBC patients with a higher proportion of CD8 T cells in their tumor (quartiles 3 and 4) show a trend towards improved OS ($p = 0.094$, FDR $= 0.48$, log rank test). **(b)** Analysis of TCGA TNBC patients with a higher proportion of CD4 memory activated T cells in their tumor sample (quartiles 3 and 4) show a trend towards improved DFS ($p = 0.085$, FDR $= 0.42$, log rank test). **(a)** and **(b)** Cut offs of T cell infiltrate were based on quartiles. **(a)** and **(b)** Images generated with R 4.0.2 (https://www.R-project.org) [35].

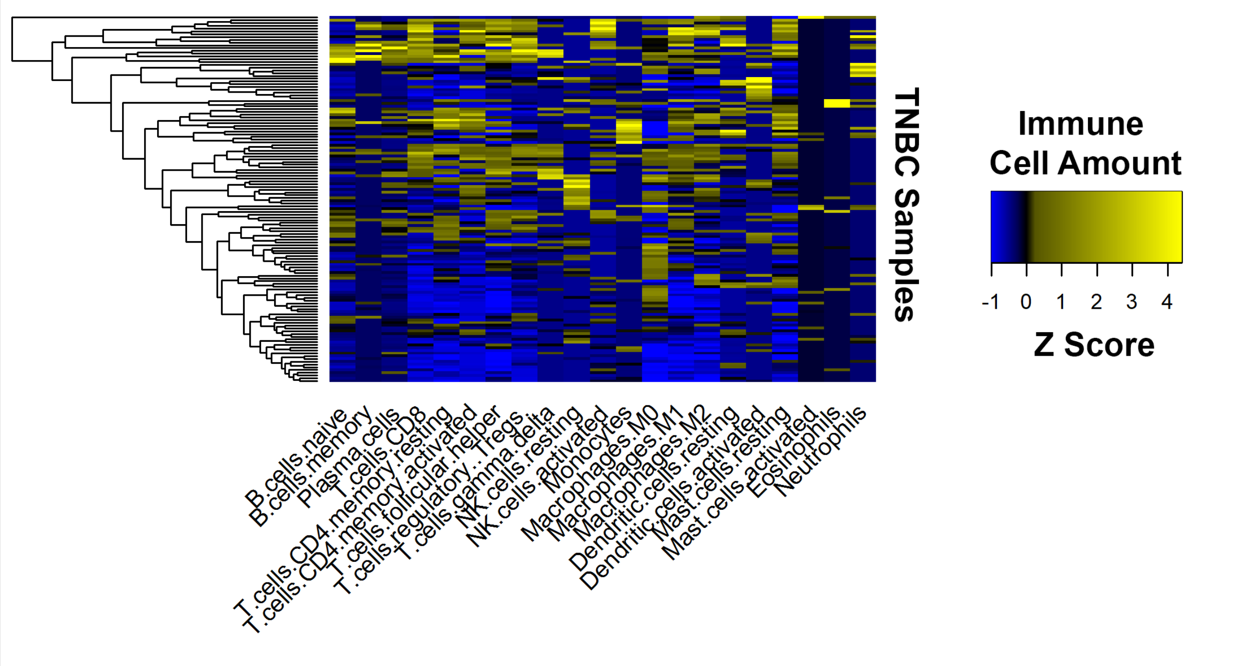

Figure S5: **Hierarchical clustering of all immune cell quantities in TCGA TNBC samples.** TCGA RNA-Seq gene expression data was analyzed with CIBERSORT to quantify the amount of different immune cells in the TNBC samples. Hierarchical clustering of these quantities after normalization is shown in the heatmap. Image generated with R's (4.0.2) (https://www.R-project.org) [35] gplots package (3.0.4) (https://CRAN.R-project.org/package=gplots) [36].

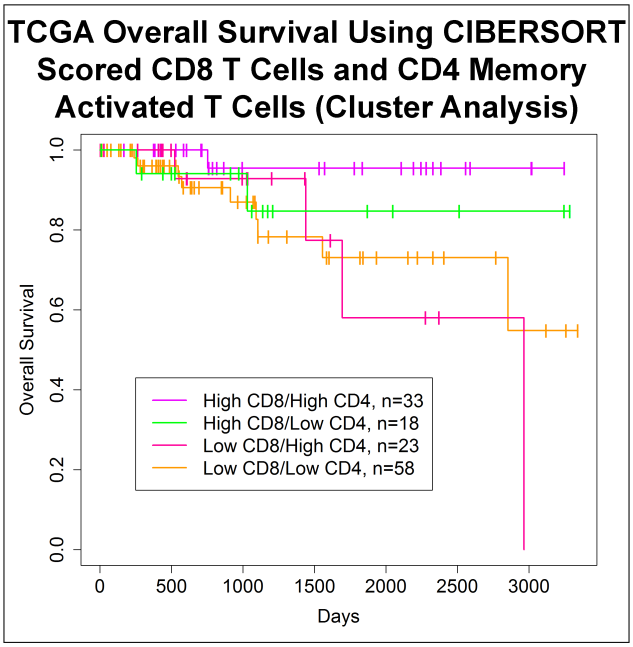

Figure S6: **Kaplan-Meier curve demonstrating improvements in OS in TCGA TNBC patients with high quantities of CD8 T cells and CD4 memory activated T cells in their tumor sample.** TCGA TNBC patients with a higher amount of CD8 T cells and CD4 memory activated T cells in their tumor (magenta) as compared to samples with a lower amount of both these cell types (orange) have a better OS ($p = 0.037$, FDR = 0.11, log rank test) (survival rates high vs. low, 5 year: 95.5% and 73.1%, 10 year: 95.5% and 54.8%). The clusters were identified according to the hierarchical clustering analysis in figure 5. Image generated with R 4.0.2 (https://www.R-project.org) [35].

Table S6: Comparison of TCGA and METABRIC TNBC Patients

Characteristic	TCGA TNBC Patients (n=133)	METABRIC TNBC Patients (n=199)	p
Mean Age at Pathologic Diagnosis	54.7	53.9	0.57*
Pathologic Stage			0.09#
I-II	110 (83%)	129 (65%)	Y
III-IV	20 (15%)	12 (6%)	Y
NA	3 (2%)	58 (29%)	N
Menopause			0.05#
Pre	35 (26%)	82 (41%)	Y
Post	82 (62%)	117 (59%)	Y
NA	16 (12%)	0 (0%)	N
Chemotherapy			**0.0006**#
Yes	103 (77%)	117 (59%)	Y
No/NA	30 (23%)	82 (41%)	Y
Positive Lymph Node			**0.01**#
Yes	49 (37%)	100 (50%)	Y
No	84 (63%)	96 (48%)	Y
NA	0 (0%)	3 (2%)	N

TCGA: The Cancer Genome Atlas; METABRIC: Molecular Taxonomy of Breast Cancer International Consortium; TNBC: triple negative breast cancer; p: pvalue; *: Wilcoxon test; #: Fisher's exact test; Y: Used in Fisher's exact test; N: Not used for Fisher's exact test; NA: missing data; **Bold**: pvalue < 0.05

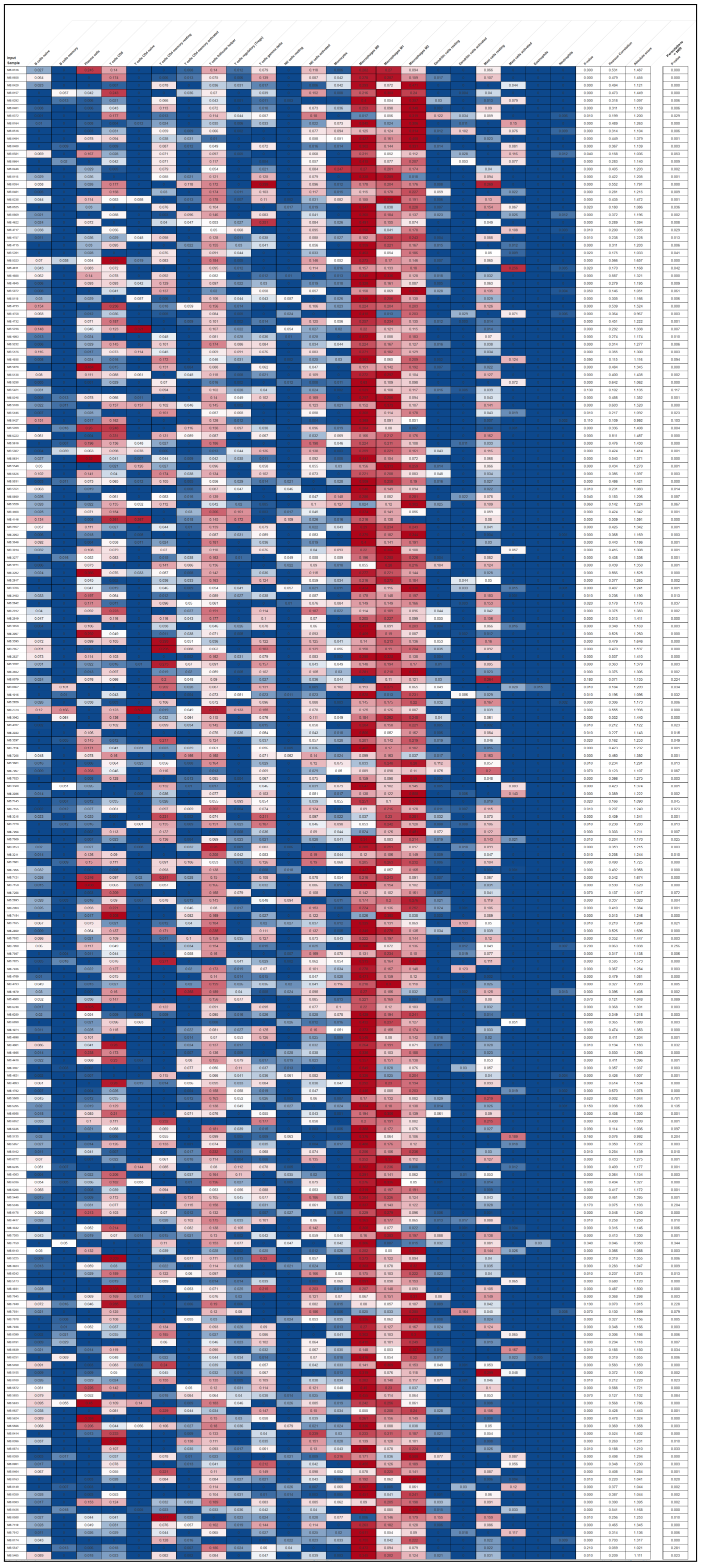

Figure S7: **Quantification of immune cell infiltrate by CIBERSORT analysis of microarray gene expression data from METABRIC TNBC cases.** The heatmap returned by CIBERSORT shows the quantities of 22 different immune cell types among the 196 METABRIC TNBC samples. CIBERSORT assigns a score of arbitrary units that reflects the absolute proportion of each cell type in a mixture. A higher score would indicate a higher proportion of that cell type. The far right column indicates the pvalue when 1000 permutations are used instead of 100. Image generated with CIBERSORT (https://cibersort.stanford.edu) [29].

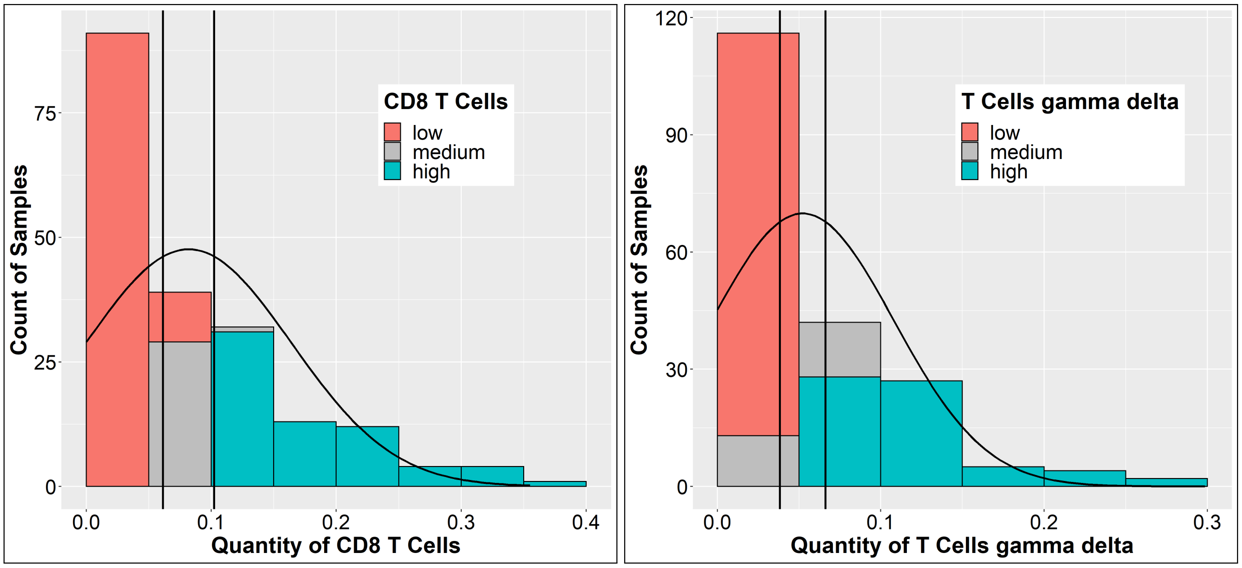

(a) (b)

Figure S8: **Distribution of the proportion of CD8 T cells or gamma delta T cells across the METABRIC TNBC cases.** A histogram of the quantity of **(a)** CD8 T cells or **(b)** gamma delta T cells present across the different METABRIC TNBC samples was plotted using data generated by CIBERSORT. CIBERSORT assigns a score of arbitrary units that reflects the absolute proportion of each cell type in a mixture. A higher score would indicate a higher proportion of that cell type. Vertical lines represent the cut off (+/- 0.25 * standard deviation) used to create the "high" and "low" groups. **(a)** and **(b)** Images generated with R 4.0.2 (https://www.R-project.org) [35].

Table S7: Prognostic Value of Immune Cell Types in METABRIC TNBC Patients (Kaplan-Meier log rank test)

Cell Type	High vs. Low Cut Off	Low Cut Off	High Cut Off	Low Number	High Number	OS pvalue	OS FDR
CD8 T Cells	0.25*sd	0.0613	0.1023	101	65	0.979	0.98
CD8 T Cells	median	0.0581	0.0581	98	98	0.673	0.78
CD8 T Cells	mean	0.0818	0.0818	118	78	0.773	0.94
CD8 T Cells	0.5*sd	0.0407	0.1228	79	50	0.681	0.97
CD8 T Cells	quartiles	0.0000, 0.0175, 0.0581	0.0581, 0.1233, 0.3552	49, 49	49, 49	0.238	0.51
gamma delta T Cells	0.25*sd	0.0382	0.0662	103	66	**0.006**	0.12
gamma delta T Cells	median	0.0362	0.0362	98	98	**0.007**	0.10
gamma delta T Cells	mean	0.0522	0.0522	116	80	**0.011**	0.23
gamma delta T Cells	0.5*sd	0.0242	0.0801	78	48	**0.008**	0.13
gamma delta T Cells	quartiles	0.0000, 0.0035, 0.0362	0.0362, 0.0792, 0.2986	49, 49	49, 49	0.0521	0.16

METABRIC: Molecular Taxonomy of Breast Cancer International Consortium; TNBC: triple negative breast cancer; OS: overall survival; sd: standard deviation; FDR: false discovery rate; **Bold**: pvalue < 0.05

Table S8: Survival Rates of METABRIC TNBC Patients (Kaplan-Meier)

Years	OS High CD8 T Cells (0.25*sd)	OS Low CD8 T Cells (0.25*sd)	OS High T Cells gamma delta (0.25*sd)	OS Low T Cells gamma delta (0.25*sd)
5	66.2%	63.5%	73.9%	56.6%
7	62.9%	53.9%	72.3%	48.0%
10	55.6%	50.3%	67.3%	42.0%
15	43.9%	47.1%	61.3%	37.6%
20	36.6%	47.1%	58.0%	35.4%

METABRIC: Molecular Taxonomy of Breast Cancer International Consortium; TNBC: triple negative breast cancer; OS: overall survival; sd: standard deviation

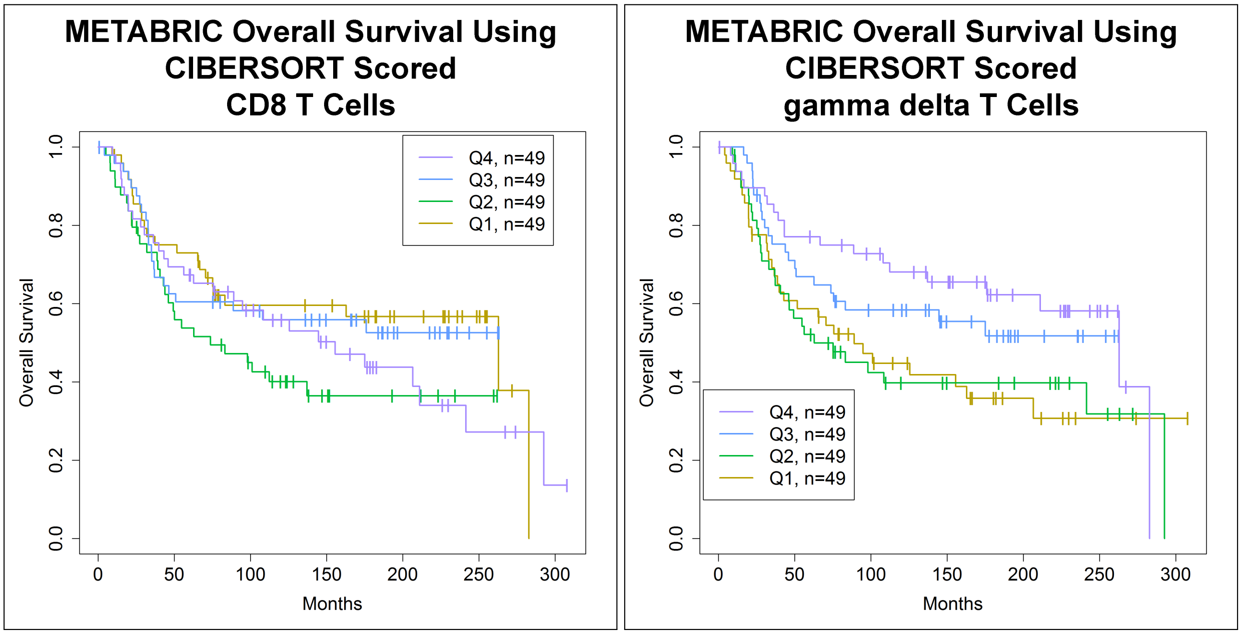

(a) (b)

Figure S9: **Kaplan-Meier curves showing a trend towards improved OS in METABRIC TNBC patients with high quantities of gamma delta T cells in their tumor sample.** **(a)** Analysis of METABRIC TNBC patients with a higher proportion of CD8 T cells in their tumor (quartiles 3 and 4) do not have a better OS (p = 0.238, FDR = 0.51, log rank test). **(b)** Analysis of METABRIC TNBC patients with a higher proportion of gamma delta T cells in their tumor sample (quartiles 3 and 4) show a trend towards improved OS (p = 0.052, FDR = 0.16, log rank test). **(a)** and **(b)** Cut offs of T cell infiltrate were based on quartiles. **(a)** and **(b)** Images generated with R 4.0.2 (https://www.R-project.org) [35].

Table S9: Subtypes of METABRIC TNBC Patients

Characteristic	TNBC Patients (n=199)	CD8 T Cells (0.25*sd) High CD8 T Cells (n=65)	Low CD8 T Cells (n=101)	p	fdr	T Cells gamma delta (0.25*sd) High T Cells gamma delta (n=66)	Low T Cells gamma delta (n=103)	p	fdr
TNBC Subtype				**8.9e-05**				0.16	
BL1	29 (15%)	7 (11%)	15 (15%)	0.49	1	10 (15%)	15 (15%)	0.65	1
BL2	14 (7%)	4 (6%)	8 (8%)	0.77	1	4 (6%)	9 (9%)	0.38	1
IM	45 (23%)	26 (40%)	9 (9%)	**3.3e-06**	**2.3e-05**	17 (26%)	19 (18%)	0.17	1
LAR	18 (9%)	5 (8%)	12 (12%)	0.44	1	8 (12%)	9 (9%)	0.58	1
M	38 (19%)	6 (9%)	31 (31%)	**0.0011**	**0.0065**	6 (9%)	27 (26%)	**0.0089**	0.063
MSL	10 (5%)	4 (6%)	3 (3%)	0.43	1	6 (9%)	4 (4%)	0.34	1
UNS	33 (17%)	10 (15%)	18 (18%)	0.83	1	12 (18%)	15 (15%)	0.42	1
NA	12 (6%)	3 (5%)	5 (5%)			3 (5%)	5 (5%)		

METABRIC: Molecular Taxonomy of Breast Cancer International Consortium; TNBC: triple negative breast cancer; p: pvalue; sd: standard deviation; FDR: false discovery rate; BL1: basal-like 1; BL2: basal-like 2; IM: immunomodulatory; M: mesenchymal, MSL: mesenchymal stem-like, LAR: luminal androgen receptor; UNS: unstable; NA: not applicable; **Bold**: pvalue < 0.05

Table S10: 133 TCGA TNBC Cases

Barcode	% TILs	CD8 T Cells	CD4 Memory Activated T Cells	Subtype	ER IHC	PR IHC	HER2 IHC	HER2 IHC Level	HER2 FISH	Pathologic Stage	Age	Ethnicity	Menopause	Chemotherapy	LN +	OS Status	OS Days	DFS Status	DFS Days
TCGA-A2-A1G6		0.213	0.000	NA	Neg	Neg	Neg	1+		IIIA	50	C	Pre	Y	Pos	0	501	0	501
TCGA-C8-A12V	10-20%	0.578	0.105	IM	Neg	Neg	Neg			IIA	55		Post		Neg	0	385	0	385
TCGA-GI-A2C9	<1%	0.025	0.000	M	Neg	Neg	Neg			IIB	58	AA	Post		Neg	0	3342	0	3342
TCGA-A7-A6VV		0.017	0.000	M	Neg	Neg	Neg			IIA	51	AA		Y	Neg	0	313	0	313
TCGA-D8-A27H	<1%	0.010	0.000	M	Neg	Neg	Neg			IIA	72	C	Post	Y	Neg	0	397	0	397
TCGA-EW-A1OW	10 - 20%	0.014	0.005	M	Neg	Neg	Neg	1+		IIA	58	AA	Post	Y	Neg	0	694	0	694
TCGA-D8-A1XQ	1-10%	0.085	0.042	BL1	Neg	Neg	Neg	1+		IIA	69	C	Post		Neg	0	499	0	499
TCGA-E2-A1LI	1-10%	0.035	0.014	BL1	Neg	Neg	Equiv		Neg	IIB	57	C	Post	Y	Pos	0	3121	0	3121
TCGA-BH-A0BG	20-30%	0.222	0.021	UNS	Neg	Neg	Neg			I	73	C	Post		Neg	0	1871	0	1871

TCGA: The Cancer Genome Atlas; TNBC: triple negative breast cancer; Cluster assignment: high in both T cells, high in CD8 T cells only, high in CD4 memory activated T cells only, low in both T cells; TIL: tumor infiltrating lymphocyte; High quantity of T Cells: > 0.25*standard deviation; Low quantity of T Cells: < 0.25*standard deviation; ER: estrogen receptor; IHC: immunohistochemistry; Pos: Positive; Neg: negative; PR: progesterone receptor; HER2: human epidermal growth factor receptor 2; Equiv: equivocal; Indet: indeterminate; FISH: fluorescence in situ hybridization; C: Caucasion; AA: African American; LN +: Lymph Node Positive; Y: Yes; OS: overall survival; DFS: disease free survival; BL1: basal-like 1; BL2: basal-like 2; IM: immunomodulatory; M: mesenchymal, MSL: mesenchymal stem-like, LAR: luminal androgen receptor; UNS: unstable; NA: not applicable

Table S10: 133 TCGA TNBC Cases

Barcode	% TILs	CD8 T Cells	CD4 Memory Activated T Cells	Subtype	ER IHC	PR IHC	HER2 IHC	HER2 IHC Level	HER2 FISH	Pathologic Stage	Age	Ethnicity	Menopause	Chemotherapy	LN +	OS Status	OS Days	DFS Status	DFS Days
TCGA-AN-A0AR	1-10%	0.026	0.000	LAR	Neg	Neg	Neg	0		IIA	55	C	Pre		Neg	0	10	0	10
TCGA-LL-A441		0.242	0.064	MSL	Neg	Neg	Neg	1+		IA	62	AA	Post	Y	Neg	0	996	0	996
TCGA-A2-A04Q	10-20%	0.321	0.150	IM	Neg	Neg	Equiv	2+	Neg	IA	48	C	Post	Y	Neg	0	2385	0	2385
TCGA-D8-A27F	1-10%	0.007	0.003	M	Neg	Neg	Neg	1+		IIA	40	C	Pre	Y	Neg	0	488	0	488
TCGA-A7-A0DA	<1%	0.029	0.000	M	Neg	Neg	Neg	1+		IIA	62	C	Post	Y	Neg	0	1085	0	1085
TCGA-LL-A5YO	10-20%	0.407	0.188	IM	Neg	Neg	Neg	1+		IA	50	AA	Pre	Y	Neg	0	440	0	440
TCGA-C8-A26Y	<1%	0.017	0.000	LAR	Neg	Neg	Neg	1+		IIA	90		Post		Neg	0	394	0	394
TCGA-D8-A1JL	1-10%	0.141	0.078	UNS	Neg	Neg	Neg	1+		IIA	72	C	Post		Neg	0	611	0	611
TCGA-OL-A5D7		0.188	0.126	IM	Neg	Neg			Neg	IIA	70	AA	Post	Y	Neg	0	1780	0	1780
TCGA-AO-A0J2	10-20%	0.090	0.063	LAR	Neg	Neg	Equiv	2+	Neg	IA	41	C	Pre	Y	Neg	0	997	0	997

TCGA: The Cancer Genome Atlas; TNBC: triple negative breast cancer; Cluster assignment: high in both T cells, high in CD8 T cells only, high in CD4 memory activated T cells only, low in both T cells; TIL: tumor infiltrating lymphocyte; High quantity of T Cells: > 0.25*standard deviation; Low quantity of T Cells: < 0.25*standard deviation; ER: estrogen receptor; IHC: immunohistochemistry; Pos: Positive; Neg: negative; PR: progesterone receptor; HER2: human epidermal growth factor receptor 2; Equiv: equivocal; Indet: indeterminate; FISH: fluorescence in situ hybridization; C: Caucasion; AA: African American; LN +: Lymph Node Positive; Y: Yes; OS: overall survival; DFS: disease free survival; BL1: basal-like 1; BL2: basal-like 2; IM: immunomodulatory; M: mesenchymal, MSL: mesenchymal stem-like, LAR: luminal androgen receptor; UNS: unstable; NA: not applicable

Table S10: 133 TCGA TNBC Cases

Barcode	% TILs	CD8 T Cells	CD4 Memory Activated T Cells	Subtype	ER IHC	PR IHC	HER2 IHC	HER2 IHC Level	HER2 FISH	Pathologic Stage	Age	Ethnicity	Menopause	Chemotherapy	LN +	OS Status	OS Days	DFS Status	DFS Days
TCGA-A2-A0D2	20-30%	0.163	0.088	M	Neg	Neg	Neg	0	Neg	IIA	45	C	Pre	Y	Neg	0	1027	0	1027
TCGA-AR-A0TU	10-20%	0.166	0.158	BL1	Neg	Neg	Neg	1+		IIA	35		Pre	Y	Neg	0	709	0	709
TCGA-AR-A0U1					Neg	Neg	Neg	1+		IIB	36	C	Pre	Y	Pos	0	4052	0	4052
TCGA-AN-A0XU	10-20%	0.104	0.072	UNS	Neg	Neg	Neg			IIA	54	C	Post		Neg	0	10	0	10
TCGA-OL-A5RW		0.000	0.000	UNS	Neg	Neg			Neg	IIA	40	AA	Pre	Y	Pos	0	1106	0	1106
TCGA-EW-A6SB	40-50%	0.238	0.106	IM	Neg	Neg	Neg			II	62	AA			Neg	0	760	0	760
TCGA-AO-A0J4	1-10%	0.083	0.021	BL1	Neg	Neg	Neg			IA	41	AA	Pre	Y	Neg	0	1587	0	1587
TCGA-AQ-A54N	<1%	0.060	0.000	LAR	Neg	Neg	Equiv	2+	Neg	IIA	51	AA	Post		Neg	0	78	0	78
TCGA-AR-A1AY	1-10%	0.016	0.000	M	Neg	Neg	Neg			I	65	C	Post	Y	Neg	0	1026	0	1026
TCGA-AR-A0U4	10-20%	0.030	0.008	IM	Neg	Neg	Neg			IIA	54	C	Pre	Y	Neg	0	3261	0	3261

TCGA: The Cancer Genome Atlas; TNBC: triple negative breast cancer; Cluster assignment: high in both T cells, high in CD8 T cells only, high in CD4 memory activated T cells only, low in both T cells; TIL: tumor infiltrating lymphocyte; High quantity of T Cells: > 0.25*standard deviation; Low quantity of T Cells: < 0.25*standard deviation; ER: estrogen receptor; IHC: immunohistochemistry; Pos: Positive; Neg: negative; PR: progesterone receptor; HER2: human epidermal growth factor receptor 2; Equiv: equivocal; Indet: indeterminate; FISH: fluorescence in situ hybridization; C: Caucasion; AA: African American; LN +: Lymph Node Positive; Y: Yes; OS: overall survival; DFS: disease free survival; BL1: basal-like 1; BL2: basal-like 2; IM: immunomodulatory; M: mesenchymal, MSL: mesenchymal stem-like, LAR: luminal androgen receptor; UNS: unstable; NA: not applicable

Table S10: 133 TCGA TNBC Cases

Barcode	% TILs	CD8 T Cells	CD4 Memory Activated T Cells	Subtype	ER IHC	PR IHC	HER2 IHC	HER2 IHC Level	HER2 FISH	Pathologic Stage	Age	Ethnicity	Menopause	Chemotherapy	LN +	OS Status	OS Days	DFS Status	DFS Days
TCGA-E2-A1L7		0.264	0.053	NA	Neg	Neg	Neg			IIIA	40	C	Pre	Y	Pos	0	1836	0	1836
TCGA-E2-A574	<1%	0.033	0.000	M	Neg	Neg	Equiv	2+	Neg	IIA	44	AA	Pre	Y	Neg	0	1179	0	1179
TCGA-BH-A18G	<1%	0.062	0.026	UNS	Neg	Neg	Neg			IA	81	C	Post		Neg	0	149	0	149
TCGA-A1-A0SO	1-10%	0.016	0.006	UNS	Neg	Neg	Equiv		Neg	IIB	67	C	Post		Pos	0	852	0	852
TCGA-OL-A66I	1-10%	0.559	0.151	IM	Neg	Neg			Neg	IIA	36	AA		Y	Pos	0	714	0	714
TCGA-A7-A4SE	1-10%	0.014	0.000	BL2	Neg	Neg	Neg	1+		IIA	54	AA	Post	Y	Neg	0	644	0	644
TCGA-BH-A0E6		0.162	0.000	UNS	Neg	Neg	Equiv	2+	Neg	IA	69	C	Post	Y	Neg	0	293	0	293
TCGA-OL-A6VO	<1%	0.016	0.000	BL1	Neg	Neg	Neg		Neg	IA	43	AA		Y	Neg	0	858	0	858
TCGA-A2-A0T2	1-10%	0.136	0.016	UNS	Neg	Neg	Neg	0		IV	66	C	Post	Y	Pos	1	255	1	255
TCGA-BH-A0RX		0.371	0.112	NA	Neg	Neg	Neg	1+		IIA	59	C	Post	Y	Neg	0	170	0	170

TCGA: The Cancer Genome Atlas; TNBC: triple negative breast cancer; Cluster assignment: high in both T cells, high in CD8 T cells only, high in CD4 memory activated T cells only, low in both T cells; TIL: tumor infiltrating lymphocyte; High quantity of T Cells: > 0.25*standard deviation; Low quantity of T Cells: < 0.25*standard deviation; ER: estrogen receptor; IHC: immunohistochemistry; Pos: Positive; Neg: negative; PR: progesterone receptor; HER2: human epidermal growth factor receptor 2; Equiv: equivocal; Indet: indeterminate; FISH: fluorescence in situ hybridization; C: Caucasion; AA: African American; LN +: Lymph Node Positive; Y: Yes; OS: overall survival; DFS: disease free survival; BL1: basal-like 1; BL2: basal-like 2; IM: immunomodulatory; M: mesenchymal, MSL: mesenchymal stem-like, LAR: luminal androgen receptor; UNS: unstable; NA: not applicable

Table S10: 133 TCGA TNBC Cases

Barcode	% TILs	CD8 T Cells	CD4 Memory Activated T Cells	Subtype	ER IHC	PR IHC	HER2 IHC	HER2 IHC Level	HER2 FISH	Pathologic Stage	Age	Ethnicity	Menopause	Chemotherapy	LN +	OS Status	OS Days	DFS Status	DFS Days
TCGA-D8-A13Z	1-10%	0.045	0.039	BL2	Neg	Neg	Neg	1+			51	C	Post	Y	Pos	0	635	0	635
TCGA-E2-A159		0.292	0.211	BL2	Neg	Neg			Neg	IIA	50	C	Pre	Y	Neg	0	762	0	762
TCGA-D8-A143	40-50%	0.109	0.041	M	Neg	Neg	Neg	1+		IIA	51	C	Post	Y	Neg	0	431	0	431
TCGA-E2-A14N	<1%	0.061	0.064	UNS	Neg	Neg	Neg			IIB	37	C	Pre	Y	Pos	0	1434	0	1434
TCGA-A2-A3XU	1-10%	0.018	0.000	M	Neg	Neg			Neg	IIB	35	AA	Post	Y	Pos	1	912	1	574
TCGA-A2-A3XT	1-10%	0.002	0.027	NA	Neg	Neg	Neg		Neg	IIB	45	AA	Pre	Y	Pos	0	2770	0	2770
TCGA-AO-A128	20-30%	0.691	0.308	IM	Neg	Neg	Neg	0	Neg	IIA	61	C	Post	Y	Neg	0	3248	0	3248
TCGA-B6-A409	1-10%	0.060	0.007	LAR	Neg	Neg	Equiv	1+	Neg	IIIA	44	AA	Pre	Y	Pos	1	573	1	456
TCGA-D8-A27M	1-10%	0.176	0.114	UNS	Neg	Neg	Neg			IA	59	C	Post	Y	Neg	0	410	0	410
TCGA-E2-A1LK	<1%	0.018	0.000	UNS	Neg	Neg	Equiv	2+	Neg	IIIC	84	AA	Post		Pos	1	266	1	155

TCGA: The Cancer Genome Atlas; TNBC: triple negative breast cancer; Cluster assignment: high in both T cells, high in CD8 T cells only, high in CD4 memory activated T cells only, low in both T cells; TIL: tumor infiltrating lymphocyte; High quantity of T Cells: > 0.25*standard deviation; Low quantity of T Cells: < 0.25*standard deviation; ER: estrogen receptor; IHC: immunohistochemistry; Pos: Positive; Neg: negative; PR: progesterone receptor; HER2: human epidermal growth factor receptor 2; Equiv: equivocal; Indet: indeterminate; FISH: fluorescence in situ hybridization; C: Caucasion; AA: African American; LN +: Lymph Node Positive; Y: Yes; OS: overall survival; DFS: disease free survival; BL1: basal-like 1; BL2: basal-like 2; IM: immunomodulatory; M: mesenchymal, MSL: mesenchymal stem-like, LAR: luminal androgen receptor; UNS: unstable; NA: not applicable

Table S10: 133 TCGA TNBC Cases

Barcode	% TILs	CD8 T Cells	CD4 Memory Activated T Cells	Subtype	ER IHC	PR IHC	HER2 IHC	HER2 IHC Level	HER2 FISH	Pathologic Stage	Age	Ethnicity	Menopause	Chemotherapy	LN +	OS Status	OS Days	DFS Status	DFS Days
TCGA-AN-A0AT	30-40%	0.102	0.024	M	Neg	Neg	Neg			IIA	62	C	Post		Neg	0	10	0	10
TCGA-LL-A740	<1%	0.004	0.020	LAR	Neg	Neg	Equiv	2+	Neg	IA	61	AA	Post	Y	Neg	0	441	0	441
TCGA-A7-A6VW		0.010	0.000	BL2	Neg	Neg	Neg			IIA	48	AA	Post	Y	Neg	0	285	0	285
TCGA-AN-A04D		0.021	0.001	LAR	Neg	Neg	Neg	0		IIB	58	C	Post		Pos	0	52	0	52
TCGA-AO-A0J6	20-30%	0.216	0.018	IM	Neg	Neg	Neg	0		IIA	61	C	Post	Y	Neg	0	1140	0	1140
TCGA-EW-A1PH	10-20%	0.145	0.065	M	Neg	Neg	Neg			IIA	52	C	Post	Y	Pos	0	607	0	607
TCGA-BH-A0B9	30-40%	0.313	0.097	IM	Neg	Neg	Neg	0		IA	44	C	Pre	Y	Neg	0	1572	0	1572
TCGA-A2-A3XX	1-10%	0.018	0.054	BL1	Neg	Neg	Neg	1+	Neg	IIA	49	AA	Post	Y	Neg	1	1439	1	1439
TCGA-AR-A256		0.018	0.000	M	Neg	Neg	Neg	1+		IIA	45	C	Pre	Y	Neg	1	2854	1	1640
TCGA-D8-A1JF	1-10%	0.075	0.004	LAR	Neg	Neg	Neg	1+		IIIA	79	C	Post		Pos	0	366	0	366

TCGA: The Cancer Genome Atlas; TNBC: triple negative breast cancer; Cluster assignment: high in both T cells, high in CD8 T cells only, high in CD4 memory activated T cells only, low in both T cells; TIL: tumor infiltrating lymphocyte; High quantity of T Cells: > 0.25*standard deviation; Low quantity of T Cells: < 0.25*standard deviation; ER: estrogen receptor; IHC: immunohistochemistry; Pos: Positive; Neg: negative; PR: progesterone receptor; HER2: human epidermal growth factor receptor 2; Equiv: equivocal; Indet: indeterminate; FISH: fluorescence in situ hybridization; C: Caucasion; AA: African American; LN +: Lymph Node Positive; Y: Yes; OS: overall survival; DFS: disease free survival; BL1: basal-like 1; BL2: basal-like 2; IM: immunomodulatory; M: mesenchymal, MSL: mesenchymal stem-like, LAR: luminal androgen receptor; UNS: unstable; NA: not applicable

Table S10: 133 TCGA TNBC Cases

Barcode	% TILs	CD8 T Cells	CD4 Memory Activated T Cells	Subtype	ER IHC	PR IHC	HER2 IHC	HER2 IHC Level	HER2 FISH	Pathologic Stage	Age	Ethnicity	Menopause	Chemotherapy	LN +	OS Status	OS Days	DFS Status	DFS Days
TCGA-GM-A2DB	10-20%	0.094	0.000	LAR	Neg	Neg	Neg	1+	Neg	IIA	62	C	Post	Y	Neg	0	2406	0	2406
TCGA-BH-A0AV		0.010	0.008	M	Neg	Neg			Neg	I	52	AA		Y	Neg	0	1820	0	1820
TCGA-B6-A402	1-10%	0.014	0.000	M	Neg	Neg	Neg	1+		I	47	AA		Y	Neg	0	2330	1	1516
TCGA-E2-A14X	20-30%	0.180	0.033	BL1	Neg	Neg	Neg	1+		IIIA	55	AA	Post	Y	Pos	0	972	0	972
TCGA-A2-A0ST		0.480	0.052	NA	Neg	Neg			Neg	IIA	62	C	Post	Y	Pos	0	3017	1	3017
TCGA-BH-A1F6	<1%	0.156	0.079	BL1	Neg	Neg	Neg				51	C			Pos	1	2965	1	2965
TCGA-E2-A1LH	1-10%	0.299	0.035	MSL	Neg	Neg	Neg			I	59	C	Post	Y	Neg	0	3247	0	3247
TCGA-S3-AA15		0.287	0.018	MSL	Neg	Neg	Neg			IIB	51	AA	Post	Y	Pos	0	525	0	525
TCGA-A8-A07O	10-20%	0.042	0.020	BL1	Neg	Neg	Neg	1+	Neg	IIA	51		Post		Neg	0	304	0	304
TCGA-EW-A1P7		0.362	0.000	UNS	Neg	Neg	Equiv	2+	Neg	IIA	59	C	Post	Y	Neg	0	915	1	729

TCGA: The Cancer Genome Atlas; TNBC: triple negative breast cancer; Cluster assignment: high in both T cells, high in CD8 T cells only, high in CD4 memory activated T cells only, low in both T cells; TIL: tumor infiltrating lymphocyte; High quantity of T Cells: > 0.25*standard deviation; Low quantity of T Cells: < 0.25*standard deviation; ER: estrogen receptor; IHC: immunohistochemistry; Pos: Positive; Neg: negative; PR: progesterone receptor; HER2: human epidermal growth factor receptor 2; Equiv: equivocal; Indet: indeterminate; FISH: fluorescence in situ hybridization; C: Caucasion; AA: African American; LN +: Lymph Node Positive; Y: Yes; OS: overall survival; DFS: disease free survival; BL1: basal-like 1; BL2: basal-like 2; IM: immunomodulatory; M: mesenchymal, MSL: mesenchymal stem-like, LAR: luminal androgen receptor; UNS: unstable; NA: not applicable

Table S10: 133 TCGA TNBC Cases

Barcode	% TILs	CD8 T Cells	CD4 Memory Activated T Cells	Subtype	ER IHC	PR IHC	HER2 IHC	HER2 IHC Level	HER2 FISH	Pathologic Stage	Age	Ethnicity	Menopause	Chemotherapy	LN +	OS Status	OS Days	DFS Status	DFS Days
TCGA-A1-A0SP	20-30%	0.044	0.004	M	Neg	Neg	Neg	0		IIA	40				Neg	0	584	0	584
TCGA-A2-A3XS	10-20%	0.133	0.000	MSL	Neg	Neg			Neg	IIIA	62	AA	Post	Y	Pos	1	1032	1	682
TCGA-A8-A08R	1-10%	0.081	0.070	BL1	Neg	Neg	Neg	1+	Neg	IIB	52		Post		Pos	0	30	0	30
TCGA-C8-A27B	20-30%	0.131	0.077	UNS	Neg	Neg	Neg			IIB	48			Y	Neg	0	439	0	439
TCGA-D8-A1XK	10-20%	0.050	0.068	BL1	Neg	Neg	Neg			IIB	55	C	Post	Y	Pos	0	441	0	441
TCGA-AO-A12F	10-20%	0.025	0.000	UNS	Neg	Neg	Neg	1+		IIA	36	C	Pre	Y	Neg	0	1842	0	1842
TCGA-AR-A1AQ		0.265	0.177	IM	Neg	Neg	Equiv	2+	Neg	IIA	49	C	Post	Y	Neg	0	3021	0	3021
TCGA-C8-A1HJ	10-20%	0.080	0.053	MSL	Neg	Neg	Neg			IIA	53				Neg	0	5	0	5
TCGA-AC-A6IW	20-30%	0.159	0.062	IM	Neg	Neg	Neg			IIA	73	AA	Post		Neg	0	413	0	413
TCGA-BH-A1EW		0.143	0.091	NA	Neg	Neg	Neg			IIA	38	C	Pre		Pos	1	1694	1	1694

TCGA: The Cancer Genome Atlas; TNBC: triple negative breast cancer; Cluster assignment: high in both T cells, high in CD8 T cells only, high in CD4 memory activated T cells only, low in both T cells; TIL: tumor infiltrating lymphocyte; High quantity of T Cells: > 0.25*standard deviation; Low quantity of T Cells: < 0.25*standard deviation; ER: estrogen receptor; IHC: immunohistochemistry; Pos: Positive; Neg: negative; PR: progesterone receptor; HER2: human epidermal growth factor receptor 2; Equiv: equivocal; Indet: indeterminate; FISH: fluorescence in situ hybridization; C: Caucasion; AA: African American; LN +: Lymph Node Positive; Y: Yes; OS: overall survival; DFS: disease free survival; BL1: basal-like 1; BL2: basal-like 2; IM: immunomodulatory; M: mesenchymal, MSL: mesenchymal stem-like, LAR: luminal androgen receptor; UNS: unstable; NA: not applicable

Table S10: 133 TCGA TNBC Cases

Barcode	% TILs	CD8 T Cells	CD4 Memory Activated T Cells	Subtype	ER IHC	PR IHC	HER2 IHC	HER2 IHC Level	HER2 FISH	Pathologic Stage	Age	Ethnicity	Menopause	Chemotherapy	LN +	OS Status	OS Days	DFS Status	DFS Days
TCGA-E2-A1LL		0.004	0.002	BL2	Neg	Neg	Neg	1+		IIIA	73	AA	Post	Y	Pos	0	1309	1	1303
TCGA-E2-A14R	10-20%	0.108	0.027	IM	Neg	Neg	Neg	1+		IIA	62	C	Post	Y	Neg	0	1174	0	1174
TCGA-A8-A07C	30-40%	0.090	0.097	BL1	Neg	Neg	Neg	1+	Neg	IIA	57		Post	Y	Neg	0	1034	0	1034
TCGA-EW-A1P1		0.157	0.025	MSL	Neg	Neg	Equiv	2+	Neg	IIIC	68	C	Post	Y	Pos	0	1210	1	1014
TCGA-AQ-A04J	<1%	0.210	0.118	MSL	Neg	Neg	Neg	0		IIA	45	C	Pre	Y	Neg	0	819	0	819
TCGA-AR-A1AR	<1%	0.029	0.060	UNS	Neg	Neg	Neg			IIIA	50	C	Post	Y	Pos	1	524	1	524
TCGA-AO-A1KR		0.134	0.039	LAR	Neg	Neg	Neg	1+	Neg	IIA	51	C	Pre	Y	Neg	0	2513	0	2513
TCGA-GM-A3XL	20-30%	0.177	0.133	IM	Neg	Neg			Neg	IIA	49	AA	Post	Y	Neg	0	2108	0	2108
TCGA-BH-A0B3		0.127	0.098	BL2	Neg	Neg	Neg	1+		IIB	53	C	Post	Y	Pos	0	1203	0	1203
TCGA-BH-A0E0	10-20%	0.025	0.002	LAR	Neg	Neg	Neg	1+		IIIC	38	C	Pre	Y	Pos	0	134	0	134

TCGA: The Cancer Genome Atlas; TNBC: triple negative breast cancer; Cluster assignment: high in both T cells, high in CD8 T cells only, high in CD4 memory activated T cells only, low in both T cells; TIL: tumor infiltrating lymphocyte; High quantity of T Cells: > 0.25*standard deviation; Low quantity of T Cells: < 0.25*standard deviation; ER: estrogen receptor; IHC: immunohistochemistry; Pos: Positive; Neg: negative; PR: progesterone receptor; HER2: human epidermal growth factor receptor 2; Equiv: equivocal; Indet: indeterminate; FISH: fluorescence in situ hybridization; C: Caucasion; AA: African American; LN +: Lymph Node Positive; Y: Yes; OS: overall survival; DFS: disease free survival; BL1: basal-like 1; BL2: basal-like 2; IM: immunomodulatory; M: mesenchymal, MSL: mesenchymal stem-like, LAR: luminal androgen receptor; UNS: unstable; NA: not applicable

Table S10: 133 TCGA TNBC Cases

Barcode	% TILs	CD8 T Cells	CD4 Memory Activated T Cells	Subtype	ER IHC	PR IHC	HER2 IHC	HER2 IHC Level	HER2 FISH	Pathologic Stage	Age	Ethnicity	Menopause	Chemotherapy	LN +	OS Status	OS Days	DFS Status	DFS Days
TCGA-E2-A573	>70%	0.189	0.000	IM	Neg	Neg	Equiv	2+	Neg	IA	48	AA		Y	Neg	0	1062	0	1062
TCGA-GM-A2DD	10-20%	0.197	0.120	LAR	Neg	Neg	Equiv	2+	Neg	I	53	C	Post	Y	Neg	0	2282	0	2282
TCGA-A7-A0CE	1-10%	0.050	0.000	M	Neg	Neg	Equiv	2+	Neg	IIA	57	C		Y	Neg	0	1074	0	1074
TCGA-A2-A0YE	1-10%	0.017	0.000	M	Neg	Neg	Neg	0		IIB	48	C	Post	Y	Pos	0	554	0	554
TCGA-EW-A1OV	50-60%	0.537	0.073	UNS	Neg	Neg	Neg		Neg	IIB	56	C	Post	Y	Pos	0	789	0	789
TCGA-D8-A142		0.081	0.014	M	Neg	Neg	Equiv	2+	Neg	IIB	74	C	Post	Y	Neg	0	425	0	425
TCGA-A2-A3XY	20-30%	0.033	0.014	M	Neg	Neg	Neg		Neg	IIB	49	AA	Post	Y	Pos	1	1093	1	802
TCGA-GM-A2DF	1-10%	0.067	0.000	MSL	Neg	Neg	Neg	1+	Neg	IIA	53	AA	Post	Y	Pos	0	2155	0	2155
TCGA-A2-A0SX	30-40%	0.220	0.083	BL2	Neg	Neg	Neg	1+		IA	48	C	Pre	Y	Neg	0	1534	1	1239
TCGA-A7-A26I	<1%	0.000	0.000	MSL	Neg	Neg	Equiv	2+	Neg	IIA	65	C	Post	Y	Neg	0	661	0	661

TCGA: The Cancer Genome Atlas; TNBC: triple negative breast cancer; Cluster assignment: high in both T cells, high in CD8 T cells only, high in CD4 memory activated T cells only, low in both T cells; TIL: tumor infiltrating lymphocyte; High quantity of T Cells: > 0.25*standard deviation; Low quantity of T Cells: < 0.25*standard deviation; ER: estrogen receptor; IHC: immunohistochemistry; Pos: Positive; Neg: negative; PR: progesterone receptor; HER2: human epidermal growth factor receptor 2; Equiv: equivocal; Indet: indeterminate; FISH: fluorescence in situ hybridization; C: Caucasion; AA: African American; LN +: Lymph Node Positive; Y: Yes; OS: overall survival; DFS: disease free survival; BL1: basal-like 1; BL2: basal-like 2; IM: immunomodulatory; M: mesenchymal, MSL: mesenchymal stem-like, LAR: luminal androgen receptor; UNS: unstable; NA: not applicable

Table S10: 133 TCGA TNBC Cases

Barcode	% TILs	CD8 T Cells	CD4 Memory Activated T Cells	Subtype	ER IHC	PR IHC	HER2 IHC	HER2 IHC Level	HER2 FISH	Pathologic Stage	Age	Ethnicity	Menopause	Chemotherapy	LN +	OS Status	OS Days	DFS Status	DFS Days
TCGA-AN-A0AL	20-30%	0.037	0.032	MSL	Neg	Neg	Neg	0		IIIB	41	C	Pre		Neg	0	227	0	227
TCGA-E2-A1B6		0.421	0.271	IM	Neg	Neg	Equiv	2+	Neg	IIA	44	C	Pre	Y	Neg	0	867	0	867
TCGA-OL-A66P	20-30%	0.506	0.065	LAR	Neg	Neg			Neg	IIA	75	AA		Y	Neg	0	428	0	428
TCGA-EW-A1P8	10-20%	0.075	0.012	BL2	Neg	Neg	Neg			IIIC	58	C	Post	Y	Pos	1	239	1	207
TCGA-EW-A3U0	<1%	0.417	0.164	UNS	Neg	Neg	Neg			IIIA	61	AA	Post	Y	Pos	0	532	0	532
TCGA-AO-A129	10-20%	0.193	0.047	IM	Neg	Neg	Neg	0	Neg	IIB	29	C	Pre	Y	Pos	0	3286	0	3286
TCGA-A2-A04P	1-10%	0.076	0.000	NA	Neg	Neg			Neg	IIIC	36	AA	Pre	Y	Pos	1	548	1	102
TCGA-A2-A0CM	40-50%	0.212	0.100	IM	Neg	Neg	Neg	0		IIA	40	AA	Pre	Y	Neg	1	754	1	382
TCGA-A2-A0YM	10-20%	0.034	0.009	BL1	Neg	Neg			Neg	IIA	67	C	Post		Neg	0	965	0	965
TCGA-D8-A147	10-20%	0.045	0.000	BL1	Neg	Neg	Neg				45	C		Y	Neg	0	584	0	584

TCGA: The Cancer Genome Atlas; TNBC: triple negative breast cancer; Cluster assignment: high in both T cells, high in CD8 T cells only, high in CD4 memory activated T cells only, low in both T cells; TIL: tumor infiltrating lymphocyte; High quantity of T Cells: > 0.25*standard deviation; Low quantity of T Cells: < 0.25*standard deviation; ER: estrogen receptor; IHC: immunohistochemistry; Pos: Positive; Neg: negative; PR: progesterone receptor; HER2: human epidermal growth factor receptor 2; Equiv: equivocal; Indet: indeterminate; FISH: fluorescence in situ hybridization; C: Caucasion; AA: African American; LN +: Lymph Node Positive; Y: Yes; OS: overall survival; DFS: disease free survival; BL1: basal-like 1; BL2: basal-like 2; IM: immunomodulatory; M: mesenchymal, MSL: mesenchymal stem-like, LAR: luminal androgen receptor; UNS: unstable; NA: not applicable

Table S10: 133 TCGA TNBC Cases

Barcode	% TILs	CD8 T Cells	CD4 Memory Activated T Cells	Subtype	ER IHC	PR IHC	HER2 IHC	HER2 IHC Level	HER2 FISH	Pathologic Stage	Age	Ethnicity	Menopause	Chemotherapy	LN +	OS Status	OS Days	DFS Status	DFS Days
TCGA-E2-A158	20-30%	0.038	0.000	M	Neg	Neg	Neg	1+		IIA	43	C	Pre	Y	Pos	0	450	0	450
TCGA-E2-A150	<1%	0.006	0.007	BL2	Neg	Neg	Neg			IIA	48	C	Post	Y	Neg	0	1935	0	1935
TCGA-AC-A2BK	1-10%	0.085	0.004	M	Neg	Neg	Neg			IIIA	78	AA	Post	Y	Pos	0	2222	0	2222
TCGA-D8-A1JG		0.098	0.083	LAR	Neg	Neg	Equiv	2+	Neg	IIA	62	C	Post	Y	Neg	0	1612	0	1612
TCGA-AN-A0G0	10-20%	0.018	0.020	UNS	Neg	Neg	Neg	1+		IIA	56	C	Post		Neg	0	16	0	16
TCGA-S3-AA10	40-50%	0.273	0.085	IM	Neg	Neg	Neg	1+		IIA	65	AA	Post	Y	Neg	0	586	0	586
TCGA-C8-A131	20-30%	0.067	0.019	LAR	Neg	Neg	Neg	1+		IIIA	82		Post	Y	Pos	0	411	0	411
TCGA-E2-A1LS	1-10%	0.026	0.000	UNS	Neg	Neg	Neg	1+		IA	46	C	Pre	Y	Neg	0	1604	0	1604
TCGA-A2-A0T0	1-10%	0.029	0.002	M	Neg	Neg	Neg	0		IIB	59	C	Post	Y	Pos	0	533	0	533
TCGA-GM-A2DI		0.590	0.224	NA	Neg	Neg			Neg	I	52	C	Pre	Y	Neg	0	2590	0	2590

TCGA: The Cancer Genome Atlas; TNBC: triple negative breast cancer; Cluster assignment: high in both T cells, high in CD8 T cells only, high in CD4 memory activated T cells only, low in both T cells; TIL: tumor infiltrating lymphocyte; High quantity of T Cells: > 0.25*standard deviation; Low quantity of T Cells: < 0.25*standard deviation; ER: estrogen receptor; IHC: immunohistochemistry; Pos: Positive; Neg: negative; PR: progesterone receptor; HER2: human epidermal growth factor receptor 2; Equiv: equivocal; Indet: indeterminate; FISH: fluorescence in situ hybridization; C: Caucasion; AA: African American; LN +: Lymph Node Positive; Y: Yes; OS: overall survival; DFS: disease free survival; BL1: basal-like 1; BL2: basal-like 2; IM: immunomodulatory; M: mesenchymal, MSL: mesenchymal stem-like, LAR: luminal androgen receptor; UNS: unstable; NA: not applicable

Table S10: 133 TCGA TNBC Cases

Barcode	% TILs	CD8 T Cells	CD4 Memory Activated T Cells	Subtype	ER IHC	PR IHC	HER2 IHC	HER2 IHC Level	HER2 FISH	Pathologic Stage	Age	Ethnicity	Menopause	Chemotherapy	LN +	OS Status	OS Days	DFS Status	DFS Days
TCGA-A7-A6VY		0.065	0.106	BL2	Neg	Neg	Neg			IIB	48	AA	Post	Y	Pos	0	266	0	266
TCGA-BH-A0BL	30-40%	0.052	0.074	UNS	Neg	Neg	Neg			I	35	C	Pre	Y	Neg	0	2278	0	2278
TCGA-A2-A04T	20-30%	0.191	0.093	BL1	Neg	Neg	Equiv	2+	Neg	IIA	62	C	Post	Y	Neg	0	2246	0	2246
TCGA-C8-A26X	20-30%	0.183	0.093	LAR	Neg	Neg	Neg	1+		IIA	58		Post	Y	Pos	0	376	0	376
TCGA-AR-A0TS	<1%	0.247	0.117	BL1	Neg	Neg	Neg	1+		IIB	46	C	Pre	Y	Pos	0	2558	0	2558
TCGA-BH-A0BW		0.084	0.071	UNS	Neg	Neg			Neg	I	71	AA	Post	Y	Neg	0	2371	0	2371
TCGA-B6-A400	10-20%	0.032	0.003	M	Neg	Neg	Neg	1+		IIIA	43	AA			Pos	0	215	0	215
TCGA-EW-A1PB	1-10%	0.220	0.069	IM	Neg	Neg	Neg			IIIA	70	AA	Post		Pos	0	608	0	608
TCGA-GM-A2DH	20-30%	0.251	0.079	UNS	Neg	Neg	Neg	1+	Neg	I	58	C	Post	Y	Neg	0	2193	0	2193
TCGA-E2-A1AZ	<1%	0.136	0.124	MSL	Neg	Neg	Indet		Neg	IIB	63	C	Post	Y	Pos	0	2329	0	2329

TCGA: The Cancer Genome Atlas; TNBC: triple negative breast cancer; Cluster assignment: high in both T cells, high in CD8 T cells only, high in CD4 memory activated T cells only, low in both T cells; TIL: tumor infiltrating lymphocyte; High quantity of T Cells: > 0.25*standard deviation; Low quantity of T Cells: < 0.25*standard deviation; ER: estrogen receptor; IHC: immunohistochemistry; Pos: Positive; Neg: negative; PR: progesterone receptor; HER2: human epidermal growth factor receptor 2; Equiv: equivocal; Indet: indeterminate; FISH: fluorescence in situ hybridization; C: Caucasion; AA: African American; LN +: Lymph Node Positive; Y: Yes; OS: overall survival; DFS: disease free survival; BL1: basal-like 1; BL2: basal-like 2; IM: immunomodulatory; M: mesenchymal, MSL: mesenchymal stem-like, LAR: luminal androgen receptor; UNS: unstable; NA: not applicable

Table S10: 133 TCGA TNBC Cases

Barcode	% TILs	CD8 T Cells	CD4 Memory Activated T Cells	Subtype	ER IHC	PR IHC	HER2 IHC	HER2 IHC Level	HER2 FISH	Pathologic Stage	Age	Ethnicity	Menopause	Chemotherapy	LN +	OS Status	OS Days	DFS Status	DFS Days
TCGA-OL-A5D6	<1%	0.039	0.000	LAR	Neg	Neg			Neg	IIA	71	AA	Post		Neg	1	1104	1	1104
TCGA-A2-A0D0	10-20%	0.124	0.038	UNS	Neg	Neg	Neg	0	Neg	IIA	60	AA	Post	Y	Neg	0	2048	0	2048
TCGA-BH-A18V	10-20%	0.006	0.001	BL1	Neg	Neg	Neg			IIB	48	C			Pos	1	1556	1	1556
TCGA-A7-A4SD		0.154	0.019	BL2	Neg	Neg	Equiv	2+	Neg	IIA	52	AA	Post	Y	Neg	0	441	0	441

TCGA: The Cancer Genome Atlas; TNBC: triple negative breast cancer; Cluster assignment: high in both T cells, high in CD8 T cells only, high in CD4 memory activated T cells only, low in both T cells; TIL: tumor infiltrating lymphocyte; High quantity of T Cells: > 0.25*standard deviation; Low quantity of T Cells: < 0.25*standard deviation; ER: estrogen receptor; IHC: immunohistochemistry; Pos: Positive; Neg: negative; PR: progesterone receptor; HER2: human epidermal growth factor receptor 2; Equiv: equivocal; Indet: indeterminate; FISH: fluorescence in situ hybridization; C: Caucasion; AA: African American; LN +: Lymph Node Positive; Y: Yes; OS: overall survival; DFS: disease free survival; BL1: basal-like 1; BL2: basal-like 2; IM: immunomodulatory; M: mesenchymal, MSL: mesenchymal stem-like, LAR: luminal androgen receptor; UNS: unstable; NA: not applicable

Table S11: 199 METABRIC TNBC Cases

Patient ID	CD8 T Cells	T Cells gamma delta	Sub-type	ER IHC	ER Status	PR Status	HER2 Status	HER2 SNP6	Tumor Stage	Age At Diag-nosis	In-ferred Meno-pause	Che-mo-ther-apy	LN+	OS Sta-tus	OS Months
MB-0062	0.000	0.131	BL1	Neg	Neg	Neg	Neg	Neutral	1	52.14	Post	Y	Neg	0	153.97
MB-0079	0.066	0.027	LAR	Neg	Neg	Neg	Neg	Neutral	2	50.42	Post	Y	Pos	1	28.5
MB-0100	0.024	0.109	UNS	Neg	Neg	Neg	Neg	Neutral	2	68.68	Post	Y	Neg	1	8.07
MB-0115	0.000	0.125	UNS	Neg	Neg	Neg	Neg	Neutral	2	39.84	Pre	Y	Neg	1	66.73
MB-0149	0.000	0.000	M	Neg	Neg	Neg	Neg	Loss	2	77.13	Post	N	Neg	1	51.7
MB-0157	0.243	0.03	UNS	Neg	Neg	Neg	Neg	Neutral	2	71.5	Post	N	Neg	0	114.77
MB-0158				Neg	Neg	Neg	Neg	Neutral	2	50.43	Post	Y		0	192.3
MB-0163	0.028	0.021	M	Neg	Neg	Neg	Neg	Neutral	NA	84.73	Post	N	Pos	1	98.1
MB-0164	0.004	0.033	BL2	Neg	Neg	Neg	Neg	Neutral	2	37.87	Pre	Y	Neg	0	10.83
MB-0174	0.000	0.001	UNS	Neg	Neg	Neg	Neg	Neutral	2	59.18	Post	Y	Neg	0	78.77
MB-0191	0.000	0.102	UNS	Neg	Neg	Neg	Neg	Neutral	2	81.02	Post	N	Neg	1	15.3
MB-0238	0.053	0.110	IM	Neg	Neg	Neg	Neg	Neutral	2	52.19	Post	Y	Pos	0	193.17
MB-0269	0.037	0.000	UNS	Neg	Neg	Neg	Neg	Neutral	2	45.39	Pre	Y	Pos	0	22.23
MB-0292	0.021	0.011	LAR	Neg	Neg	Neg	Neg	Neutral	2	80.34	Post	N	Pos	1	49.43
MB-0303	0.124	0.083	IM	Neg	Neg	Neg	Neg	Neutral	1	47.71	Pre	N	Neg	0	60.13
MB-0316	0.140	0.079	IM	Neg	Neg	Neg	Neg	Neutral	2	50.82	Post	N	Neg	0	182.9
MB-0350	0.053	0.010	M	Neg	Neg	Neg	Neg	Neutral	2	49.05	Pre	N	Neg	1	46.07
MB-0354	0.177	0.299	IM	Neg	Neg	Neg	Neg	Neutral	2	73.01	Post	N	Pos	1	11.6
MB-0372	0.177	0.057	IM	Neg	Neg	Neg	Neg	Neutral	1	39.86	Pre	Y	Neg	1	62.77
MB-0396	0.355	0.035	UNS	Neg	Neg	Neg	Neg	Neutral	2	56.45	Post	Y	Neg	0	60.67
MB-0399	0.035	0.086	LAR	Neg	Neg	Neg	Neg	Neutral	3	68.47	Post	Y	Pos	1	137.1
MB-0400	0.009	0.072	M	Neg	Neg	Neg	Neg	Neutral	1	55.28	Post	Y	Neg	1	22.47
MB-0401	0.043	0.018	M	Neg	Neg	Neg	Neg	Neutral	1	48.58	Pre	Y	Neg	1	26.27
MB-0414	0.233	0.040	IM	Neg	Neg	Neg	Neg	Loss	2	72.16	Post	N	Neg	0	76.63

METABRIC: Molecular Taxonomy of Breast Cancer International Consortium; TNBC: triple negative breast cancer; High quantity of T Cells: > 0.25*standard deviation; Low quantity of T Cells: < 0.25*standard deviation; ER: estrogen receptor; IHC: immunohistochemistry; Pos: Positive; Neg: negative; PR: progesterone receptor; HER2: human epidermal growth factor receptor 2; LN +: Lymph Node Positive; Y: Yes; N: No; OS: overall survival; DFS: disease free survival; BL1: basal-like 1; BL2: basal-like 2; IM: immunomodulatory; M: mesenchymal, MSL: mesenchymal stem-like, LAR: luminal androgen receptor; UNS: unstable; NA: not applicable

Table S11: 199 METABRIC TNBC Cases

Patient ID	CD8 T Cells	T Cells gamma delta	Sub-type	ER IHC	ER Status	PR Status	HER2 Status	HER2 SNP6	Tumor Stage	Age At Diag-nosis	In-ferred Meno-pause	Che-mo-ther-apy	LN+	OS Sta-tus	OS Months
MB-0420	0.007	0.017	M	Neg	Neg	Neg	Neg	Neutral	1	34.92	Pre	Y	Neg	0	76.73
MB-0436	0.000	0.042	BL2	Neg	Neg	Neg	Neg	Neutral	1	88.29	Post	N	Neg	1	75.5
MB-0446	0.005	0.021	BL2	Neg	Neg	Neg	Neg	Neutral	2	56.96	Post	Y	Neg	0	72.27
MB-0464	0.055	0.149	LAR	Neg	Neg	Neg	Neg	Loss	3	58.98	Post	Y	Pos	1	39.3
MB-0481	0.158	0.103	M	Neg	Neg	Neg	Neg	Neutral	1	54.22	Post	Y	Neg	1	36.4
MB-0494	0.094	0.000	BL2	Neg	Neg	Neg	Neg	Neutral	3	77.22	Post	Y	Pos	1	36.63
MB-0516	0.031	0.000	M	Neg	Neg	Neg	Neg	Neutral	1	62.81	Post	Y	Neg	0	114.47
MB-0525	0.000	0.000	BL2	Neg	Neg	Neg	Neg	Neutral	2	48.47	Pre	Y	Neg	0	65.87
MB-0581	0.028	0.000	BL2	Neg	Neg	Neg	Neg	Neutral	2	96.29	Post	N	Pos	1	7.87
MB-0588	0.041	0.024	LAR	Neg	Neg	Neg	Neg	Neutral	2	76.35	Post	N	Neg	0	119.73
MB-0627	0.081	0.147	MSL	Neg	Neg	Neg	Neg	Neutral	NA	54.1	Post	N	Neg	0	0.77
MB-0639	0.119	0.032	M	Neg	Neg	Neg	Neg	Neutral	1	50.39	Post	Y	Neg	0	75.4
MB-0658	0.174	0.139	BL2	Neg	Neg	Neg	Neg	Loss	2	60.89	Post	Y	Pos	0	97.27
MB-0664	0.042	0.004	BL1	Neg	Neg	Neg	Neg	Neutral	3	78.27	Post	Y	Pos	1	11.07
MB-0869	0.058	0.083	BL1	Neg	Neg	Neg	Neg	Neutral	2	58.83	Post	Y	Neg	1	112.4
MB-0874	0.107	0.061	BL1	Neg	Neg	Neg	Neg	Neutral	2	59.22	Post	Y	Neg	1	16.6
MB-0901	0.078	0.212	BL1	Neg	Neg	Neg	Neg	Neutral	2	68.19	Post	Y	Pos	0	136.17
MB-2724	0.123	0.155	IM	Neg	Neg	Neg	Neg	Neutral	1	60.59	Post	N	Neg	1	108.07
MB-2827	0.103	0.079	UNS	Neg	Neg	Neg	Neg	Neutral	2	36.5	Pre	Y	Pos	0	235.67
MB-2842	0.011	0.000	IM	Neg	Neg	Neg	Neg	Neutral	2	58.48	Post	Y	Pos	1	19.73
MB-2849	0.116	0.100	IM	Neg	Neg	Neg	Neg	Neutral	1	60.65	Post	N	Neg	1	43.2
MB-2850	0.137	0.111	IM	Neg	Neg	Neg	Neg	Neutral	3	31.48	Pre	Y	Pos	1	9.43
MB-2857	0.000	0.183	IM	Neg	Neg	Neg	Neg	Neutral	2	45.3	Pre	Y	Pos	0	250.67
MB-2904	0.221	0.000	IM	Neg	Neg	Neg	Neg	Neutral	2	34.83	Pre	Y	Pos	1	125.6

METABRIC: Molecular Taxonomy of Breast Cancer International Consortium; TNBC: triple negative breast cancer; High quantity of T Cells: > 0.25*standard deviation; Low quantity of T Cells: < 0.25*standard deviation; ER: estrogen receptor; IHC: immunohistochemistry; Pos: Positive; Neg: negative; PR: progesterone receptor; HER2: human epidermal growth factor receptor 2; LN +: Lymph Node Positive; Y: Yes; N: No; OS: overall survival; DFS: disease free survival; BL1: basal-like 1; BL2: basal-like 2; IM: immunomodulatory; M: mesenchymal, MSL: mesenchymal stem-like, LAR: luminal androgen receptor; UNS: unstable; NA: not applicable

Table S11: 199 METABRIC TNBC Cases

Patient ID	CD8 T Cells	T Cells gamma delta	Sub-type	ER IHC	ER Status	PR Status	HER2 Status	HER2 SNP6	Tumor Stage	Age At Diag-nosis	In-ferred Meno-pause	Che-mo-ther-apy	LN+	OS Sta-tus	OS Months
MB-2912	0.223	0.114	BL1	Neg	Neg	Neg	Neg	Neutral	3	59.6	Post	Y	Pos	0	267.4
MB-2917	0.002	0.124	UNS	Neg	Neg	Neg	Neg	Neutral	2	33.9	Pre	Y	Pos	1	31.93
MB-2929	0.003	0.096	LAR	Neg	Neg	Neg	Neg	Neutral	2	53.31	Post	Y	Pos	1	262.87
MB-2957	0.027	0.079	UNS	Neg	Neg	Neg	Neg	Neutral	2	31.02	Pre	Y	Pos	0	262.13
MB-2993	0.090	0.048	NA	Neg	Neg	Neg	Neg	Neutral	1	63.65	Post	N	Neg	0	187.03
MB-3001	0.064	0.029	BL1	Neg	Neg	Neg	Neg	Loss	2	50.45	Post	Y	Pos	0	263.23
MB-3014	0.079	0.076	BL1	Neg	Neg	Neg	Neg	Neutral	2	39.7	Pre	Y	Pos	0	262.63
MB-3046	0.058	0.036	M	Neg	Neg	Neg	Neg	Neutral	1	67.2	Post	N	Neg	1	73.83
MB-3057	0.049	0.000	BL1	Neg	Neg	Neg	Neg	Neutral	2	55.3	Post	Y	Pos	1	32.03
MB-3058	0.000	0.078	UNS	Neg	Neg	Neg	Neg	Neutral	2	47.74	Pre	Y	Pos	0	254.27
MB-3062	0.136	0.076	MSL	Neg	Neg	Neg	Neg	Neutral	1	62.87	Post	N	Neg	0	146.37
MB-3063	0.000	0.059	M	Neg	Neg	Neg	Neg	Neutral	3	43.51	Pre	Y	Pos	1	28.57
MB-3153	0.000	0.083	LAR	Neg	Neg	Neg	Neg	Neutral	2	42.17	Pre	Y	Pos	0	227.93
MB-3211	0.090	0.053	IM	Neg	Neg	Neg	Neg	Neutral	1	58.31	Post	N	Neg	0	145.5
MB-3218	0.001	0.211	BL1	Neg	Neg	Neg	Neg	Neutral	2	44.94	Pre	Y	Pos	0	248.77
MB-3271	0.000	0.000	IM	Neg	Neg	Neg	Neg	Neutral	1	59.43	Post	N	Neg	1	65.47
MB-3277	0.083	0.000	IM	Neg	Neg	Neg	Neg	Neutral	2	29.92	Pre	Y	Pos	1	32.93
MB-3292	0.076	0.036	IM	Neg	Neg	Neg	Neg	Neutral	1	60.04	Post	N	Neg	0	217.77
MB-3297	0.012	0.037	NA	Neg	Neg	Neg	Neg	Neutral	2	42.72	Pre	Y	Pos	0	236.07
MB-3383	0.000	0.054	UNS	Neg	Neg	Neg	Neg	Neutral	3	38.49	Pre	Y	Pos	1	23.2
MB-3395	0.105	0.122	MSL	Neg	Neg	Neg	Neg	Neutral	1	52.45	Post	N	Neg	0	243.77
MB-3396	0.000	0.103	BL2	Neg	Neg	Neg	Neg	Neutral	2	59.29	Post	Y	Pos	0	226.73
MB-3453	0.064	0.038	UNS	Neg	Neg	Neg	Neg	Neutral	2	51.36	Post	Y	Pos	1	32.83
MB-3500	0.000	0.046	UNS	Neg	Neg	Neg	Neg	Neutral	1	66.91	Post	N	Neg	0	239.3

METABRIC: Molecular Taxonomy of Breast Cancer International Consortium; TNBC: triple negative breast cancer; High quantity of T Cells: > 0.25*standard deviation; Low quantity of T Cells: < 0.25*standard deviation; ER: estrogen receptor; IHC: immunohistochemistry; Pos: Positive; Neg: negative; PR: progesterone receptor; HER2: human epidermal growth factor receptor 2; LN +: Lymph Node Positive; Y: Yes; N: No; OS: overall survival; DFS: disease free survival; BL1: basal-like 1; BL2: basal-like 2; IM: immunomodulatory; M: mesenchymal, MSL: mesenchymal stem-like, LAR: luminal androgen receptor; UNS: unstable; NA: not applicable

Table S11: 199 METABRIC TNBC Cases

Patient ID	CD8 T Cells	T Cells gamma delta	Sub-type	ER IHC	ER Status	PR Status	HER2 Status	HER2 SNP6	Tumor Stage	Age At Diag-nosis	In-ferred Meno-pause	Che-mo-ther-apy	LN+	OS Sta-tus	OS Months
MB-3502	0.097	0.102	UNS	Neg	Neg	Neg	Neg	Neutral	2	50.79	Post	Y	Pos	0	229.33
MB-3702	0.016	0.157	MSL	Neg	Neg	Neg	Neg	Neutral	2	37.16	Pre	Y	Pos	0	230.47
MB-3706	0.019	0.000	BL1	Neg	Neg	Neg	Neg	Neutral	1	55.71	Post	N	Neg	1	38.8
MB-4015	0.043	0.023	UNS	Neg	Neg	Neg	Neg	Loss	NA	76.13	Post	N	Neg	1	11.3
MB-4024	0.030	0.000	M	Neg	Neg	Neg	Neg	Loss	2	56.14	Post	N	Neg	0	124.1
MB-4146	0.261	0.000	IM	Neg	Neg	Neg	Neg	Neutral	2	43.06	Pre	Y	Pos	1	15.63
MB-4303	0.206	0.000	NA	Neg	Neg	Neg	Neg	Neutral	1	66.91	Post	N	Neg	1	89.03
MB-4332	0.214	0.000	BL1	Neg	Neg	Neg	Neg	Neutral	1	34.36	Pre	N	Neg	0	307.93
MB-4407	0.000	0.037	M	Neg	Neg	Neg	Neg	Neutral	1	44.4	Pre	N	Neg	1	83.37
MB-4408	0.154	0.003	UNS	Neg	Neg	Neg	Neg	Neutral	NA	71.66	Post	N	Neg	1	206.57
MB-4416	0.230	0.017	IM	Neg	Neg	Neg	Neg	Neutral	2	44.89	Pre	Y	Pos	1	241.6
MB-4417	0.000	0.101	BL2	Neg	Neg	Neg	Neg	Neutral	2	68.16	Post	N	Neg	1	16.7
MB-4621	0.000	0.036	UNS	Neg	Neg	Neg	Neg	Neutral	3	39.51	Pre	Y	Pos	0	271.87
MB-4622	0.000	0.251	UNS	Neg	Neg	Neg	Neg	Neutral	2	40.83	Pre	Y	Pos	1	282.83
MB-4660	0.147	0.000	UNS	Neg	Neg	Neg	Neg	Neutral	2	37.53	Pre	Y	Pos	1	39.87
MB-4679	0.160	0.005	IM	Neg	Neg	Neg	Neg	Neutral	1	64.11	Post	N	Neg	1	292.67
MB-4696	0.000	0.126	LAR	Neg	Neg	Neg	Neg	Loss	NA	49.98	Pre	N	Neg	0	255.27
MB-4707	0.102	0.015	UNS	Neg	Neg	Neg	Neg	Neutral	2	59.85	Post	N	Neg	0	221.2
MB-4715	0.095	0.041	UNS	Neg	Neg	Neg	Neg	Neutral	2	67.8	Post	Y	Pos	1	35.23
MB-4717	0.056	0.000	M	Neg	Neg	Neg	Neg	Loss	2	43.51	Pre	N	Neg	1	42.9
MB-4732	0.187	0.014	IM	Neg	Neg	Neg	Neg	Neutral	2	26.72	Pre	Y	Pos	0	149.87
MB-4733	0.236	0.000	IM	Neg	Neg	Neg	Neg	Neutral	2	56.27	Post	Y	Pos	1	19.73
MB-4757	0.029	0.035	BL1	Neg	Neg	Neg	Neg	Neutral	NA	38.93	Pre	Y	Pos	1	27.4
MB-4758	0.036	0.000	M	Neg	Neg	Neg	Neg	Neutral	NA	45.77	Pre	N	Neg	0	211.93

METABRIC: Molecular Taxonomy of Breast Cancer International Consortium; TNBC: triple negative breast cancer; High quantity of T Cells: > 0.25*standard deviation; Low quantity of T Cells: < 0.25*standard deviation; ER: estrogen receptor; IHC: immunohistochemistry; Pos: Positive; Neg: negative; PR: progesterone receptor; HER2: human epidermal growth factor receptor 2; LN +: Lymph Node Positive; Y: Yes; N: No; OS: overall survival; DFS: disease free survival; BL1: basal-like 1; BL2: basal-like 2; IM: immunomodulatory; M: mesenchymal, MSL: mesenchymal stem-like, LAR: luminal androgen receptor; UNS: unstable; NA: not applicable

Table S11: 199 METABRIC TNBC Cases

Patient ID	CD8 T Cells	T Cells gamma delta	Sub-type	ER IHC	ER Status	PR Status	HER2 Status	HER2 SNP6	Tumor Stage	Age At Diag-nosis	In-ferred Meno-pause	Che-mo-ther-apy	LN+	OS Sta-tus	OS Months
MB-4769	0.075	0.015	BL1	Neg	Neg	Neg	Neg	Neutral	2	65.86	Post	N	Neg	1	21.7
MB-4792	0.026	0.019	M	Neg	Neg	Neg	Neg	Neutral	2	49.92	Pre	Y	Pos	1	40.63
MB-4793	0.027	0.036	IM	Neg	Neg	Neg	Neg	Neutral	2	72.49	Post	N	Neg	1	83.37
MB-4865	0.173	0.000	UNS	Neg	Neg	Neg	Neg	Neutral	2	50.7	Post	N	Neg	0	229.9
MB-4881	0.280	0.000	M	Neg	Neg	Neg	Neg	Neutral	NA	37.05	Pre	N	Neg	0	274.2
MB-4888	0.078	0.012	MSL	Neg	Neg	Neg	Neg	Neutral	1	63.95	Post	N	Neg	0	230.5
MB-4893	0.280	0.084	IM	Neg	Neg	Neg	Neg	Neutral	2	33.04	Pre	Y	Pos	0	176.7
MB-4911	0.072	0.000	BL1	Neg	Neg	Neg	Neg	Neutral	2	43.51	Pre	Y	Pos	1	31.43
MB-4931	0.338	0.215	IM	Neg	Neg	Neg	Neg	Neutral	2	58.39	Post	N	Neg	1	30.37
MB-4938	0.016	0.000	M	Neg	Neg	Neg	Neg	Neutral	2	33.92	Pre	Y	Pos	1	70.6
MB-4945	0.093	0.030	M	Neg	Neg	Neg	Neg	Neutral	NA	65.53	Post	Y	Pos	1	20.13
MB-4974	0.115	0.125	BL1	Neg	Neg	Neg	Neg	Neutral	1	65.16	Post	N	Neg	1	176.1
MB-4993	0.000	0.036	LAR	Neg	Neg	Neg	Neg	Neutral	2	33.55	Pre	Y	Pos	1	75.23
MB-5008	0.035	0.026	NA	Neg	Neg	Neg	Neg	Neutral	3	65.76	Post	Y	Pos	0	223.3
MB-5057	0.126	0.035	UNS	Neg	Neg	Neg	Neg	Neutral	1	39.25	Pre	N	Neg	1	56.33
MB-5070	0.015	0.062	IM	Neg	Neg	Neg	Neg	Loss	NA	47.38	Pre	Y	Pos	0	213.9
MB-5072	0.041	0.058	LAR	Neg	Neg	Neg	Neg	Neutral	2	52.23	Post	Y	Pos	1	50.23
MB-5076				Neg	Neg	Neg	Neg	Neutral	2	28.05	Pre	Y		1	26.87
MB-5100	0.137	0.000	IM	Neg	Neg	Neg	Neg	Neutral	2	32.74	Pre	Y	Pos	1	19.9
MB-5102	0.007	0.068	M	Neg	Neg	Neg	Neg	Neutral	2	69.93	Post	Y	Pos	1	30.37
MB-5115	0.000	0.043	BL1	Neg	Neg	Neg	Neg	Neutral	2	40.57	Pre	Y	Pos	0	77.4
MB-5126	0.073	0.076	BL1	Neg	Neg	Neg	Neg	Neutral	NA	63.48	Post	Y	Pos	1	27.3
MB-5135	0.006	0.009	M	Neg	Neg	Neg	Neg	Neutral	2	38.59	Pre	N	Neg	1	37.37
MB-5138	0.085	0.021	IM	Neg	Neg	Neg	Neg	Neutral	NA	44.59	Pre	Y	Pos	1	37
MB-5155	0.050	0.067	NA	Neg	Neg	Neg	Neg	Neutral	2	51.08	Post	Y	Pos	0	259.93

METABRIC: Molecular Taxonomy of Breast Cancer International Consortium; TNBC: triple negative breast cancer; High quantity of T Cells: > 0.25*standard deviation; Low quantity of T Cells: < 0.25*standard deviation; ER: estrogen receptor; IHC: immunohistochemistry; Pos: Positive; Neg: negative; PR: progesterone receptor; HER2: human epidermal growth factor receptor 2; LN +: Lymph Node Positive; Y: Yes; N: No; OS: overall survival; DFS: disease free survival; BL1: basal-like 1; BL2: basal-like 2; IM: immunomodulatory; M: mesenchymal, MSL: mesenchymal stem-like, LAR: luminal androgen receptor; UNS: unstable; NA: not applicable

Table S11: 199 METABRIC TNBC Cases

Patient ID	CD8 T Cells	T Cells gamma delta	Sub-type	ER IHC	ER Status	PR Status	HER2 Status	HER2 SNP6	Tumor Stage	Age At Diag-nosis	In-ferred Meno-pause	Che-mo-ther-apy	LN+	OS Sta-tus	OS Months
MB-5173	0.019	0.039	M	Neg	Neg	Neg	Neg	Neutral	2	69.05	Post	N	Neg	1	22.13
MB-5208	0.039	0.088	M	Neg	Neg	Neg	Neg	Neutral	2	52.43	Post	N	Neg	0	151.9
MB-5209	0.248	0.038	IM	Neg	Neg	Neg	Neg	Neutral	2	79.17	Post	N	Neg	1	175.1
MB-5223	0.231	0.067	MSL	Neg	Neg	Neg	Neg	Neutral	2	42.05	Pre	Y	Pos	1	144.67
MB-5225	0.339	0.220	UNS	Neg	Neg	Neg	Neg	Neutral	2	68.01	Post	N	Neg	1	43.27
MB-5232	0.145	0.084	MSL	Neg	Neg	Neg	Neg	Neutral	1	74.53	Post	N	Neg	1	211.2
MB-5236	0.123	0.000	BL1	Neg	Neg	Neg	Neg	Neutral	3	63.02	Post	Y	Pos	0	166.67
MB-5258	0.029	0.000	BL1	Neg	Neg	Neg	Neg	Neutral	2	72.77	Post	N	Neg	1	101.07
MB-5281	0.000	0.000	LAR	Neg	Neg	Neg	Neg	Neutral	1	55.72	Post	N	Neg	0	79.3
MB-5295	0.129	0.000	M	Neg	Neg	Neg	Neg	Neutral	2	60.51	Post	N	Neg	1	75.7
MB-5323	0.346	0.000	IM	Neg	Neg	Neg	Neg	Loss	1	31.26	Pre	N	Neg	0	226.07
MB-5335	0.058	0.015	M	Neg	Neg	Neg	Neg	Neutral	2	41.81	Pre	N	Neg	1	25.23
MB-5346	0.077	0.031	BL1	Neg	Neg	Neg	Neg	Neutral	NA	52.09	Post	Y	Pos	0	194.3
MB-5348	0.066	0.102	IM	Neg	Neg	Neg	Neg	Neutral	2	41.27	Pre	Y	Pos	0	169.67
MB-5421	0.000	0.040	M	Neg	Neg	Neg	Neg	Neutral	1	68	Post	N	Neg	0	194.57
MB-5427	0.162	0.000	M	Neg	Neg	Neg	Neg	Neutral	2	83.68	Post	N	Neg	1	155.73
MB-5440	0.113	0.077	IM	Neg	Neg	Neg	Neg	Neutral	2	49.05	Pre	Y	Pos	0	196.63
MB-5446	0.000	0.000	LAR	Neg	Neg	Neg	Neg	Neutral	2	51.25	Post	Y	Pos	1	162.83
MB-5450	0.083	0.057	IM	Neg	Neg	Neg	Neg	Neutral	1	38.92	Pre	N	Neg	0	190.2
MB-5465	0.023	0.047	M	Neg	Neg	Neg	Neg	Loss	2	69.83	Post	N	Neg	1	18.8
MB-5526	0.040	0.102	UNS	Neg	Neg	Neg	Neg	Neutral	1	45.42	Pre	N	Neg	0	151.07
MB-5529	0.135	0.005	BL1	Neg	Neg	Neg	Neg	Neutral	2	40.21	Pre	Y	Pos	1	14.8
MB-5531	0.065	0.014	BL2	Neg	Neg	Neg	Neg	Neutral	1	66.56	Post	N	Neg	0	183.97
MB-5547	0.018	0.060	M	Neg	Neg	Neg	Neg	Neutral	2	43.08	Pre	Y	Pos	0	98.57

METABRIC: Molecular Taxonomy of Breast Cancer International Consortium; TNBC: triple negative breast cancer; High quantity of T Cells: > 0.25*standard deviation; Low quantity of T Cells: < 0.25*standard deviation; ER: estrogen receptor; IHC: immunohistochemistry; Pos: Positive; Neg: negative; PR: progesterone receptor; HER2: human epidermal growth factor receptor 2; LN +: Lymph Node Positive; Y: Yes; N: No; OS: overall survival; DFS: disease free survival; BL1: basal-like 1; BL2: basal-like 2; IM: immunomodulatory; M: mesenchymal, MSL: mesenchymal stem-like, LAR: luminal androgen receptor; UNS: unstable; NA: not applicable

Table S11: 199 METABRIC TNBC Cases

Patient ID	CD8 T Cells	T Cells gamma delta	Sub-type	ER IHC	ER Status	PR Status	HER2 Status	HER2 SNP6	Tumor Stage	Age At Diag-nosis	In-ferred Meno-pause	Che-mo-ther-apy	LN+	OS Sta-tus	OS Months
MB-5548	0.021	0.058	IM	Neg	Neg	Neg	Neg	Neutral	NA	62.43	Post	N	Neg	0	25.33
MB-5551	0.000	0.000	M	Neg	Neg	Neg	Neg	Neutral	2	67.24	Post	N	Neg	0	182.23
MB-5560	0.061	0.000	MSL	Neg	Neg	Neg	Neg	Loss	1	44.05	Pre	N	Neg	0	186.6
MB-5566	0.044	0.000	MSL	Neg	Neg	Neg	Neg	Neutral	NA	38.44	Pre	Y	Pos	0	234.4
MB-5572	0.142	0.114	IM	Neg	Neg	Neg	Neg	Neutral	1	47.88	Pre	N	Neg	0	178.63
MB-5602	0.098	0.126	IM	Neg	Neg	Neg	Neg	Neutral	NA	39.7	Pre	Y	Pos	0	224.43
MB-5616	0.136	0.000	IM	Neg	Neg	Neg	Neg	Neutral	2	41.53	Pre	Y	Pos	0	180.63
MB-5624	0.000	0.058	UNS	Neg	Neg	Neg	Neg	Neutral	NA	55.43	Post	Y	Pos	0	136
MB-5625				Neg	Neg	Neg	Neg	Neutral	NA	45.51	Pre	Y		1	32.3
MB-5633	0.109	0.000	IM	Neg	Neg	Neg	Neg	Neutral	3	62.7	Post	Y	Pos	1	35.03
MB-5634	0.041	0.011	IM	Neg	Neg	Neg	Neg	Neutral	2	55.53	Post	N	Neg	1	62.9
MB-5655	0.000	0.038	UNS	Neg	Neg	Neg	Neg	Neutral	2	67.57	Post	N	Neg	0	191.8
MB-6052	0.111	0.177	NA	Neg	Neg	Neg	Neg	Neutral	NA	36.47	Pre	N	Neg	0	255.37
MB-6058	0.210	0.055	IM	Neg	Neg	Neg	Neg	Neutral	NA	41.79	Pre	Y	Pos	1	22.67
MB-6098	0.096	0.000	UNS	Neg	Neg	Neg	Neg	Neutral	NA	28.29	Pre	Y	Pos	1	10.63
MB-6143	0.000	0.025	M	Neg	Neg	Neg	Neg	Neutral	2	60.6	Post	N	Neg	0	255.6
MB-6178	0.103	0.007	UNS	Neg	Neg	Neg	Neg	Neutral	NA	38.58	Pre	N	Pos	1	33.13
MB-6242	0.189	0.000	BL2	Neg	Neg	Neg	Neg	Neutral	NA	41.29	Pre	Y	Pos	0	165.43
MB-6245	0.000	0.078	M	Neg	Neg	Neg	Neg	Neutral	NA	47.01	Pre	N	Neg	0	177.63
MB-6248	0.000	0.095	BL1	Neg	Neg	Neg	Neg	Neutral	NA	51.22	Post	Y	Pos	0	182.83
MB-6251	0.048	0.014	M	Neg	Neg	Neg	Neg	Loss	2	48.59	Pre	Y	Pos	1	14.7
MB-6272	0.022	0.008	UNS	Neg	Neg	Neg	Neg	Neutral	NA	36.93	Pre	Y	Pos	1	22.4
MB-6280	0.009	0.026	BL1	Neg	Neg	Neg	Neg	Neutral	NA	53.68	Post	Y	Pos	1	27.87
MB-6336	0.182	0.000	UNS	Neg	Neg	Neg	Neg	Neutral	NA	63.22	Post	Y	Pos	1	17.2

METABRIC: Molecular Taxonomy of Breast Cancer International Consortium; TNBC: triple negative breast cancer; High quantity of T Cells: > 0.25*standard deviation; Low quantity of T Cells: < 0.25*standard deviation; ER: estrogen receptor; IHC: immunohistochemistry; Pos: Positive; Neg: negative; PR: progesterone receptor; HER2: human epidermal growth factor receptor 2; LN +: Lymph Node Positive; Y: Yes; N: No; OS: overall survival; DFS: disease free survival; BL1: basal-like 1; BL2: basal-like 2; IM: immunomodulatory; M: mesenchymal, MSL: mesenchymal stem-like, LAR: luminal androgen receptor; UNS: unstable; NA: not applicable

Table S11: 199 METABRIC TNBC Cases

Patient ID	CD8 T Cells	T Cells gamma delta	Sub-type	ER IHC	ER Status	PR Status	HER2 Status	HER2 SNP6	Tumor Stage	Age At Diag-nosis	In-ferred Meno-pause	Che-mo-ther-apy	LN+	OS Sta-tus	OS Months
MB-7008	0.113	0.036	LAR	Neg	Neg	Neg	Neg	Neutral	NA	69.94	Post	N	Neg	1	50.9
MB-7009	0.023	0.021	M	Neg	Neg	Neg	Neg	Neutral	NA	68.28	Post	Y	Pos	1	54.77
MB-7012	0.029	0.027	BL1	Neg	Neg	Neg	Neg	Neutral	NA	60.96	Post	Y	Neg	1	19.83
MB-7023	0.128	0.067	BL2	Neg	Neg	Neg	Neg	Neutral	NA	66.66	Post	Y	Pos	1	27.97
MB-7025	0.076	0.029	MSL	Neg	Neg	Neg	Neg	Neutral	NA	65.22	Post	N	Neg	0	80.23
MB-7031	0.125	0.000	NA	Neg	Neg	Neg	Neg	Neutral	NA	60.58	Post	Y	Pos	0	85.37
MB-7036	0.127	0.070	BL1	Neg	Neg	Neg	Neg	Neutral	NA	54.48	Post	Y	Pos	0	120.43
MB-7038	0.037	0.090	M	Neg	Neg	Neg	Neg	Neutral	NA	57.62	Post	Y	Pos	0	81.03
MB-7045	0.169	0.000	LAR	Neg	Neg	Neg	Neg	Neutral	NA	61.39	Post	N	Neg	1	94.93
MB-7049	0.285	0.000	M	Neg	Neg	Neg	Neg	Neutral	NA	48.75	Pre	N	Neg	0	102.03
MB-7052	0.109	0.127	IM	Neg	Neg	Neg	Neg	Neutral	NA	45.45	Pre	N	Neg	0	106.13
MB-7055	0.005	0.008	M	Neg	Neg	Neg	Neg	Neutral	NA	54.87	Post	N	Neg	1	9.13
MB-7057	0.046	0.069	NA	Neg	Neg	Neg	Neg	Neutral	NA	72.81	Post	Y	Pos	1	43.83
MB-7078	0.108	0.000	BL2	Neg	Neg	Neg	Neg	Neutral	NA	74.8	Post	N	Neg	1	4.17
MB-7081	0.111	0.126	IM	Neg	Neg	Neg	Neg	Neutral	NA	69.12	Post	N	Neg	0	140.23
MB-7087	0.044	0.000	NA	Neg	Neg	Neg	Neg	Neutral	NA	66.61	Post	Y	Pos	1	5.07
MB-7090	0.049	0.015	BL2	Neg	Neg	Neg	Neg	Neutral	NA	48.52	Pre	Y	Pos	0	109.77
MB-7114	0.041	0.056	BL1	Neg	Neg	Neg	Neg	Neutral	NA	55.57	Post	N	Neg	0	123.27
MB-7119	0.031	0.144	IM	Neg	Neg	Neg	Neg	Neutral	NA	54.53	Post	Y	Pos	0	128.37
MB-7121	0.097	0.108	IM	Neg	Neg	Neg	Neg	Neutral	NA	49.02	Pre	Y	Pos	1	88.83
MB-7145	0.035	0.054	BL1	Neg	Neg	Neg	Neg	Neutral	NA	44.42	Pre	Y	Pos	0	138.33
MB-7154	0.308	0.027	IM	Neg	Neg	Neg	Neg	Neutral	NA	77.54	Post	N	Neg	1	108.47
MB-7155	0.061	0.074	UNS	Neg	Neg	Neg	Neg	Neutral	NA	41.14	Pre	Y	Pos	0	149.77
MB-7158	0.065	0.032	IM	Neg	Neg	Neg	Neg	Neutral	NA	50.73	Post	Y	Pos	1	46.43

METABRIC: Molecular Taxonomy of Breast Cancer International Consortium; TNBC: triple negative breast cancer; High quantity of T Cells: > 0.25*standard deviation; Low quantity of T Cells: < 0.25*standard deviation; ER: estrogen receptor; IHC: immunohistochemistry; Pos: Positive; Neg: negative; PR: progesterone receptor; HER2: human epidermal growth factor receptor 2; LN +: Lymph Node Positive; Y: Yes; N: No; OS: overall survival; DFS: disease free survival; BL1: basal-like 1; BL2: basal-like 2; IM: immunomodulatory; M: mesenchymal, MSL: mesenchymal stem-like, LAR: luminal androgen receptor; UNS: unstable; NA: not applicable

Table S11: 199 METABRIC TNBC Cases

Patient ID	CD8 T Cells	T Cells gamma delta	Sub-type	ER IHC	ER Status	PR Status	HER2 Status	HER2 SNP6	Tumor Stage	Age At Diag-nosis	In-ferred Meno-pause	Che-mo-ther-apy	LN+	OS Sta-tus	OS Months
MB-7159	0.000	0.000	M	Neg	Neg	Neg	Neg	Neutral	NA	60.45	Post	Y	Neg	1	21.93
MB-7165	0.021	0.020	BL1	Neg	Neg	Neg	Neg	Neutral	NA	28.78	Pre	Y	Pos	0	147.17
MB-7205	0.070	0.042	IM	Neg	Neg	Neg	Neg	Neutral	NA	38.17	Pre	Y	Pos	0	166.03
MB-7208	0.160	0.071	LAR	Neg	Neg	Neg	Neg	Neutral	NA	48.83	Pre	Y	Pos	1	45.93
MB-7258	0.209	0.000	LAR	Neg	Neg	Neg	Neg	Neutral	NA	45.11	Pre	Y	Pos	1	15.07
MB-7270	0.000	0.187	LAR	Neg	Neg	Neg	Neg	Neutral	NA	50.84	Post	Y	Pos	0	175.17

METABRIC: Molecular Taxonomy of Breast Cancer International Consortium; TNBC: triple negative breast cancer; High quantity of T Cells: > 0.25*standard deviation; Low quantity of T Cells: < 0.25*standard deviation; ER: estrogen receptor; IHC: immunohistochemistry; Pos: Positive; Neg: negative; PR: progesterone receptor; HER2: human epidermal growth factor receptor 2; LN +: Lymph Node Positive; Y: Yes; N: No; OS: overall survival; DFS: disease free survival; BL1: basal-like 1; BL2: basal-like 2; IM: immunomodulatory; M: mesenchymal, MSL: mesenchymal stem-like, LAR: luminal androgen receptor; UNS: unstable; NA: not applicable
